# Highly oriented hydrogels for tissue regeneration: design strategies, cellular mechanisms, and biomedical applications

**DOI:** 10.7150/thno.89493

**Published:** 2024-02-24

**Authors:** Jiuping Wu, Zhihe Yun, Wenlong Song, Tao Yu, Wu Xue, Qinyi Liu, Xinzhi Sun

**Affiliations:** 1 Department of Orthopedics, The First Affiliated Hospital of Zhengzhou University, Zhengzhou 450052, China.; 2 Translational Medicine Center, The First Affiliated Hospital of Zhengzhou University, Zhengzhou 450052, China.; 3 Department of Orthopedics, The Second Hospital of Jilin University, Changchun 130041, China.; 4 State Key Laboratory of Supramolecular Structure and Materials, College of Chemistry, Jilin University, Changchun 130023, China.

## Abstract

Many human tissues exhibit a highly oriented architecture that confers them with distinct mechanical properties, enabling adaptation to diverse and challenging environments. Hydrogels, with their water-rich "soft and wet" structure, have emerged as promising biomimetic materials in tissue engineering for repairing and replacing damaged tissues and organs. Highly oriented hydrogels can especially emulate the structural orientation found in human tissue, exhibiting unique physiological functions and properties absent in traditional homogeneous isotropic hydrogels. The design and preparation of highly oriented hydrogels involve strategies like including hydrogels with highly oriented nanofillers, polymer-chain networks, void channels, and microfabricated structures. Understanding the specific mechanism of action of how these highly oriented hydrogels affect cell behavior and their biological applications for repairing highly oriented tissues such as the cornea, skin, skeletal muscle, tendon, ligament, cartilage, bone, blood vessels, heart, etc., requires further exploration and generalization. Therefore, this review aims to fill that gap by focusing on the design strategy of highly oriented hydrogels and their application in the field of tissue engineering. Furthermore, we provide a detailed discussion on the application of highly oriented hydrogels in various tissues and organs and the mechanisms through which highly oriented structures influence cell behavior.

## 1. Introduction

Tissue engineering (TE), which was first defined in 1993, is an interdisciplinary field that combines the principles of life science and engineering in vitro with the ultimate goal of developing biological substitutes or entire tissues and organs for use in basic research on physiological processes and translational therapies aimed at repairing, preserving or enhancing tissue function 1. To achieve this, bionic scaffolds have been designed using various strategies to provide cells with biochemical and/or biophysical cues replicating the in vivo cellular survival environment, aiming to regulate cellular behavior toward proliferation and differentiation for tissue repair and regeneration. As such, emulating the structural and physicochemical properties of natural tissues or organs is paramount in TE.

Hydrogel, a cross-linked polymer network containing water, has emerged as a promising candidate for biomedical TE applications owing to its biocompatibility, ease of functionalization, and ability to mimic the chemical and biophysical properties of various structures in vivo 2-4. However, traditional hydrogels' poor mechanical properties and brittleness, which impede their wider use **(Table 1)**, have led researchers to explore and develop numerous approaches to address these limitations over the past decades. For instance, double-network 5, nanocomposite 6, dynamically cross-linked 7, and polyampholyte 8 hydrogels have shown great potential in improving the mechanical properties of hydrogels. However, as TE research advances, it has become clear that these efforts are insufficient to replicate natural tissues; replicating the structure of natural tissues and organs is also crucial in pursuing bionics.

**Figure 1:**
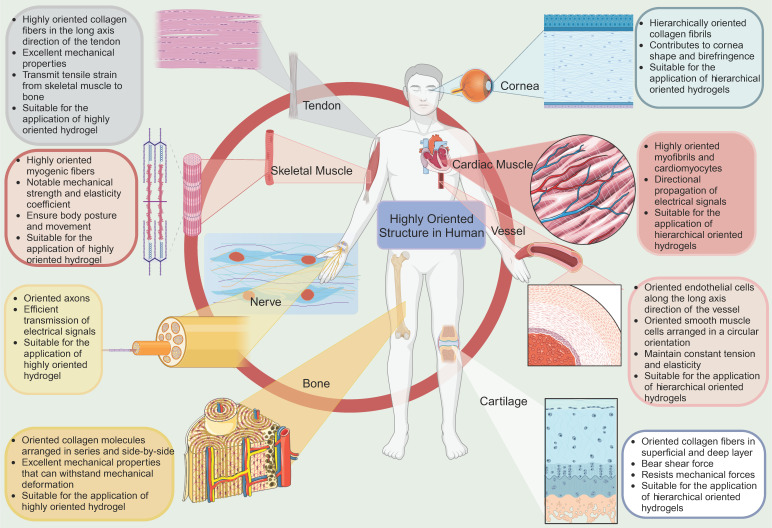
Highly oriented tissues and organs in the human body (Figure was created with Biorender.com).

Natural biological tissues such as cartilage 9, bone 10, skeletal muscle 11, tendons 12, myocardium 13, nerves 14, blood vessels 15, 16, and cornea 17 exhibit highly oriented structures that bestow them with distinctive mechanical properties, which are fundamental to their biological function** (Figure 1)**. For example, the layered, highly oriented cartilage structure confers different tasks to each layer, allowing them to work in synergy to dissipate compressive loads and absorb impact 9. Similarly, the highly oriented arrangement of collagen fibers in skeletal muscle imparts superior mechanical properties 11. The importance of highly oriented microenvironments in the body for regulating cell behavior and tissue development demonstrates the need to construct highly oriented 3D hydrogels in vitro to replicate natural tissues' unique mechanical properties and functions. This approach enhances hydrogels' mimicry of natural tissues and broadens their applications 18.

**Figure 2:**
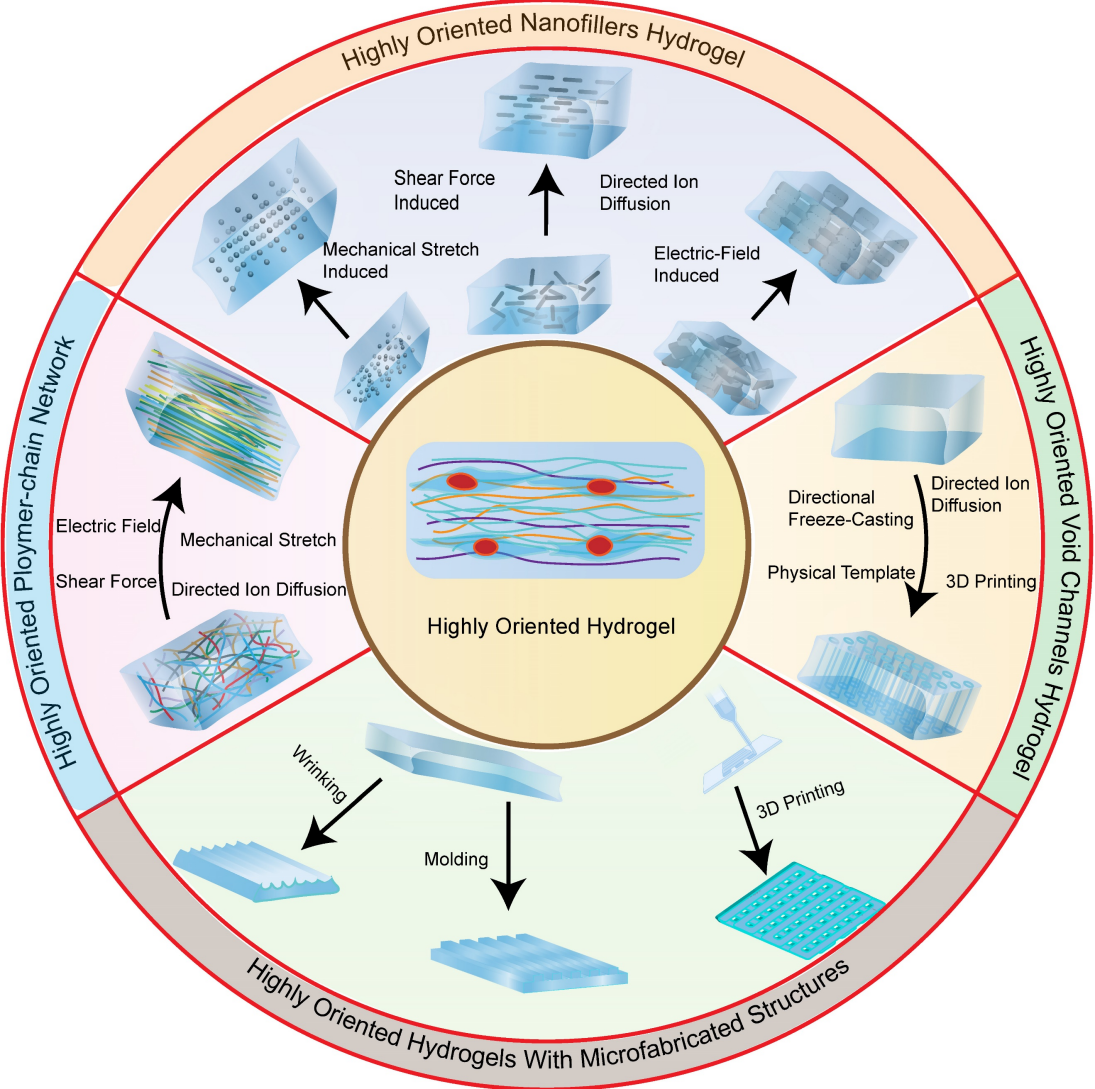
Classification of highly oriented hydrogels, which can be broadly summarized as highly oriented nanofillers hydrogels obtained using strategies such as magnetic/electric fields and mechanical force, highly oriented polymer-chain network hydrogels obtained by strategies such as mechanical force and ion diffusion, highly oriented void channels hydrogels prepared by strategies such as directed freezing/ice template, and highly oriented hydrogels with microfabricated structures using strategies such as 3D printing.

Recent studies have reported on a variety of highly oriented hydrogels, which can be broadly summarized as (i) highly oriented nanofillers hydrogels obtained using strategies such as magnetic/electric fields 19, 20 and mechanical forces 21, (ii) hydrogels with a highly oriented polymer-chain network obtained by strategies such as mechanical forces 22 and ion diffusion 23, (iii) hydrogels with highly oriented void channels prepared by strategies such as directed freezing/ice templates 24, and (iv) highly oriented hydrogels with microfabricated structures using strategies such as 3D printing 25, and (v) other highly oriented hydrogels prepared by special methods **(Figure 2)**. This paper aims to review each of these strategies, discussing their unique strengths and limitations and the progress of research.

In addition, although hydrogels have been widely used in TE and regenerative medicine, the specific mechanism of action of how hydrogels affect cell behavior is still not fully understood. Similarly, the mechanism of how highly oriented hydrogels affect specific cell behaviors and promote tissue repair and regeneration is unknown, with only limited literature suggesting that they can regulate specific cellular behaviors by influencing cell physiological processes such as cell adhesion, proliferation, migration, and differentiation 26, 27. Although their cytological mechanisms are not well understood, studies have confirmed that highly oriented hydrogels are effective for structural and functional tissue repair and regeneration, especially for highly oriented tissues such as nerve 28, skeletal muscle 29, tendon 30, ligament 31, cartilage 32, bone 33, blood vessels 34, heart 35, etc., by mimicking their structure. However, the extensive literature on the biological applications of highly oriented hydrogels remains to be summarized and reviewed.

Several exceptional previously published reviews exist on anisotropic hydrogels, ranging from comprehensive reviews covering their diverse applications across various fields to reports focusing on their use in a specific TE domain 36-41. Additionally, some reviews delve into specific preparation strategies for anisotropic hydrogels in TE applications 42, 43. Nancy Khuu et al. 44 reviewed the synthesis, characterization, and physical properties of anisotropic hydrogels for use in TE; however, they did not provide an overview of recent advances in TE applications. This review aims to fill that gap by focusing on the design strategy of highly oriented hydrogels and their application in TE **(Table 2)**. In particular, we provide a detailed discussion of the applications of highly oriented hydrogels in different tissues and organs and the mechanisms by which highly oriented structures may influence cell behavior. This comprehensive review provides valuable insights into the progress and challenges in preparing highly oriented hydrogels and their potential applications in TE. By understanding the strengths and weaknesses of each strategy, researchers can more effectively develop novel approaches to prepare highly oriented hydrogels with improved properties and functionalities, leading to better outcomes for TE applications.

## 2. Design and Preparation of Highly Oriented Hydrogels

Scientists have designed and reported several types of highly oriented structural hydrogels. These include nanofiller hydrogels obtained through magnetic/electrical fields, mechanical forces, self-assembly, and other methods; polymer-chain network hydrogels can be prepared through mechanical forces, ion diffusion, and other methods; void channel hydrogels can be obtained through sacrificial template methods such as directed freezing; hydrogels with microfabricated structures can be created through techniques such as 3D printing; and lastly, other specialized techniques can be used to prepare highly oriented hydrogels.

### 2.1. Highly Oriented Nanofillers Hydrogels

A common method for preparing highly oriented hydrogels is to disperse highly oriented nanofiller materials within them. Various methods, including magnetic/electrical fields, mechanical forces, directed freezing, and self-assembly, can control the distribution and orientation of the filler material. This section presents the principles and recent advances of these different methods and discusses their respective advantages and disadvantages.

#### 2.1.1. Magnetic-Field-Induced Highly Oriented Nanofillers Hydrogels

Magnetic highly oriented hydrogels are prepared by controlling the distribution of magnetic field-responsive materials in the hydrogel using a magnetic field to obtain an oriented structure. Magnetism-responsive electrons in the filler material allow for the formation of a highly oriented morphology in the external magnetic field, which is then fixed in the 3D hydrogel during the gelation process, resulting in a highly oriented structure that is maintained after the withdrawal of the external magnetic field 38. This composite hydrogel is highly oriented and magnetic, while also exhibiting excellent biocompatibility, which is particularly important in TE. Its preparation, involving magnetically responsive materials, both magnetic and non-magnetic, and an external magnetic field, offers a non-invasive and therefore advantageous alternative to other methods of creating highly oriented hydrogels.

##### 2.1.1.1. Magnetically Responsive Materials

Magnetically responsive materials can be categorized into two types: magnetic and non-magnetic materials. Among magnetic materials, magnetic nanoparticles (MNPs) are extensively employed for TE repair due to their remarkable magnetic responsiveness 42, 45-52. The MNPs used for biomedical applications include iron oxide-based MNPs such as hematite (α-Fe_2_O_3_), magnetite (γ-Fe_2_O_3_), and magnetite (Fe_3_O_4_), transition metal ferrites such as CoFe_2_O_4_, MnFe_2_O_4_, and transition metal alloys like FePT, which are incorporated into hydrogels 53-57. Iron oxide-based MNPs have been approved by the FDA for clinical use due to their good biocompatibility, high magnetization ability, low toxicity, easy preparation, and low cost. Consequently, they have gained significant attention in biomedical applications such as TE and drug delivery, and are more commonly used than other types of MNPs 42, 58-66.

The arrangement of MNPs into highly oriented hydrogels depends on various properties such as the size, shape, and saturation magnetic strength of MNPs, as well as the strength and shape of the magnetic field 67. These factors have a significant impact on the physicochemical properties and magnetic responsiveness of the final hydrogels, which in turn are influenced by the method of MNP synthesis 47, 59, 68. Currently, the three main synthesis methods for MNPs are physical, chemical, or biological 69. Chemical synthesis methods such as sol-gel, solvothermal synthesis, co-precipitation, and thermal decomposition account for the majority of all MNPs produced as they deliver better particle quality and higher homogeneity. Compared to chemical synthesis methods, physical synthesis methods including thermal evaporation, pulsed laser deposition and grinding produce less than 10% of all MNPs. Biological synthesis methods as the third synthesis method for MNPs include using microorganisms, enzymes, fungi, plants and plant extracts. Similarly, biological synthesis methods are also used for the preparation of only a small amount of MNPs 42, 58, 70. Among the chemical methods, the thermal decomposition method has better application potential than the simple, fast, cheap, and high-yield co-precipitation method, as it allows for better control over the shape and size of MNPs 42.

The size of MNPs is one of the most influential factors that affect their properties. When the particle diameter is less than a well-defined size (*D*_cr_), the particles become single domain and have uniform magnetization, regardless of the presence of an external magnetic field 42, 71. Further reduction in diameter to less than a certain critical size (*D*_sp_) results in particles entering the superparamagnetic regime, where the magnetic moment becomes thermally unstable and changes spontaneously and rapidly in the absence of an external magnetic field 72, 73. Superparamagnetic particles have zero magnetization in the absence of an external magnetic field and exhibit magnetic responsiveness only in the presence of such a field, making them highly remote-controllable in practical applications 74. Additionally, they have little tendency to form aggregates, which, along with their low toxicity, make highly oriented hydrogels of superparamagnetic state MNPs promising for TE applications 42, 70, 75.

**Figure 3:**
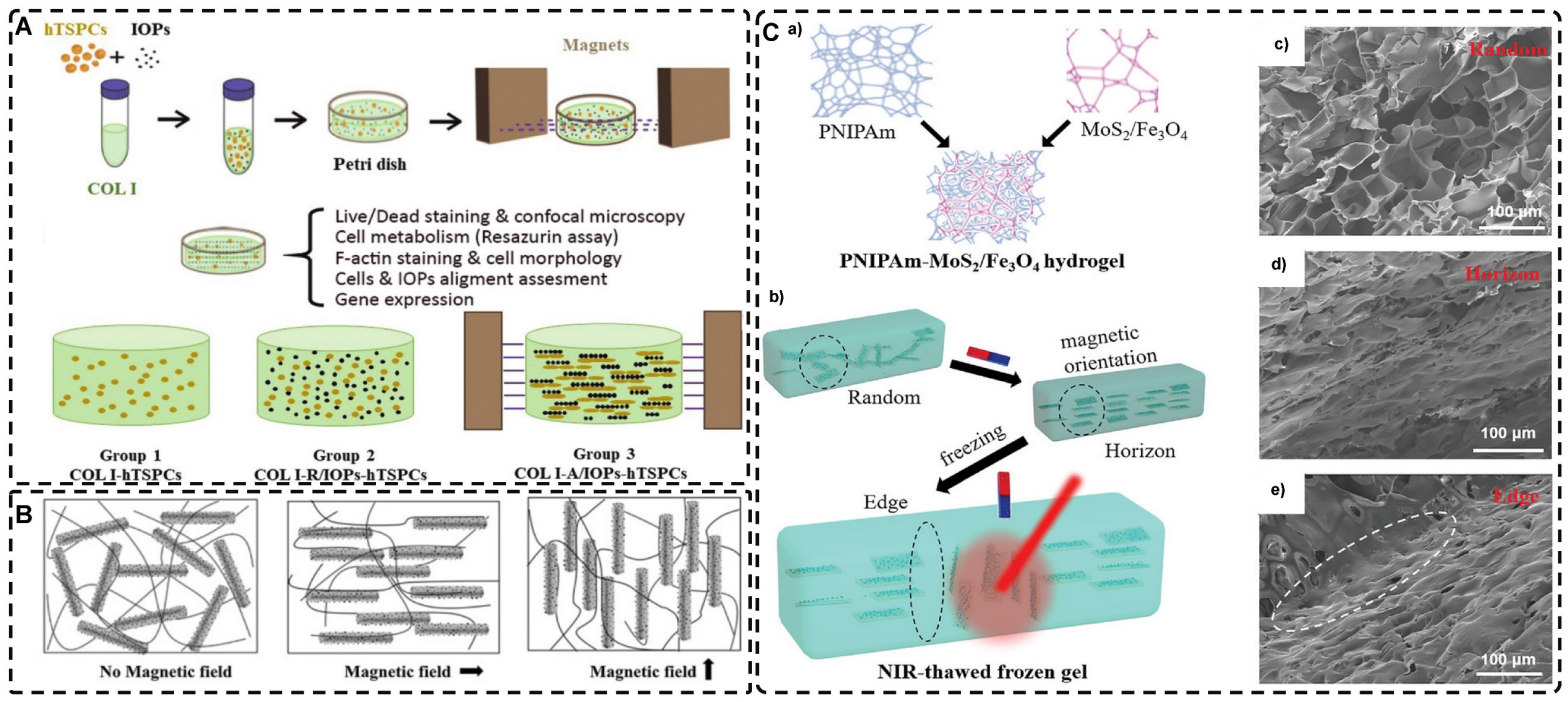
Fabrication of highly oriented hydrogels based on 0D nanoparticles (A), 1D nanotubes (B) and 2D nanosheets (C) in an external magnetic field. A). Schematic illustration of the formation of anisotropic hTSPC-nanocomposite hydrogel induced by the addition of paramagnetic iron oxide nanoparticles (IOPs) under exposure to magnetic field and cartoon of the three study groups. Adapted with permission from 19, copyright 2021, Royal Society of Chemistry. B) Schematic representation of 1D nanorods in hydrogel oriented under an external magnetic field. Adapted with permission from 81, copyright 2020, Elsevier. C) Schematic representation of a) the fabrication of PNIPAm-MoS_2_/Fe_3_O_4_ hydrogel and b) programming the orientation of MoS_2_/Fe_3_O_4_ under the magnetic field followed by NIR light-treatment of frozen hydrogels. Representative SEM images of PNIPAm-MoS_2_/Fe_3_O_4_ hydrogels under different conditions. Adapted with permission from 93, copyrights 2022, Wiley-VCH.

Magnetic nanomaterials of various dimensions (0D, 1D, 2D) can be magnetically assembled and oriented in a pre-hydrogel solution under the influence of an external magnetic field. The 0D MNPs typically form a chain-like oriented structure when exposed to a magnetic field^19^, ^76^-^80^. Recently, Xu et al. 19 successfully produced highly oriented nanocomposite hydrogels that consist of type I collagen and oriented MNPs **(Figure 3A)**. Compared to pristine type I collagen and isotropic hydrogels, human Tendon Stem/Progenitor Cells (hTSPCs) had a higher value-added rate in highly oriented hydrogels, resulting in significant upregulation of tendon-related genes and good potential for clinical translation. Zhang et al. 80 also reported the self-assembly of SiO_2_-coated Fe_3_O_4_ nanoparticles into highly oriented unconfined filled arrays in a pre-hydrogel solution. The MNPs conferred magnetic and photothermal properties to the highly oriented hydrogels, resulting in self-healing properties, and their results showed that the viability of cells is not affected by the temperature changes induced by light and alternating magnetic fields.

Magnetic 1D nanorods or nanowires and 2D nanosheets may facilitate better orientation compared to magnetic spherical particles 81, 82. Several studies have reported the preparation of highly oriented hydrogels based on 1D nanomaterials utilizing magnetic fields 71, 81, 83-88. Rincón-Iglesias et al. 83 synthesized highly monodisperse ferrimagnetic Fe_3_O_4_ nanorods with adjustable size by solvothermal synthesis and varied the amount of hexadecylamine capping ligands. Subsequently, the nanorods were coated with gold shells and doped into agarose hydrogels, to obtain hydrogels with anisotropic magnetic and optical properties. These hydrogels have applications in fields such as regenerative medicine, cancer ablation, local therapy, and sensors due to their magneto-thermal and photothermal properties. Shi et al. 81 dispersed silica nanorods coated with Fe_3_O_4_ in a sol-gel state type I collagen and aligned the nanorods along the magnetic field with a weak magnetic field (<100 mT) generated by two plate-like magnets **(Figure 3B)**. Next, the sol-gel state was transformed into the gel state at 37°C to prepare a highly oriented hydrogel that facilitates the directional growth of normal human dermal fibroblasts.

Similarly, recent studies have shown that 2D magnetic nanosheets can create highly oriented structures when subjected to an external magnetic field 89-94. Dai et al. 91 accomplished the formation of two-dimensional magnetic nanosheet structures (MDS) by interleaving γ-Fe_2_O_3_ nanoparticles between two silicate layers. They then rotated the magnetic field (± 260 mT) to obtain a range of highly oriented poly(N-isopropylacrylamide) (PNIPAm) nanocomposite hydrogels. These MDS structures exhibit controlled anisotropy in optical and mechanical properties due to their sandwich structure, high aspect ratio, high charge density, and responsiveness to light and magnetic fields. Additionally, they exhibit anisotropic responses to temperature or light irradiation. Chen et al. 93 developed a novel programmable hydrogel that is magneto-thermally responsive and highly oriented **(Figure 3C)**. They anchored thermally sensitive PNIPAm and Fe_3_O_4_ magnetic nanoparticles onto the surface of MoS_2_ nanosheets and then embedded them in 3D-printed hydrogel cubes. They controlled the orientation of the 2D nanosheets by applying a magnetic field. In a separate study, Yan et al. 92 prepared a highly oriented hydrogel using catechol-mediated precipitation of Fe_3_O_4_ nanoparticles encapsulated on the surface of dialdehyde cellulose-polydopamine (DAC-PDA) nanomaterials, cross-linked with PAM chains under a magnetic field. The hydrogel possessed high electrical conductivity, strong adhesion, and biocompatibility, making it a promising candidate for various advanced applications, especially for flexible wearable sensors. The above examples demonstrate the wide range of current applications of magnetic materials in the preparation of highly oriented hydrogels and their potential for future use in TE.

Non-magnetic materials exhibit an extremely weak response to external magnetic stimuli and can be categorized as either antimagnetic or paramagnetic 42, 95. Several non-magnetic materials, including peptides 96, 97, protein fibers such as fibrin and collagen 98-101, cellulose nanocrystals 31, 102, 103, carbon nanotubes 88, 104, graphene 105, 106, and rare earth elements 107, have been explored for the preparation of highly oriented structural hydrogels. For instance, Radvar et al. 96 designed peptides that contained phenylalanine residues at the C-terminus and fabricated hydrogels with highly oriented nanofiber alignment through the self-assembly of peptides with hyaluronic acid. By exploiting the antimagnetic properties of phenylalanine residues under magnetic fields (12T, 6T, 1T), they achieved highly oriented hydrogels. Echave et al. 31 incorporated cellulose nanocrystals (CNC) into a gelatin network and utilized the antimagnetic properties of CNC to create highly oriented hydrogels that mimicked the alignment of tendon tissues under a magnetic field. The preparation of highly oriented hydrogels using antimagnetic materials is promising but challenging; most non-magnetic materials require high-intensity external magnetic fields, which can be unsafe for clinical TE, limiting their practical use.

##### 2.1.1.2. External Magnetic Field

For TE, magnetic fields can be categorized as static or alternating, depending on whether they are constant or continuously varying in amplitude and direction. Typically, static magnetic fields, generated with two parallel permanent magnets for a more uniform effect than single magnets, are used to generate the orientation of magnetic hydrogels. The size, composition, and purity of the magnets can be varied to control the magnitude of the static field 108. To date, several studies have employed static magnetic fields to orient MNPs in hydrogels along the magnetic field lines 76-80. Alternating magnetic fields can also be used to prepare highly oriented hydrogels 77, 109. Hu et al. 77 demonstrated this using a high-frequency (400 kHz) alternating magnetic field to orient MNPs into chains in a hydrogel matrix, enhancing its magneto-thermal properties. This technique allows control over thermogenesis by adjusting the angle between the hydrogel's structure and the magnetic field, making it suitable for controlled drug release.

**Figure 4:**
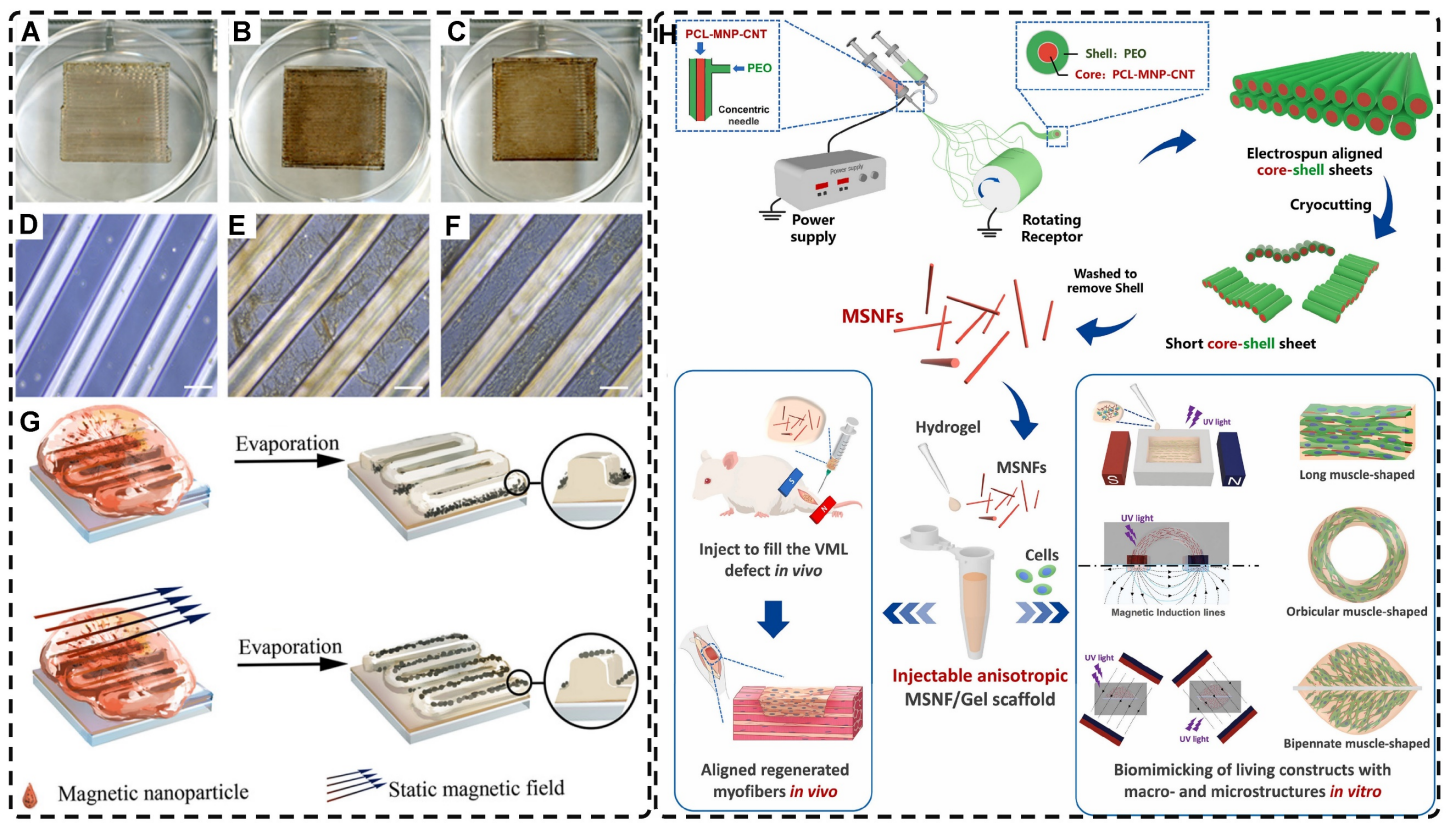
A-F) Macroscopic and microscopic images of MNP-, MNP+MNA-, and MNP+MNA+. G) Schematic diagram of hydrogels with oriented structures by combining 3D printing with MNPs induced by magnetic fields. Adapted with permission from 110, copyright 2021, Nature Publishing Group. H) Schematic illustration of injectable oriented MSNF/Gel nanofiber hydrogel scaffold for biomimicking of living constructs with macro- and micro-structures in vitro and aligned regenerated myofibers in vivo. Adapted with permission from 29, copyright 2022, Elsevier.

In addition, a magnetic field can guide the orientation arrangement of MNPs directly to assist in 3D printing or electrostatic spinning techniques. Hu et al. 110 also created hydrogels with oriented structures at both hundred microns and ~10 microns by combining 3D printing with MNPs induced by magnetic fields. These MNPs were sized similarly to adipose tissue-derived stem cells (ADSCs), allowing ADSCs to better sense the orientation structure and promote their differentiation towards osteogenesis** (Figure 4A-G)**. Johnson et al. 51 used oleic acid-coated superparamagnetic iron oxide nanoparticles doped into poly-l-lactic acid (PLLA) to prepare small conduits of aligned magnetic fibers through electrospinning. These small conduits were combined with collagen and fibrinogen hydrogel solutions, and then prepared under magnetic fields to form a highly oriented hydrogel that directed the growth of primary dorsal root ganglion. Wang et al. 29 prepared monodisperse magnetic controlled short nanofibers (MSNFs) using an advanced coaxial electrospinning-cyrocutting method and further designed an injectable oriented MSNF/Gel nanofiber hydrogel scaffold **(Figure 4H)** that guided the 3D alignment and mimicry of cells with macroscopic and microscopic biological structures in vitro, demonstrating great potential in promoting skeletal muscle regeneration in vivo.

In fact, designing oriented hydrogels by orienting magnetic materials in hydrogel matrices with low-intensity magnetic fields has been a preferred method 42, 111. This approach enhances biocompatibility, orientation, and magnetic responsiveness while also providing unique advantages in remote control, bionanotechnology, and mechanical properties. Randomly oriented MNPs can transit from superparamagnetic to weakly ferromagnetic; the alignment of MNPs changes their magnetic properties, explained by dipolar moments, creating local magnetic order that persists even without an external magnetic field 112. This enables MNPs to generate a micro-magnetic driving forces between the hydrogel scaffold and the cells, activating cell surface-sensitive receptors, ion channels and triggering mechanotransduction pathways for remote cell stimulation for remote cell stimulation to influence cell growth 59, 112-114. Several studies have shown that the bionic oriented structure of magnetic highly oriented hydrogels enhances cellular localization, proliferation, and differentiation, making them an excellent candidate for TE e.g., tendon regeneration, skin regeneration, and nerve regeneration 19, 29, 76, 81, 84, 85, 115, 116.

The biosafety and fate of biomimetic hydrogels in living organisms are critical for TE. Iron oxide-based MNPs, commonly used in MRI, drug delivery, thermal therapy etc., are recognized for their biosafety and eventual metabolism by the host immune system following in vivo administration 117-121. However, MNPs' effects and pharmacokinetics vary based on size, shape, chemical composition, and surface coating. Magnetic hydrogels interact differently with cells or tissues depending on their intended use, and comprehensive data are necessary to ensure their controlled, stable, safe, and effective use in clinical TE 122, 123. Future TE studies should consider as many of these factors as possible to demonstrate the potential and promising future of highly oriented hydrogels induced by magnetic fields in conjunction with other ordered manufacturing methods.

#### 2.1.2. Electric-Field-Induced Highly Oriented Nanofiller Hydrogels

Applying an external electric field induces the distributed polarization of free electrons on particles, generating an electric dipole moment. This phenomenon enables the electric field to manipulate the alignment and assembly of materials in solution that have permanent or induced dipole moments, in addition to the magnetic field. The migration of dispersed particles in a fluid under a uniform electric field is referred to as electrophoresis (EP), while the migration of particles in a non-uniform electric field is referred to as dielectrophoresis (DEP) 124-126. Using different pre-gel media and electro-responsive building blocks of varying sizes, oriented structures with diverse orientations can be obtained by driving them with EP or DEP under different external electric fields. These oriented structures are then immobilized in the hydrogel matrix via a sol-to-gel state transition.

**Figure 5:**
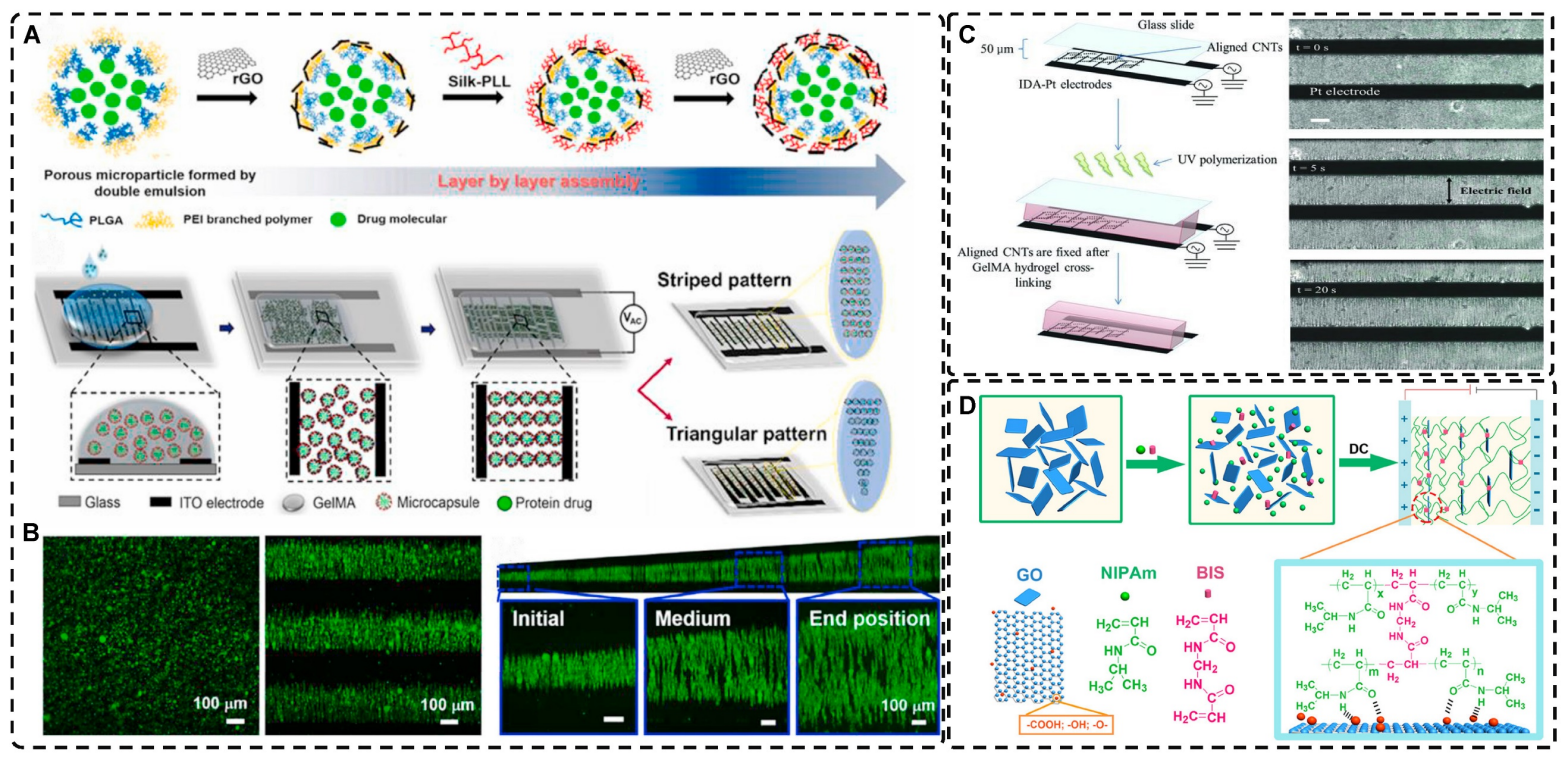
A) a) Synthesis of 0D microcapsule assembly. b) The fabrication process of orienting 0D microcapsule alignment by DEP manipulation. B) Random distribution of 0D microcapsule and subsequent orientation alignment within the hydrogel after DEP. Adapted with permission from 128, copyright 2021, Elsevier. C) Schematic representation of the fabrication process for CNT alignment within the GelMA hydrogel and phase contrast images of the CNT alignment over time. CNTs were aligned after 20 seconds. Adapted with permission from 129, copyright 2013, Wiley-VCH. D) Scheme of preparation of 2D PNIPAm/GO oriented hydrogel. Adapted with permission from 131, copyright 2018, American Chemical Society.

Electric fields, like magnetic fields, can induce materials of different morphologies to assemble into oriented structures in hydrogels. Morales et al. 127 and Zhang et al. 20 aligned 0D polystyrene particles into chains of different orientations using DEP forces under an alternating current (AC) electric field, creating endoskeleton-like particle chains, resulting in composite hydrogels with unique mechanical, optical, and anisotropic properties compared to isotropic hydrogels. Potential applications of these hydrogels include the development of soft robots and smart materials. Recently, Chiang et al. 128 designed graphene oxide/filament-poly-L-lysine layer-assembled PLGA 0D porous microcapsules (PM) loaded with NT-3 **(Figure 5A)**, arranged in linear or triangular highly oriented structures within photocrosslinked gelatin methacrylic acid (GelMA) hydrogels by DEP. These triangular hydrogels can form a gradient distribution of NT-3 that could significantly guide the migration of NSCs and enhance spinal cord injury repair. Such spatially highly oriented 4D hydrogels have great potential to replace 3D hydrogels in bionic TE for better mimicking the pericellular microenvironment.

Carbon nanotubes, commonly used as 1D filler materials in hydrogel substrates, can be assembled into oriented composite hydrogels under the influence of an external electric field 129, 130. Ahadian et al. 129 utilized DEP to align carbon nanotubes (CNTs) within GelMA hydrogels under a 2 MHz AC electric field at 20 V, resulting in oriented hydrogels that achieved superior maturation of muscle myofibers compared to hydrogels with randomly distributed CNTs **(Figure 5B)**. Moreover, their oriented GelMA-CNT hydrogels, prepared under a 1 MHz AC electric field at 20 V, promoted the differentiation of mouse embryoid bodies (EBs) to cardiomyocytes more efficiently than isotropic hydrogels 130. Notably, cells on the oriented hydrogels exhibited enhanced migration and differentiation after applying electrical stimulation in the direction of orientation, which is attributed to the excellent electrical conductivity.

Electric fields have also been used to orient 2D graphene nanosheets as hydrogel filler materials 131, 132. Yang et al. 131 achieved an anodically oriented arrangement of negatively charged 2D graphene oxide (GO) through EP under a DC electric field, which resulted in a bionic gradient-oriented structure after cross-linking gelation in poly(N-isopropylacrylamide) hydrogels** (Figure 5C)**. Its near-infrared light response and programmable motion make it a promising candidate for artificial muscles and soft brakes. MXenes are a new class of 2D materials that consist of transition metal carbides, nitrides or carbonitrides with the general formula of M *_n_*_+1_X*_n_* (*n* = 1-3), where M represents an early transition metal (such as Sc, Ti, Zr, Hf, V, Nb, Ta, Cr, Mo) and X is either carbon or nitrogen 133-135. Using the electric field-assisted forced-assembly technique, Dutta et al. 136 were able to obtain hydrogels with aligned Ti_3_C_2_T_x_ Mxene nanosheets. The gelation process depended on the type of metal electrode and redox potential, and adjusting the electric field distribution allowed for various sheet-like alignments, useful for capacitors and hot water evaporators. MXene's oriented structure also promoted cell adhesion, proliferation, and guided growth, highlighting its potential in biomedicine TE 137. Zhu et al. 138 induced highly charged nanosheets (Hectorite [Na_0.5_]^inter^[Li_0.5_Mg_2.5_]^oct^[Si_4_]^teter^O_10_F_2_) to form complex oriented structures in a pre-hydrogel matrix using patterned electrodes etched on glass substrates. Subsequently, these structures were immobilized by UV photopolymerization to form highly oriented bionic hydrogels that can deform in a directed way under external stimuli, which is compelling for applications in artificial muscles and soft brakes. Furthermore, the controllable patterned distribution of electric fields could be applied to orienting other charged particles through electrophoresis.

Several studies have shown that external electric fields can be utilized for straightforward, cost-effective, and flexible assembly of materials into bionic hydrogels with oriented structures, with great potential in TE. They are favored by researchers to regulate the growth, orientation, migration, and differentiation of various cell phenotypes in vitro and in vivo, given the involvement of endogenous electric fields in the growth and repair of cells, tissues, and organs 139. Future research can explore the potential of electric field induction to create oriented structural hydrogels and direct the alignment of cell growth within these hydrogels for TE.

However, it should not be ignored that electric fields are more reliant on the electrochemical stability of the hydrogel substrate material when filling the oriented hydrogel with nanomaterials or its own polymer network, compared with magnetic fields. Moreover, direct contact with the precursor solution during gel synthesis may result in unforeseen electrochemical reactions and/or electrophoresis. Consequently, optimization of voltage and current based on the electrochemical stability of various materials is necessary. These limitations greatly restrict the use of electric field-induced oriented hydrogels.

#### 2.1.3. Mechanical Force-Induced Highly Oriented Nanofiller Hydrogels

Mechanical force-induced alignment involves arranging disordered polymer chains or filler materials in hydrogels in response to external stress, like stretching or shearing. The mechanical force or strain can be applied during or after partial or complete gelation, resulting in the alignment of the nanofiller material and/or hydrogel polymer network in the direction of the applied strain. Compared to other methods, this technique is simple, fast, and straightforward, making it widely used in the preparation of highly oriented nanocomposite hydrogels.

**Figure 6:**
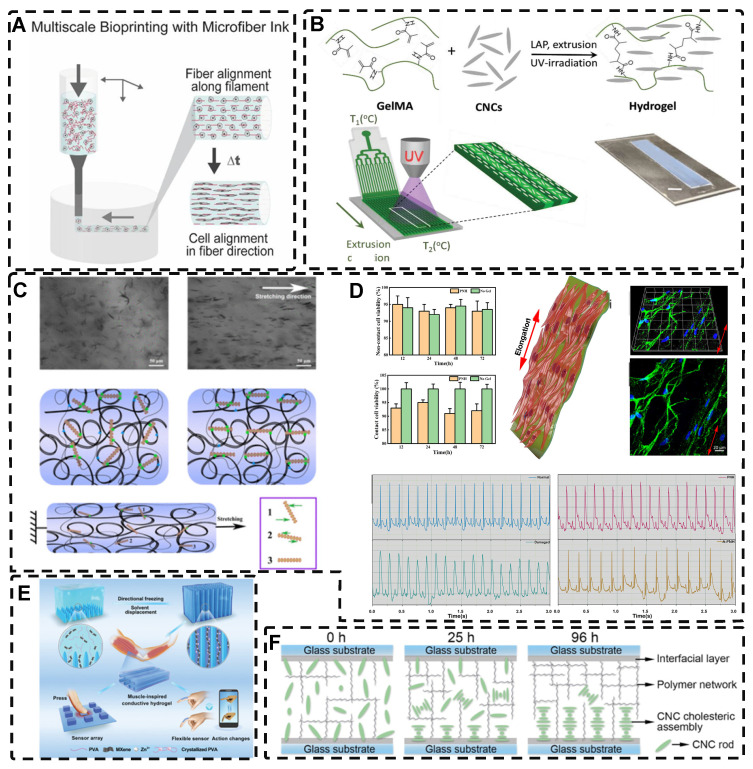
A) Schematic of prepared highly oriented, fiber-aligned hydrogels using shear forces during extrusion printing. Adapted with permission from 152, copyright 2021, IOP Publishing. B) (a,b)Schematic of shear-mediated extrusion of highly oriented hydrogel from the mixture of CNCs, GelMA, and LAP using a microfluidic printhead, and (c) photograph of the hydrogel sheet. Adapted with permission from 143, copyright 2021, Wiley-VCH. C) Confocal microscopy images of random aligned hydrogels and highly oriented hydrogels, and schematic of preparation of highly oriented PPy nanocomposite hydrogels after repeated stretching. D) They have good biocompatibility, induce targeted growth of cardiomyocytes and promote restoration of electrical conductivity. Adapted with permission from 153, copyright 2021, American Chemical Society. E) Schematic representation of the synthesis procedures of oriented void channels hydrogels with an ordered internal orientation structure and further applications in wearable flexible sensors and 3D sensor arrays. Adapted with permission from 162, copyright 2021, Wiley-VCH. F) Schematic of self-assembled oriented structures of CNC arrangement in the polymer network after UV curing with different standing times Adapted with permission from 168, copyright 2021, Elsevier.

Among the mechanical induction methods, the preferred method for preparing highly oriented nanofiller hydrogels is using shear forces 21, 140-151, which induce larger and more well-ordered alignments compared to magnetic and electric fields, and cover larger areas. However, as with magnetic and electric fields, the metastable state of the induced highly oriented alignment structure may be changed or destroyed when the external field is removed or altered. To prevent loss of the oriented structure, the hydrogel can act as an external matrix to fix the highly oriented structure during the transition from the sol to the gel state. In recent years, 3D printing has led to tremendous growth in the application of bionic hydrogel scaffolds in TE. Shear force-induced directional alignment of polymer chains and/or filler materials within hydrogels is widely used during the printing process when the bioink passes through the nozzle and generates shear forces. Zhao et al. 21 utilized shear force to align and distribute CNTs within hydroxyethyl methacrylate (HEMA) gels, resulting in composite hydrogels with varying CNTs orientations that exhibited distinct mechanical properties. Kumacheva et al. 141-143 published a series of studies on the preparation of highly oriented hydrogels by shear induction **(Figure 6B)**. They recently used a 3D printed microfluidic print head to extrude a hydrogel consisting of CNCs and GelMA through 16 microchannels with varying shear forces 143. Due to the different shear forces, the CNCs were oriented differently and subsequently photo-crosslinked to lock the oriented CNCs within the hydrogel matrix. This uniaxially oriented hydrogel could be applied in TE and regenerative medicine of e.g., artificial muscles.

In addition to orienting filler material, 3D printing can also align cells within hydrogels using shear force has become a popular topic in hydrogel development. When hydrogels contain both filler and cells, this aligned structure allows cells to grow along it, more effectively replicating the anisotropic nature of human tissues. Prendergast et al. 152 created fiber-aligned hydrogels by mixing hydrogel fiber suspensions, derived from mechanically fragmented electrospun scaffolds of norbornene-modified hyaluronic acid (NorHA), with GelMA. They used shear forces during extrusion printing to achieve high orientation **(Figure 6A)**. After optimizing the suspension properties and printing flow rate, meniscal fibroblasts, mesenchymal stromal cells, or cardiac fibroblasts immobilized in composite-oriented hydrogels were induced to align by the highly oriented fibers, in contrast to random cell growth in non-oriented hydrogels. This provided the possibility to construct meniscus, cartilage, heart, and other tissues in vitro for TE repair and the development of physiological tissue models.

Stretch induction, the other common method for orienting hydrogel polymer chains, can also induce the alignment of filler materials within the hydrogel. Wang et al. 153 elastically stretched a composite hydrogel of sodium alginate, acrylamide, and polypyrrole (PPy), and found that PPy nanotubes tended to align parallel to the stretching direction and slide relative to the hydrogel network, resulting in the successful preparation of highly oriented PPy nanocomposite hydrogels after repeated stretching **(Figure 6C)**. The highly oriented hydrogel induced by stretching can promote the formation of an elongated cellular morphology in cardiomyocytes, and its highly biocompatible, electrically conductive, and stretchable properties make it a promising candidate for cardiac TE **(Figure 6D)**. Hu et al. 154 developed stretchable supramolecular hydrogels through host-guest interactions between β-cyclodextrin-modified tunicate cellulose nanocrystals (TCNCs) and damantane-containing polymers. Pre-stretching of the hydrogels caused the polymer chains and TCNCs in the network to move and align in the direction of the stretching force, and the highly oriented TCNC structures were then locked in the hydrogel network through the ionic coordination between Fe^3+^ and -COO on acrylic acid, by immersion in a FeCl_3_ solution. Additionally, the group utilized TCNC fillers to pre-stretch the oriented structure within a semi-rigid sodium alginate network and subsequently locked it via Ca^2+^/-COO ion coordination. These hydrogels exhibited superior mechanical properties compared to isotropic hydrogels due to their highly oriented filler structures, making them promising candidates for TE applications such as ligaments and tendons.

The preparation of highly oriented nanofiller hydrogels by mechanical induction is a simpler and more feasible method compared to other techniques. Mechanical tensile induction is more commonly used to achieve the highly oriented orientation of hydrogel polymer chains. While the development of 3D printing has led to an increased application of shear force induction in preparing highly oriented hydrogels, producing thicker highly oriented filled structure hydrogels is challenging due to the uneven distribution of shear force 37. In addition, the orientation of the filler material by shear force is influenced by various factors, such as the rheological properties of the ink, the properties of the filler material, and the specifications of the nozzle. If the ink viscosity is low, the orientation structure may be lost before the hydrogel gels, whereas high viscosity can result in higher flow resistance leading to clogging of the extruder and inconsistent ink deposition 123. Moreover, when hydrogels are used with cells, the impact of shear force on the cells should be considered, as excessive shear force can reduce the activity and function of the cells and even cause apoptosis 155. Therefore, in the field of TE, optimizing the relationship between shear force, nanomaterial orientation, hydrogel rheology, and viability of the loaded cells is a major challenge that needs to be addressed in producing highly oriented biomimetic hydrogels.

#### 2.1.4. Other Strategies for Preparing Highly Oriented Nanofillers Hydrogels

In addition to using magnetic fields, electric fields, and mechanical forces, methods like directed freezing and self-assembly are also utilized to prepare highly oriented nanofiller hydrogels.

Directed freezing is a common method used to prepare directed macroporous hydrogels, as will be further discussed in Section 2.3. During the formation of ice crystals caused by a temperature gradient, the hydrogel network and/or filler material can be separated and confined to the interstitial region between the ice crystals, which allows the filler material to be oriented in the hydrogel while preparing the macroporous structure 156-161. Drawing inspiration from muscles, Feng et al. 162 developed oriented void channels hydrogels with an ordered internal orientation structure, by repeatedly freezing MXene nanosheets processed with Ti_3_AlC_2_, mixed with polyvinyl alcohol (PVA), and ZnSO_4_ in Polytetrafluoroethylene (PTFE) molds. The MXene nanosheets were connected to the PVA main chain through hydrogen bonding, and the addition of Zn^2+^ ions disrupted the electrostatic repulsion between the MXene sheets, generating high binding energy with the -OH groups on the MXene surface, forming an ion-chelated cross-linked network that was crucial in improving the mechanical properties of the hydrogels. During the freezing process, ice crystals created occupancy, while PVA chain segments and MXene nanosheets were squeezed at the water-ice interface to form a highly ordered arrangement in the hydrogel. This reduced the distance between nanosheets, thus enhancing the electrical conductivity of the hydrogel. Moreover, the formation of small microcrystals after repeated freeze-thawing of PVA strengthened the network structure of the hydrogel, resulting in excellent shape recovery properties. These outstanding properties make this muscle-inspired, highly oriented macroporous hydrogel a promising material for sensor applications **(Figure 6E)**.

Self-assembly is a widespread phenomenon that plays a crucial role in biological growth and development. Examples include the hybridization of the DNA double helix, protein folding, and the self-assembly of troprocollagen into highly ordered and tightly arranged collagen fibers in tissues like tendons 163, 164. Drawing inspiration from these natural processes, self-assembly has become prominent in TE. This method, where the basic structural units within a hydrogel spontaneously assemble into an ordered structure through non-covalent bonds, such as hydrogen bonds, van der Waals forces, and hydrophobic forces 165, 166, has been researched for the construction of tissues and organs. The lack of need for external forces enhances cell-material interactions in TE, expanding the potential applications of self-assembly methods 167. Liu et al. 168 embedded self-assembled oriented structures of CNCs into polyethylene glycol derivatives/polyacrylamide hydrogel precursors. The arrangement of CNCs in suspension or pre-polymerization solution was influenced by time and self-assembly angle **(Figure 6F)**; over time, the CNC rods sank due to gravity, and as the self-assembly angle increased from 0° to 90°, the arrangement of CNC rods transitioned from a chiral nematic structure to a symmetrical nematic structure. These hydrogels thus exhibited great potential as mechanical stress and temperature multi-stimulation sensors. Furthermore, the high transparency of hydrogels in visible light makes them suitable for anti-counterfeiting applications.

Self-assembly is a highly organized process at the molecular level, but achieving highly oriented spatial structures in hydrogels solely by using self-assembling filler material can be challenging. External forces are often needed to control the mesoscopic order of self-assembly and create highly ordered hydrogel-filled structures 169. For instance, Akhil Patel et al. 170 self-assembled graphene-doped chitosan (CHT) with gellan gum (GG) in a microfluidic chamber and aligned them with the aid of long needles (shear force) to form self-assembled hydrogels with a unidirectional fiber structure. These hydrogels were then manually collected to form films with graphene sheets evenly dispersed within them. The inclusion of graphene enhanced the mechanical strength and electrical conductivity of the hydrogels, promoted cell adhesion, and induced the initial orientation of myogenic cells, leading to the formation of multinucleated myotubes. As a result, these hydrogels may have potential applications in skeletal muscle TE.

In addition to shear, magnetism-induced and directional ultrasound can also be applied in conjunction with self-assembly 171-175. Despite the significant progress made in self-assembly strategies in recent decades, the preparation of highly oriented hydrogels through self-assembly still poses challenges, such as the development of simple and feasible strategies, and the need for more precise control of self-assembled modules.

In general, although most of the nanofillers materials themselves have excellent biocompatibility and thus influence the hydrogel performance, such as MNPs, CNTs, MXene and other materials, they can enhance the biocompatibility, mechanical properties and other characteristics of the composite hydrogel in different degrees and fulfill the function of the material itself. More importantly, when endowed with a highly oriented structure, the hydrogel nanofiller material more closely resembles the bionic structure of highly oriented tissues, thereby regulating tissue repair and regeneration. Although the study of how highly oriented hydrogels affect the mechanism of cell behavior is not clear so far, highly oriented nanofillers hydrogels occupy an important position in the field of TE.

### 2.2. Highly Oriented Polymer-Chain Network Hydrogels

In addition to manipulating the orientation of nanofiller materials, utilizing polymer-chain networks is another strategy for preparing highly oriented hydrogels. Highly oriented polymer-chain network hydrogel is a type of hydrogels which the polymer-chain networks of hydrogel monomers are oriented. And thevmost important preparation methods for highly oriented polymer-chains hydrogel is mechanical stretching.

#### 2.2.1. Mechanical Stretch-Induced Highly Oriented Polymer-Chain Network Hydrogels

As previously discussed in the section on mechanical force-induced orientation of filled materials, mechanical stretching is also a widely used method for inducing the orientation of hydrogel polymer chains. This method can easily orient a network of randomly oriented hydrogel polymer chains along the stretching direction. However, due to the elastic nature of hydrogel polymer networks, it is difficult to maintain the structure as they tend to revert to their original, less ordered isotropic state once external forces are removed. For example, dual network hydrogels made of calcium alginate (ALg) and polyacrylamide (PAM), crosslinked ionically and covalently, are highly stretchable 2 . When stretched, the ionically cross-linked ALg chains in the stretched state become unlinked, break and align in the direction of the stretching force. However, the stable deformability of PAM chains makes the double network hydrogels reversible. To maintain the structure and maximize orientation retention after stretching, a strategy has been developed. Kim et al. 176 achieved an irreversible fixation state of the highly oriented structure by crosslinking alginate chains after directional stretching. This secondary ionic crosslinking largely retained the orientation after the stretching force was removed and increased the elastic modulus of the hydrogel. Additionally, by embedding mesoporous silica microrods as inorganic fillers in the double network hydrogels 177 they improved the mechanical properties of the hydrogels, creating structures that resemble ligaments or tendons.

**Figure 7:**
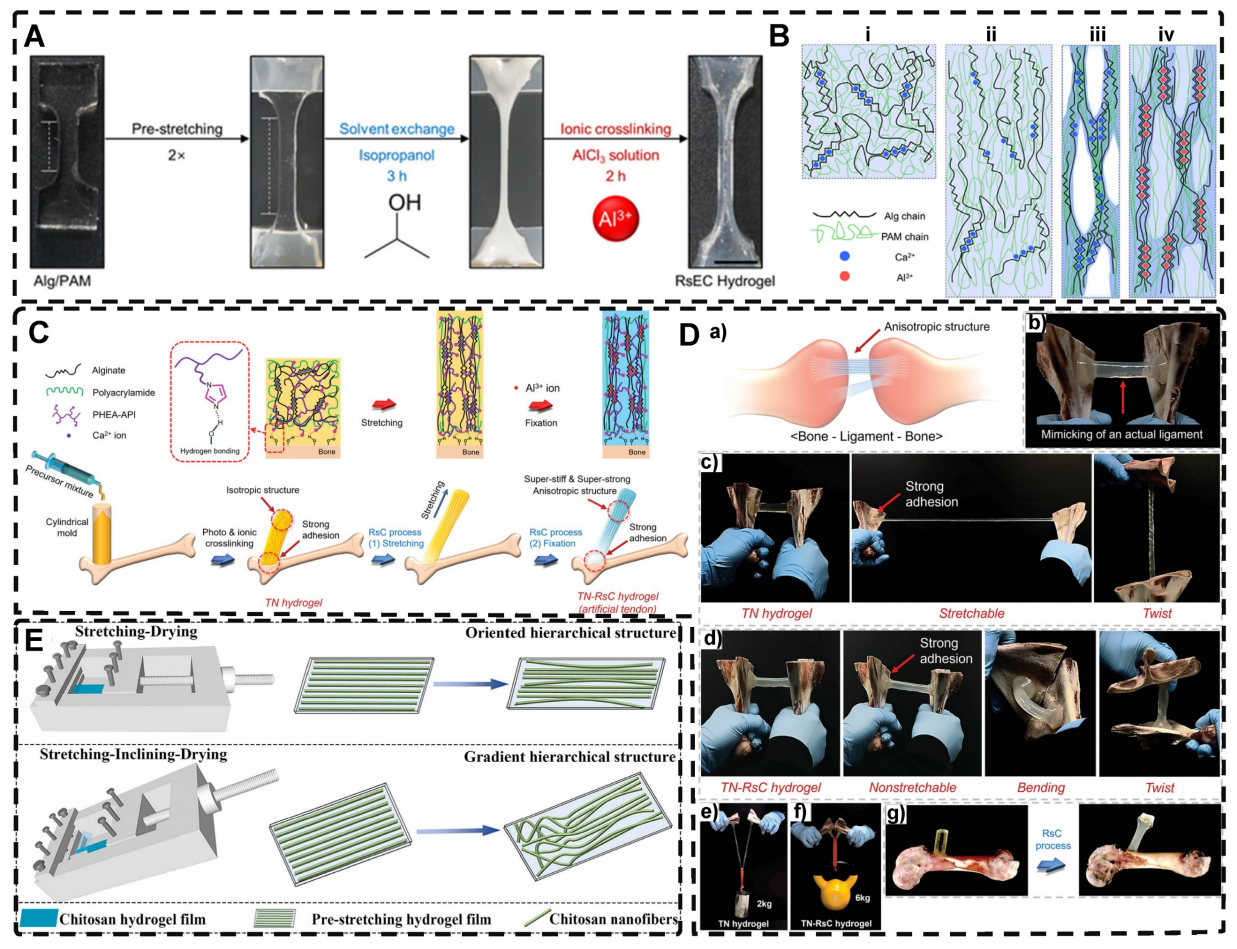
A) Photographs of the highly oriented Alg/PAM hydrogels during the RsEC process; remodeling (pre-stretching) and subsequent solvent exchange followed by ionic crosslinking (RsEC). B) Illustrations of the changes in the molecular structure during the RsEC process: (i) as-prepared Ca^2+^-crosslinked Alg/PAM hydrogel, (ii) pre-stretched hydrogel, (iii) solvent-exchanged hydrogel, and (iv) Al^3+^ crosslinked hydrogel. Adapted with permission from 179, copyright 2022, American Chemical Society. C) Design of the strong, stiff adhesive highly oriented TN hydrogel. The final TN-RsC highly oriented hydrogel subjected to the RsC process was cross-linked by Al^3+^ ions and exhibited strong mechanical and adhesion properties. D) a) Schematic of the real ligament and anisotropic structure of the ligament, and b) photographs of TN-RsC hydrogel mimic the real ligament. Adapted with permission from 180, copyright 2022, Wiley-VCH. E) Graphical illustrations for the production of oriented and gradient chitosan hydrogel films with hierarchical structure via the self-made mold. Adapted with permission from 181, copyright 2022, Elsevier.

Chen et al. 178 also prepared highly oriented ALg/PAM hydrogels using stretching and subsequent ionic cross-linking strategies. Their hydrogel, which was significantly oriented in both microstructure and ion transport, provides a promising direction for the development of next-generation wearable electronics. In another study, Park et al. 179 replaced Ca^2+^ with Al^3+^ as the cross-linking agent **(Figure 7A-B)**. By adjusting the degree of directional stretching, the mechanical properties of the hydrogel could be controlled within a certain range. The layered combination of oriented hydrogel cables prepared by applying a braiding textile process exhibited a significant load-bearing capacity and tensile behavior similar to that of tendons. Additionally, Choi et al. 180 developed highly oriented triple network hydrogels by combining poly (2-hydroxyethyl aspartamide) modified with aminopropyl imidazole (PHEA-API), which provided strong bone adhesion through multiple hydrogen bonds, and an energy-dissipative Alg/PAM double-network **(Figure 7C-D)**. The triple network hydrogels had high tensile modulus and strength, mimicking natural ligaments, and exhibited high adhesion properties at the interface with bone. This work set the foundation for future hydrogels with high mechanical properties and adhesion to bone.

Thus, the preservation of the highly oriented structure after directional stretching can be achieved by controlling the supramolecular interactions between polymer chains. Mredha et al. 22 stretched rigid/semi-rigid alginate/cellulose chains to obtain a highly oriented single polymer network hydrogel. Subsequently, the supramolecular interactions between the highly oriented polymer chains, such as hydrogen bonds/ionic bonds, were strengthened by a specific drying method, thereby strengthening and maintaining the structure and preventing it from being destroyed upon rehydration. Li et al. 181 achieved highly oriented chitosan hydrogels using the same method, and the good biocompatibility of the hydrogel and the highly oriented structure induced the proliferation and orientation of osteoblasts and fibroblasts **(Figure 7E)**. For flexible polymer chains, Chen et al. 182 prepared highly oriented PVA hydrogels using stretch-freezing cycles. Stretching oriented the PVA chains and brought the isotropic and loose PVA chains relatively close together, while freezing further brought the chains into close contact. The PVA chains in close contact easily adopted suitable conformations for hydrogen bond formation, and therefore more hydrogen bonds were formed between the oriented PVA chains, and even the cooperativity of hydrogen bonding became possible when consecutive hydrogen bonds were formed. As a result, the hydrogels maintained high orientation even after thawing, significantly enhancing their mechanical properties. Wang et al. 183 prepared PVA/NaCl highly oriented hydrogels using a hot stretch freezing method. These hydrogels exhibited various anisotropic ion transport behaviors, providing a new strategy for developing human sensors. Sun et al. 184 utilized the abundant hydrogen bonding between stiff aramid nanofibers (ANFs) and flexible PVA to achieve permanent alignment of the fiber network, resulting in hydrogels with robust mechanical properties, which was confirmed by SEM analysis. The excellent mechanics and functionality of these hydrogels, which mimic tendons, suggested their potential for use in advanced TE. Although the mechanical stretching method is simple and effective, its requirement for a large stretching force for the precursor hydrogel polymer network imposes some limitations.

#### 2.2.2. Other Strategies for Preparing Highly Oriented Polymer-Chain Network Hydrogels

The polymer network of hydrogels can also be directed by ion diffusion, shear induction, and electric field induction.

**Figure 8:**
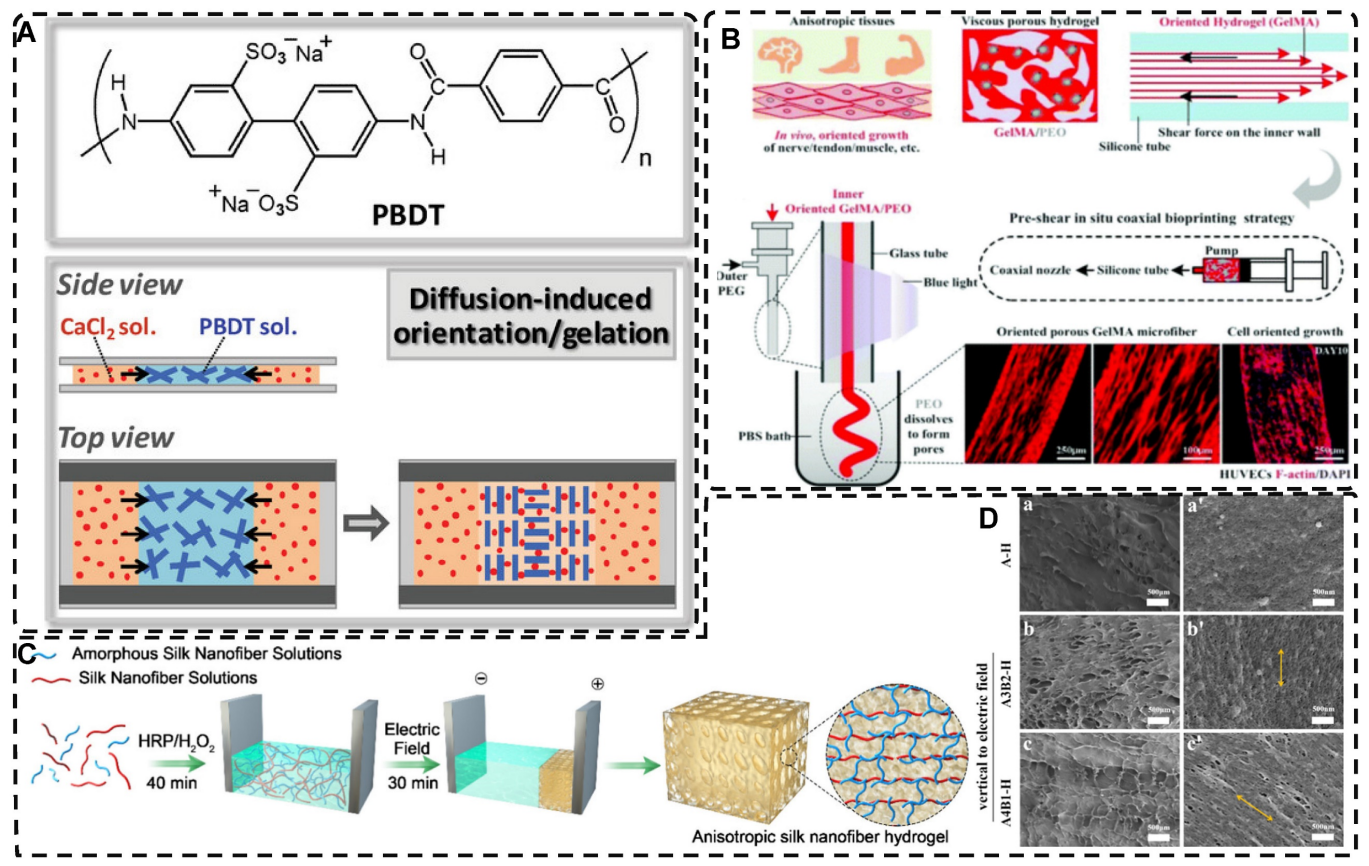
A) Chemical structure of the rigid polyanion, PBDT, and schematic for the formation of anisotropic hydrogel by unidirectional diffusion of Ca^2+^ ions into PBDT solution from two opposite lateral sides. The diffusion induced the alignment of PBDT perpendicular to the diffusion direction near the entrance yet parallel to the diffusion direction at the middle region where the two fluxes met. Adapted with permission from 185, copyright 2019, WILEY‐VCH. B) Schematic illustration and effect of pre-shear bioprinting of highly oriented hydrogel microfiber-enabled oriented growth of cells. Adapted with permission from 191, copyright 2021, Royal Society of Chemistry. C) Process to Form Tough Silk Nanofiber Hydrogels with highly oriented Architectures. D) Characterization of SF hydrogels with highly oriented structures. Adapted with permission from 195, copyright 2020, American Chemical Society.

In directional ion diffusion, a cationic aqueous solution is brought into contact with an anionic polymer aqueous solution to drive directional cation diffusion. As diffusion proceeds, the anionic polymer chains are cross-linked, forming a hydrogel with a network of directional polymer chains 23, 185-187. Yang et al. 23 physically formed poly(2,2'-disulfonyl-4,4'-benzidine terephthalamide) (PBDT) gels with a highly ordered structure by uniaxially controlling the diffusion of Ca^2+^ as a cross-linking agent in the solution of PBDT in the reaction mold. The highly oriented PBDT gels were then immersed in an acrylamide (AAM) solution containing the chemical cross-linker to chemically form the PAAm gel as a second network, resulting in highly oriented double network hydrogels with excellent mechanical properties. Qiao et al. 185 designed masks with holes to guide the diffusion of multivalent metal ions into the bottom solution of anionic PBDT, allowing for the preparation of hydrogels with complex ordered structures **(Figure 8A)**. By programming the diffusion field and adjusting the pattern and position of the mask containing holes, such highly oriented hydrogels with high mechano-optical sensitivity have potential for use in e.g., optical devices and sensors, and provide new ideas for preparing hydrogels without bionic complex tissues. In addition to polysaccharides and alginates, which have been used as anionic polymers in aqueous solutions, Ca^2+^, Zn^2+^, and Cu^2+^ have been used as the corresponding cations. Mredha et al. 186 formed an anisotropically oriented structured hydrogel on the surface of a core hydrogel by the controlled diffusion of cations in a pre-designed core hydrogel through a biopolymer storage solution, resulting in an oriented tubular alginate hydrogel with mechanical properties comparable to those of natural blood vessels. By controlling the concentration, diffusion time, and flow direction of ions and the size and shape of the core hydrogel, complex ordered tubular hydrogels with different 3D structures can be prepared, and even live cells can be carried. While this synthesis process is relatively simple, the relationship between the diffusion direction and polymer chain orientation has not been elucidated, and the resulting hydrogels had a non-homogeneous crosslink density gradient along the diffusion direction.

Shear forces can also be applied to orient a polymer chain network of a hydrogel. Nazhat et al. 188, 189 developed the gel aspiration-ejection (GAE) method, which uses shear stress to prepare injectable dense collagen (I-DC) hydrogels with highly oriented collagen fibers. The shear stress reshaped the geometry of the gel and aligned the collagen nanofibers along the long-axis direction. In addition to its ability to guide the orientation and alignment of inoculated cells, this highly oriented hydrogel mediated osteoblast differentiation of murine mesenchymal stem cells (MSCs) and supported and accelerated neuronal transdifferentiation. Additionally, a high strain mechanical stimulation of this highly aligned dense collagen fibril hydrogel induced tenogenic commitment of MSCs 190. Shao et al. 191 produced a highly oriented, porous, cell-laden GelMA hydrogel by mixing PEO, different types of cells, and GelMA **(Figure 8B)**. The oriented hydrogel chains were sheared through a coaxial nozzle, followed by photo-crosslinking, fixation, and washing off polyethylene glycol PEG in PBS. The cells encapsulated in this hydrogel showed highly oriented growth behavior similar to that in vivo, offering more biomimetic options for TE. Chen et al. 14 developed an alginate-assisted microfluidic system that can be fine-tuned with a combination of methacrylate hyaluronan (HA-MA) and fibrin (Fb) as well as microfluidic shear and stretch parameters to optimize the homogeneous orientation of bionic nerve fibers. The highly oriented hydrogel was guided by nano-topography in a focal adhesion kinase-associated manner, promoting aligned neurite growth, directional extension of axons, and myelin maturation of Schwann cells. This bionic neural composite hydrogel has significant potential in neural regeneration as well as other bionic TE applications. Kim et al. 192 utilized the shear stress of 3D printing nozzles to prepare highly oriented collagen hydrogels that contained various types of cells. The physical and biochemical cues provided by the collagen induced attachment, growth, and alignment of cardiomyocytes (H9C2), preosteoblasts (MC3T3-E1), and human adipose stem cells (hASCs) in a synergistic manner. Additionally, the shear force-induced cell alignment was achieved without losing cell viability.

Electric fields can also orient hydrogel polymer chains in addition to inducing the orientation of the filler material within the hydrogel, as described in the previous section 193. Tong et al. 194 developed highly oriented chitosan hydrogels with complex layering by exposing chitosan solutions to electrical signals of varying intensities; chitosan chains assembled and aligned at the cathode and reacted with OH- ions to create an ordered structure. By regulating the electrical signal, a direct equilibrium between OH- diffusion rate and chitosan electrophoretic rate was established, allowing for precise and continuous control of the internal structure of the hydrogel. This novel approach for multilayered, orderly assembly of polysaccharides facilitates the replication of complex structures. It serves as a valuable tool in TE for creating for example bionic, multi-layered blood vessel structures. Ding et al. 195 used horseradish peroxidase (HRP) cross-linked with amorphous silk nanofibers (ASNFs) in an electric field to form the native hydrogel, and at the same time, β-sheet-rich silk nanofibers (BSNFs) were aligned and oriented as reinforcing chains during cross-linking, resulting in a structured composite with enhanced mechanical properties **(Figure 8C-D)**. The highly oriented structure can provide various physical cues for cells, such as better stiffness and alignment aggregation, promoting stem cell proliferation and osteodifferentiation induction in vitro, with potential applications in bone TE. Moreover, Abu-Rub et al. 196 used an electric field to prepare highly oriented collagen fiber hydrogels, which supported directional orientation, growth, and guidance of nerve protrusions, overcoming the inhibitory aspect of myelin-associated glycoprotein on nerve protrusion growth. This work provides a reference for preparing nerve-guided catheters for spinal cord injury.

Compared to hydrogels filled with highly oriented materials, orienting the polymer chains is relatively straightforward, requires less equipment, and is more reproducible. Moreover, the oriented polymer chains within the hydrogel provide a continuous physical topographic cue to the cells, facilitating cell adhesion, migration, and growth, which is crucial for TE regeneration. Nonetheless, several preparation strategies have limitations. Mechanical stretching necessitates a specific polymer chain tolerance, ion orientation yields non-homogeneous hydrogels, and electric field induction may produce unexpected electrochemical reactions or electrophoresis of the polymer solution. Additionally, accurately replicating complex and intricate tissue structures while maintaining highly oriented structures and mechanical properties similar to human tissues remains challenging. However, as further investigations proceed, hydrogels with highly oriented polymer chains have a bright future in TE.

### 2.3. Highly Oriented Void Channel Hydrogels

The primary role of hydrogels in most applications is to regulate transport through pores, which is done by varying size and pore distribution; the porous structure of hydrogels plays a crucial role in regulating the transport of nutrients, gases, and waste and providing physical space for cell diffusion and communication, facilitating the formation of the final tissues. Conventional hydrogels are limited to a pore size of 100 nm, which hinders the potential for TE applications requiring micrometer-scale cell permeation. While some template-sacrificing methods, such as salt/porogen templating and gas foaming, can create hydrogels with micron-scale pores, they lack control over the interconnectivity and directionality of the pore network. Creating highly oriented continuous void channel hydrogels, makes it possible to better mimic natural biological tissues and more efficiently direct solute diffusion or transport.

#### 2.3.1. Directional Freeze-Casting for Preparing Highly Oriented Void Channel Hydrogels

Directional freezing, also known as ice templating, freeze casting, or ice separation-induced self-assembly, involves freezing a pre-reaction solution in a cold bath along a unidirectional temperature gradient to form ordered ice crystals that grow in the freezing direction. As one of the sacrificial template methods, the solute material repels from the ice and separates into the interstitial region defined by the curing solvent during ice crystal formation, while the ice crystals sublimate and then form highly oriented 3D pore channels along the freezing direction without disturbing the interstitial region. The orientation, number, morphology, and size of the porous or channel structure can be controlled by several factors, including solvent content, freezing temperature, freezing rate, and ice template support. Nucleation theory is the fundamental principle used to control pore size and the number of pores; lower freezing temperatures and faster freezing rates result in an increase in hydrogel nucleation sites and the formation of a large number of smaller ice crystals, while at higher freezing temperatures, the distribution of nucleation sites becomes looser, and the ice crystals increase in size. Lowering the polymer/solute concentration can also produce larger pore sizes. This method is environmentally beneficial compared to other methods for preparing highly oriented hydrogels, and since most hydrogels are made from aqueous precursor solutions, it is relatively easy to control highly oriented ice crystallization. Therefore, this simple, effective, and controllable method is commonly used to prepare hydrogels with highly oriented pore channels by templates and has been widely used recently to design highly oriented void channel hydrogels with various functions.

**Figure 9:**
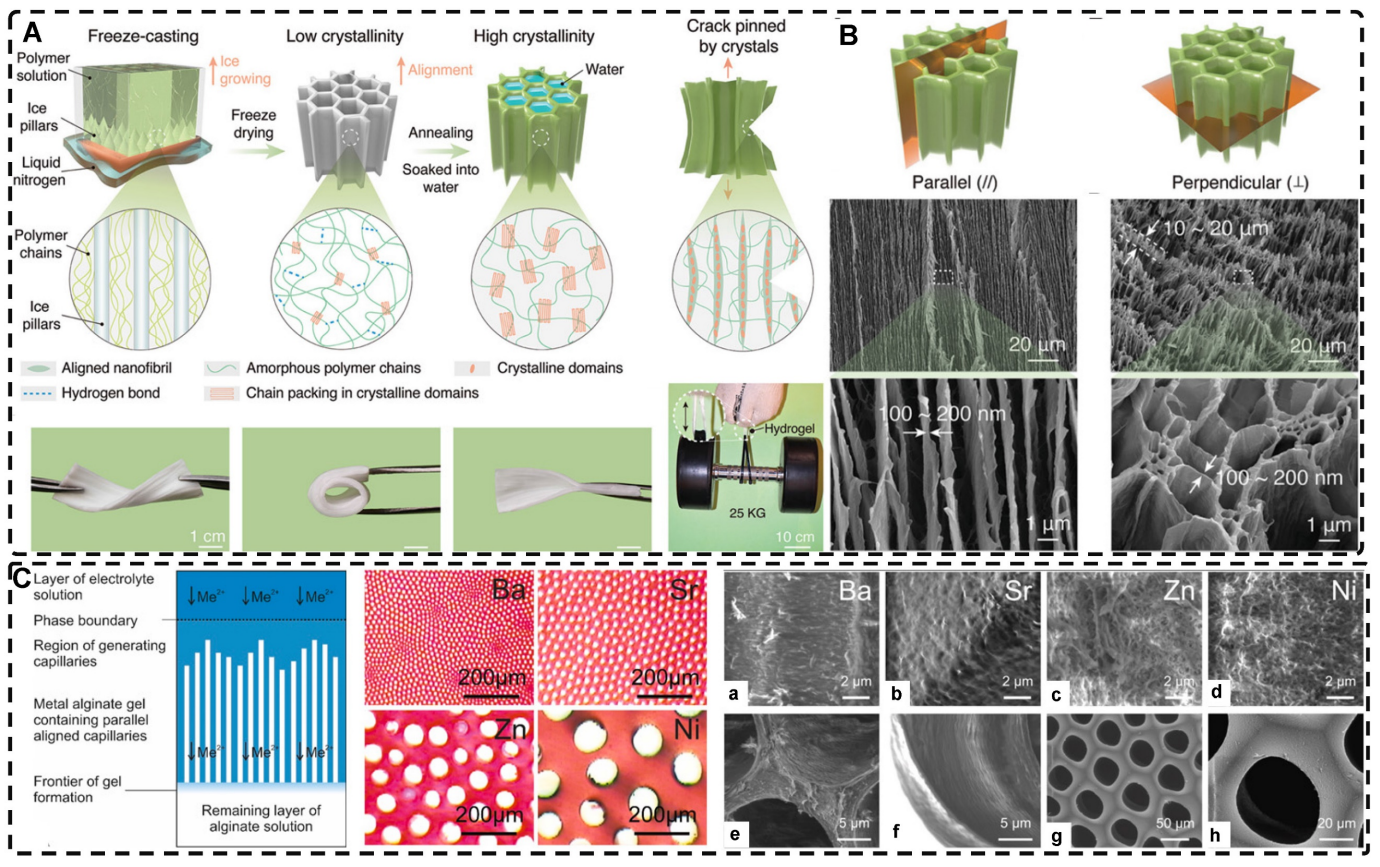
A) Schematic illustration for the highly oriented void channels hydrogels fabrication by using an ice-template freeze casting process. B) SEM images of the highly oriented void channels hydrogels. Adapted with permission from 198, copyright 2021, Wiley‐VCH. C) Schematic drawing of the void channels formed by unidirectional diffusion of divalent metal cations (Me^2+^) into a solution of sodium alginate, and cross-sections of the void channels in hydrogels formed by different cations. (a-h). Scanning electron microscopy images for highly oriented void channels hydrogels. Adapted with permission from 218, copyright 2022, Elsevier.

Since the initial report by Wu et al. 24 on preparing aligned porous hydrogels through unidirectional freezing, an increasing number of researchers have been using this method to develop highly oriented void channel hydrogels 162, 197-205. Liang et al. 198 used an ice-template freeze casting process to create highly oriented aligned micro/nanostructures in PVA hydrogels, followed by crystal growth initiated by thermal annealing** (Figure 9A-B)**. The resulting hydrogels exhibited excellent mechanical properties, high fatigue threshold, anti-crack sensitivity, and anisotropy, with a 100-fold increase in fatigue threshold compared to normal PVA hydrogels after swelling to equilibrium in deionized water. These properties make it a promising material for use in low-cost, high-performance, and durable soft applications such as robotics and artificial muscles. Additionally, this strategy is applicable to various hydrogel materials, including polysaccharides, proteins, synthetic polymers, and corresponding polymer composites. Hua et al. 199 synergistically created highly oriented void channel hydrogels at different length scales across multiple levels, from millimeter to molecular, by using directional freeze-casting, resulting in the Hofmeister effect and subsequent salting-out treatment. The researchers used PVA as the model material, and the solution was directly soaked in a kosmotropic salt solution after directional freezing. Directional freezing caused the PVA hydrogel to form highly directional honeycomb microchannels with arranged pore walls, and the polymer was concentrated and accumulated in the gap area between the channels, which was then ready for the subsequent strong aggregation and crystallization of polymer chains through salting-out. The preconcentrated PVA chains strongly self-coalesced and phase-separated from the original homogeneous phase under the influence of kosmotropic ions, which in turn formed a mesh-like nanofiber network on the surface of the micron-sized aligned pore walls. The hydrogels developed by this method exhibited high strength, toughness, tensile properties, and fatigue resistance. Since the Hofmeister effect is present in various polymer and solvent systems, this method may offer some assistance for other originally weak hydrogel applications in TE.

The subzero temperature environment during the fabrication of directed freeze casting may impair cell viability and limit the application of hydrogels intended to encapsulate functional cells in TE 206, 207, as the formation of ice crystals in the extracellular matrix results in an osmotic gradient in the cell membrane, leading to cellular dehydration and damage 208. Although cryoprotectants such as dimethyl sulfoxide and glycerol can be used, their concentration at low temperatures may cause osmotic shock or toxic damage to cells and lead to adverse effects in humans, such as clonic seizures and cardiac arrest 208, 209. Therefore, the development and exploration of advanced cell cryopreservation agents, such as vitrification agents, ice recrystallization inhibitors, macromolecular cryoprotectants, ice nucleating agents, and regulation of biochemical pathways, will contribute to expanding the applications of targeted cryogels in TE.

#### 2.3.2. Other Strategies for Preparing Highly Oriented Void Channels Hydrogels

Similar to the directional freeze-casting method, He et al. 210 developed highly oriented pore hydrogels using a crystal template technique called "hot ice",the principle of which is that needle-like and micrometer-sized aligned NaAc·3H_2_O crystals can form from saturated NaAc solutions at room temperature. A saturated NaAc and agarose mixture solution was prepared at a high temperature of 90°C, which was then cooled to room temperature to create an agarose gel in the supersaturated solution. Next, NaAc·3H_2_O crystals were placed on the surface of the supersaturated mixture solution to induce the formation of oriented crystals in the hydrogel in the vertical direction. Finally, the crystals were removed by multiple washes with water to obtain oriented pore hydrogels. The highly oriented pore hydrogels prepared using this technique exhibited great potential in TE due to the resulting high survival rate of cultured NIH3T3 cells and linear alignment.

The physical template method is a common technique for preparing highly oriented void channel hydrogels. This process involves polymerizing the hydrogel precursor solution in a mold which then determines the orientation and shape of the void channels in the hydrogel. For instance, Chen et al. 211 were inspired by the preparation of oriented pore PLGA scaffolds and used a mold comprising seven uniformly distributed stainless steel rods and glass tubes to fabricate oligo(polyethylene glycol) fumarate (OPF) and positively charged OPF (OPF+) hydrogel scaffolds with oriented void channels. These hydrogels were designed to closely match the mechanical properties of rat spinal cord tissue, and animal studies showed that OPF+ hydrogel promoted a unique pattern of axonal regeneration with a large number of regenerated axons concentrated in the channel. Subsequent studies have explored the potential value of such hydrogels loaded with cells or growth factors for neurological and spinal cord injuries 212-214. To further improve tissue integration and uniaxial tissue growth, Dumont et al. 215 utilized a two-step cross-linking process to modularly assemble hydrogel tubes prepared via templates. The multiple void channels of the hydrogel tubes promoted tissue integration while the directional void channels supported uniaxial tissue growth, which is crucial for highly structured tissue regeneration. Hydrogel tubes can be easily customized by cutting and packaging to the desired size and shape with directed regenerative void channels. This strategy has the potential for personalized repair of spinal cord, peripheral nerve, muscle, and vascular tissue defects.

Directed ion diffusion can also be used to prepare highly oriented pore channels in hydrogels. Weidner et al. 216, 217 achieved this by using electrolyte solutions containing Ba^2+^, Sr^2+^, Zn^2+^, and Ni^2+^ cations to prepare highly oriented capillary pore hydrogels with capillary sizes ranging from 12-100 μm. The oriented capillary pore channels were formed through a self-organizing process driven by the unidirectional diffusion of divalent cations into sodium alginate sols, opposite diffusion gradients, and the dissipation of the driving force of convective processes during friction of alginate chains. This led to the continuous precipitation of metal alginate, resulting in highly oriented capillary gels with honeycomb-like structures. The mechanical properties and chemical stability of this hydrogel were recently studied by Müller et al. 218, who found that the mechanical properties and degradation stability can be varied by the degree of chemical cross-linking and the formation of interpenetrating networks **(Figure 9C)**. The higher the concentration of cross-linking agent, the higher the mechanical strength. Additionally, the mechanical strength of the hydrogel generally decreased with an increase in capillary pore diameter, and the concentration of the cross-linking agent positively correlated with the resistance of the hydrogel to degradation.

3D printing enables the fabrication of hydrogel structures with highly oriented pore channels of various dimensions and directions. Li et al. 219 utilized digital light processing-based 3D printing to produce HA gelatin-based hydrogels with controlled and oriented microchannels, and they investigated the impacts of different microchannel sizes on NSC neural stem cell viability and proliferation. The outcomes demonstrated that directional microchannels could direct the migration of NSC spheroids, and the aligned microchannels could constrain the neuronal growth of NSCs within the channels, promoting alignment and preventing disorganization. Furthermore, 3D printing could accurately match the size of the hydrogel to the size of the lesion area, offering promising potential for personalizing TE treatments, with the highly oriented channel structure facilitating directed cell growth.

Hydrogels with highly oriented void channels facilitate the transport, diffusion, and growth of macromolecules like growth factors or cells due to the presence of micrometer or larger pore channels compared to void-free channel hydrogels. However, their inherently poorer mechanical properties compared to homogeneous void-free hydrogels can limit their use in load-bearing tissues 199. Template-sacrifice methods such as directional freeze-casting methods are simple, easy, economical, and environmentally friendly but do not provide precise geometric control of the material's dimensional characteristics compared to techniques like 3D printing. Additionally, accurately simulating natural tissue organs remains a challenge. In the future, hydrogels with highly oriented pore channels should aim to improve their mechanical strength and achieve more precise bionic direction.

### 2.4. Highly Oriented Hydrogels with Microfabricated Structures

#### 2.4.1. 3D Printing for Preparing Highly Oriented Hydrogels with Microfabricated Structures

**Figure 10:**
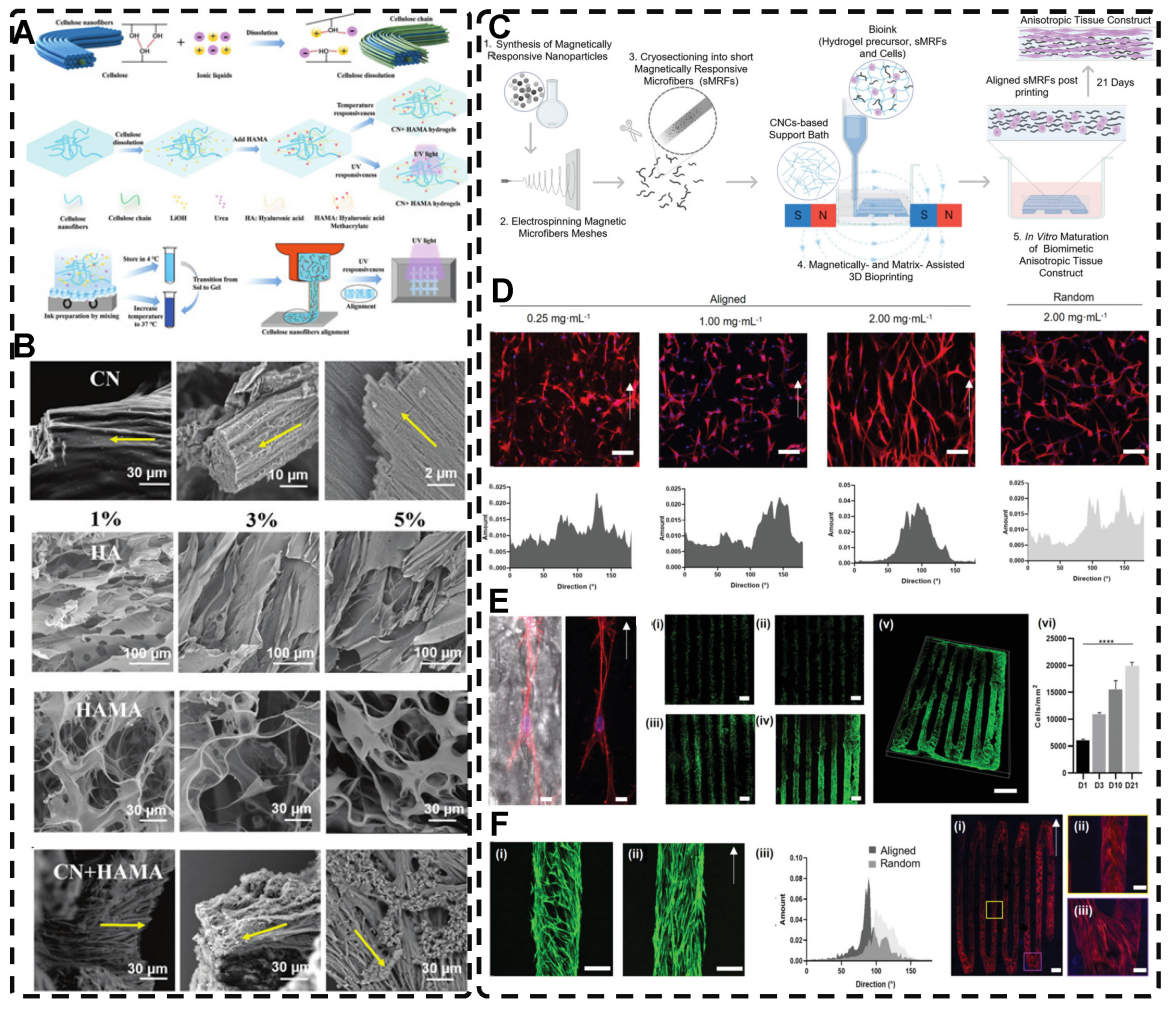
A) Schematic illustration of the composition and synthesis mechanism of the cellulose nanofibers + hyaluronic acid methacrylate (CN+HAMA) highly oriented hydrogels. B) SEM images of CNs, hyaluronic acid (HA), HAMA, and CN+HAMA hydrogels with different concentration ratios. Adapted with permission from 25, copyright 2022, AccScience Publishing. C) Schematic illustration of the proposed strategy to fabricate high-resolution oriented biomimetic constructs. D) The effect of short magnetically-responsive microfibers (sMRFs) orientation and concentration over the alignment and morphology of the encapsulated hASCs, along with their orientation plots. E) CLM images of hASCs adhered to sMRFs and acquiring elongated morphologies in highly oriented hydrogels, and viability and orientation of the hASCs encapsulated within oriented hydrogel after i) day 1, ii) day 3, iii) day 10 and iv/v/vi) day 21 of cell culture. F) Cell viability after 21 days in 3D bioprinted hydrogels with 2.00 mg·mL^-1^ of i) randomly-oriented sMRFs and ii) aligned sMRFs. G) CLM images of hASCs cytoskeleton in GelMA, i) tile scan of fabricated construct, ii) straight line of the printed filaments, iii) curve of the printed filaments. Adapted with permission from 227, copyright 2022, Wiley‐VCH.

Although the fabrication strategies for highly oriented hydrogels discussed in the previous sections have a wide range of applications, many of these strategies lack precise control over the hydrogel's structure, which is crucial for TE applications of hydrogels. Therefore, 3D printing is a promising method for creating complex tissue structures with high precision and reproducibility. This technology is increasingly being explored and applied due to its ability to prepare biomimetic hydrogels efficiently and individually according to the biological tissue structure in both micro- and macro-structures. The current techniques widely used for hydrogel 3D printing are mainly classified into extrusion printing, inkjet printing, stereolithography printing, and laser-assisted printing methods 220-223. Many reviews have examined or reviewed the details of these techniques in detail, so this paper does not discuss them in depth 223, 224. Among these techniques, extruded 3D printing is the most widely used, with its ability to prepare highly oriented filler/polymer chain/void channel hydrogels at microscopic and macroscopic levels 225, 226. As described in Section 2.1, the shear and tensile forces of the printing nozzle can orient the distribution of the filler material within the hydrogel. For example, Dong et al. 25 prepared an oriented CN+HAMA hydrogel using extrusion-based 3D printing. Similarly, shear forces during the printing process can cause the hydrogel polymer-chains to align in a highly directional manner **(Figure 10A-B)**. Kim et al. 192 prepared highly oriented collagen hydrogels with different cells using the shear stress of 3D printing nozzles. The physical topographical and biochemical cues carried by the highly oriented collagen hydrogels synergistically induced attachment, growth, and alignment of H9C2, MC3T3-E1, and hASC. Furthermore, shear force-induced cell alignment without any loss of cell viability was achieved, presenting a new approach to TE regeneration. The preparation of hydrogels with oriented pores by 3D printing has been discussed in Section 2.3.2 and will not be covered here.

In addition to utilizing shear forces alone, 3D printing can also be used in conjunction with other external fields to prepare highly oriented hydrogels. Pardo et al. 227 combined magnetically and matrix-assisted 3D bioprinting strategies to extrude bioinks composed of GelMA, short magnetically responsive microfibers (sMRFs), and hASCs into a support bath based on CNCs hydrogels, under the presence of a low-intensity magnetic field **(Figure 10C-G)**. This resulted in the preparation of hydrogels filled with highly oriented alignments of sMRFs. The high magnetic response of the MNPs bound in the sMRFs allowed them to obtain orientation by remote alignment in low viscosity GelMA inks at low content of magnetic material condition by weaker magnetic field strength (14±2 mT). The structure of the hydrogel, as well as the ability of magnetomechanical stimulation to induce the oriented growth of encapsulated hASCs, and the subsequent addition of tendon dECM (decellular extracellular matrix) to the hydrogel to induce differentiation of hASCs toward tenogenic lineage, provide a new strategy for the reconstruction of natural tendon tissue and the maturation of bioengineered tendon constructs. While the hydrogel was designed with the principle of satisfying the regenerative reconstruction of tendon tissue, it also serves as a design reference for exploring the application of other oriented tissues, such as skeletal muscle, cartilage, and cardiac muscle.

Incorporating cells or stem cells into bioink is a promising research direction toward biocompatibility. Inducing cellular orientation in hydrogels through shear force by extrusion 3D printing is another important research focus. The cells within the highly oriented hydrogel can grow directionally along the highly oriented structure, better mimicking the aligned tissue found in the human body. However, the inevitable shear forces during the preparation process can damage or kill the cells and reduce their viability after 3D printing. Mechanosensing, mechanotransduction, and downstream mechanical response pathways are the three primary processes that occur in the cellular response to mechanical stresses, such as shear force, which integrate multiple biochemical signals from sensing and transduction events in time and space. Although shear stress can have negative effects on piggybacking cells, it plays a crucial role in controlling cell growth, motility, contraction, and differentiation into specific tissues or organs. For example, suitable shear stress can induce the differentiation of stem cells into vascular endothelial cells and osteogenic cells while controlling directional cell alignment. To achieve predefined 3D hydrogel scaffolds that mimic living tissues and organs and potentially shorten the length of organ transplant lists, scientists are exploring the balance between material properties, printing parameters, and the different cells used. This direction of research holds promise for the future of TE and 3D printing technologies. To date, there is no bioprinting technology capable of creating synthetic tissues and organs at all levels of complexity, which is the ultimate goal of 3D bioprinting in TE. Although 3D printing for TE organ transplants is still in the early stages, the ultimate goal is expected to be closer to reality with the advancement of printing technology (resolution, printing speed, consistency reproducibility, etc.) and the selection of suitable printing materials.

#### 2.4.2. Other Strategies for Preparing Highly Oriented Hydrogels with Microfabricated Structures

**Figure 11:**
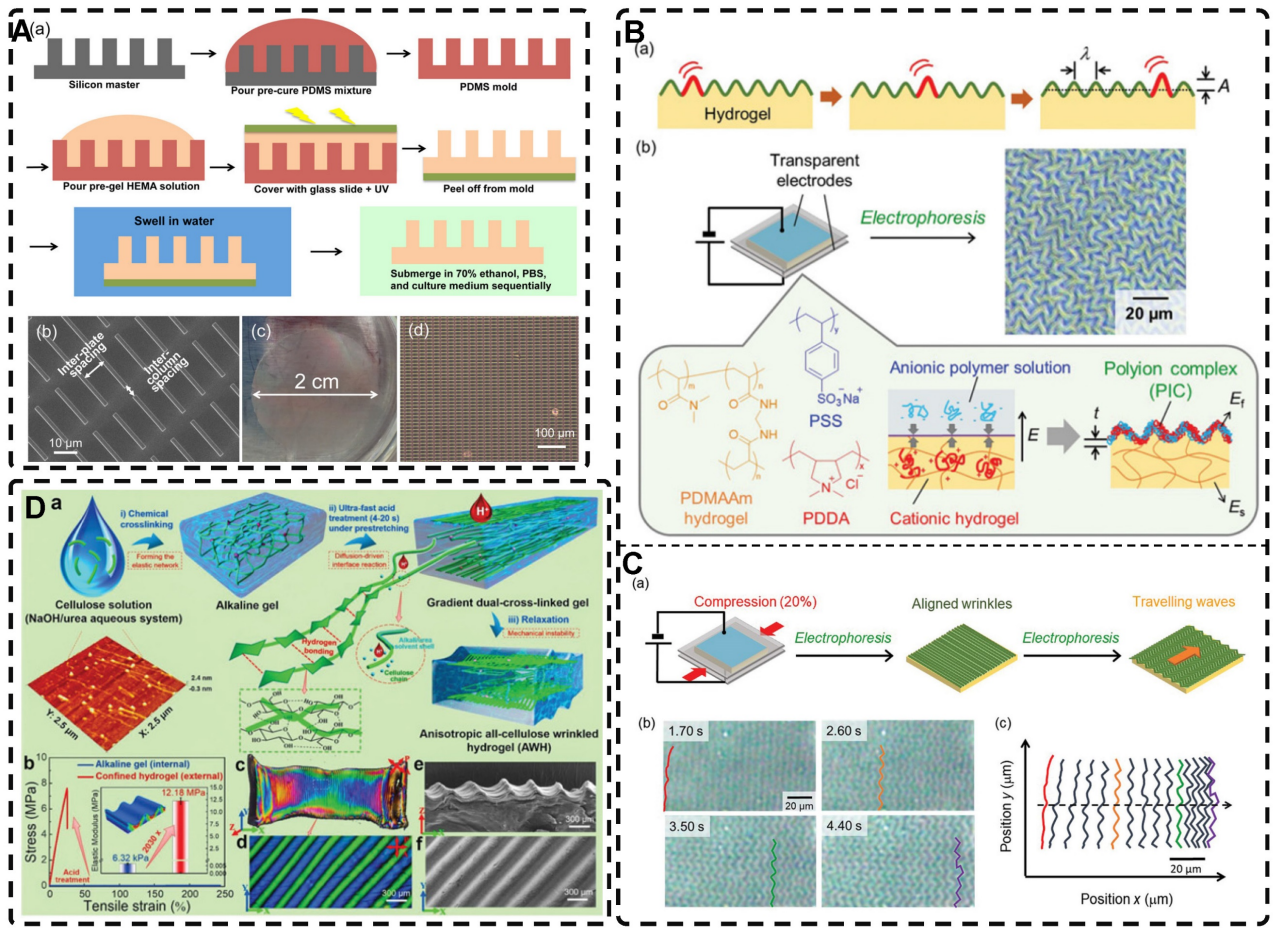
A) (a) Schematic illustration of the procedure for the fabrication of the microstructured highly oriented hydrogel substrate. Scanning electron microscopic image (b), picture (c) and microscopic image (d) of hydrogel. Adapted with permission from 230, copyright 2016, American Chemical Society. B) a) Schematic of Travelling wave generation of PIC wrinkles on the hydrogel surface. b) Fabrication of wrinkles on a hydrogel surface by electrophoretic. C) a) Experimental electrophoretic formation of aligned wrinkles under the lateral compression of gels. b) Time-course images of aligned wrinkle formation during electrophoresis. c) Trajectories of aligned wrinkle as a function of time. Adapted with permission from 239, copyright 2022, Wiley‐VCH. D) a) Schematic image of highly oriented hydrogels fabrication. b) Differences in mechanical properties between the outside and inside of hydrogels. c,d) Birefringence patterns and magnified POM images of an hydrogels sample. e,f) Cross-section and surface SEM images of hydrogels showing a long-range and highly oriented self-wrinkling configuration. Adapted with permission from 240, copyright 2019, Wiley‐VCH.

Creating highly oriented topographic patterns on the surface of hydrogels is a method employed to generate microstructured, highly oriented hydrogels. Among the various methods used to shape highly oriented hydrogels, molding is a well-established and straightforward approach 228-231. For instance, Hu et al. 230 employed a silicon master to fabricate a Polydimethylsiloxane (PDMS) mold, which was then used to create a highly oriented surface morphology pattern on a poly(2-hydroxyethyl methacrylate) (pHEMA) hydrogel** (Figure 11A)**. This highly oriented morphology pattern can promote the adhesion, spreading, and elongation of human MSCs cultured on the hydrogel, serving as a foundation for the future development of highly oriented hydrogels for TE applications.

Wrinkling can give rise to highly oriented surface morphologies in hydrogels 232-234. Professor Rodríguez-Hernández introduced the wrinkling model and proposed a comprehensive mechanism 235. The Allen model 236 established the theoretical foundation for orientational wrinkling, and Bowden et al. 237 developed an anisotropic wrinkling model that correlates spatially inhomogeneous stress distributions with wrinkling patterns to analyze the characteristics of wrinkles accurately, including their wavelength and direction 238. Kashihara et al. 239 employed electrophoresis to create a polyion complex (PIC) layer on the hydrogel surface **(Figure 11B-C)**. The resulting stress mismatch between the two layers led to the formation of wrinkles as the PICs deformed on the hydrogel surface. Applying uniaxial compression to the hydrogel in the planar direction enabled control over the direction of wrinkle motion during the formation process, resulting in a highly oriented wrinkled morphology. Ye et al. 240 immersed pre-stretched cellulose alkaline gels into acidic solutions for varying durations, resulting in the formation of a dense nanofiber structure and a robust dual-crosslinked hydrogel after the removal of external forces **(Figure 11D)**. In this process, the physical cross-linking of the pre-stretched aligned cellulose chains within the acidic solution induced mechanical instability in the hydrogel. The removal of the stretching led to the formation of a highly oriented self-wrinkling surface structure due to this mechanical instability. The highly oriented wrinkled structure of the hydrogel enhanced the adhesion and alignment of MC3T3 cells. The programmability and biocompatibility of this hydrogel, along with its design concept, make it a valuable material for application and exploration in TE. The wrinkling method offers a simple, rapid, and cost-effective approach for producing large-area hydrogels with oriented surface morphologies, thereby effectively generating multi-scale, highly oriented hydrogels.

### 2.5. Highly Oriented Hydrogels Prepared by Special Methods

**Figure 12:**
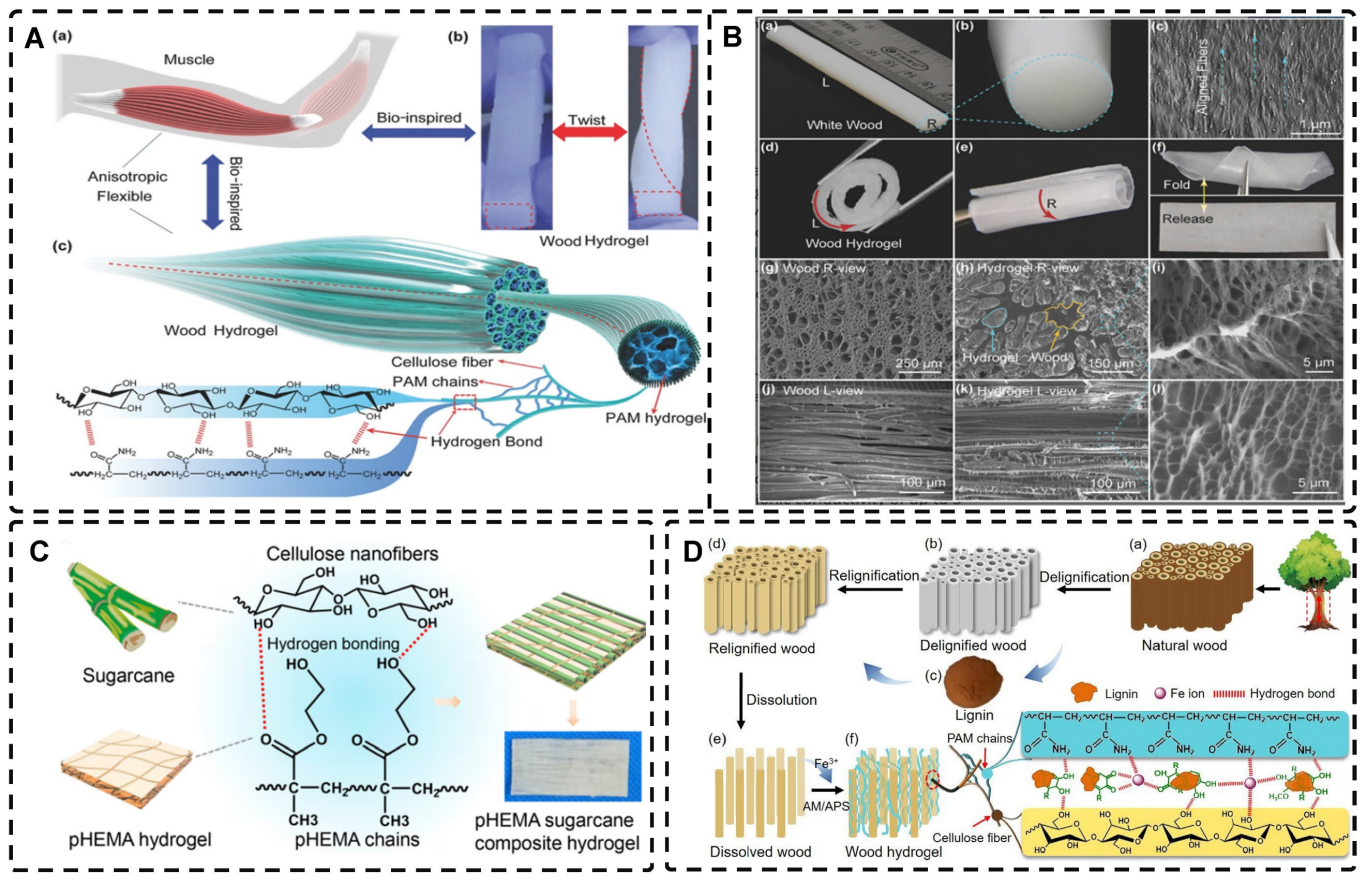
A) a) Schematic of a conventional flexible and striated skeletal muscle tissue to be emulated by the wood hydrogel. b) Images of a 7 cm long wood hydrogel sample being twisted 180°. c) Depiction of the wood hydrogel hydrogen bonding and covalent cross-linking between PAM chains. B) Optical (a-f) and SEM images (g-i) of the wood hydrogel and white wood. Adapted with permission from 241, copyright 2018, Wiley‐VCH. C) Schematic illustration of hydrogen bonding formed between white sugarcane and pHEMA chains. Adapted with permission from 242, copyright 2021, Wiley‐VCH. D) Schematics illustrating the all-wood hydrogel preparation (a-f). Adapted with permission from 243, copyright 2021, Elesevier.

Many natural organic materials have a highly oriented extracellular matrix with excellent mechanical properties, making them ideal templates for developing highly oriented hydrogels with improved mechanical properties. Hu et al. 241 utilized natural wood as a biomold to fabricate hydrogels that mimic muscle tissue. They removed lignin from white wood by a delignification process in a NaClO_2_ solution at pH=4.6 to release the tight connections between fibers while maintaining the inherent structure of oriented aligned cellulose nanofibers (CNFs). The acrylamide (AM) monomer effectively formed strong hydrogen bonds with the CNF backbone in the presence of an initiator and cross-linker to develop layered highly oriented composite wood hydrogels. The high tensile strength of 36 MPa in the longitudinal growth direction was attributed to the wood backbone, and the strong interfacial bonding provided favorable mechanical properties **(Figure 12A-B)**. Furthermore, the hydrogel's anisotropic optical properties and excellent ionic conductivity make it attractive for various applications, including TE. Xu et al. 242 utilized natural sugarcane as a biotemplate to create three highly oriented sugarcane composite hydrogels that form hydrogen bonds with different materials: pHEMA, poly(acrylamide) (pAM), and poly(acrylamide-co-acrylic acid) (p[AM-co-AA])** (Figure 12C)**. These hydrogels exhibited good flexibility, elastic recovery, and anisotropic lubrication behavior, making them promising candidates for the development of artificial skin and cartilage-lubricating materials. Similarly, Yan et al. 243 developed highly oriented all-wood tough hydrogels through dynamic bonding between natural wood biotemplates, PAM chains, and iron ions, by exploiting the reversible crosslinking of catechol groups of lignin in natural wood with Fe^3+^ ions under the influence of Ammonium peroxydisulfate (APS), which also acted as a crosslinker to link the cellulose fibers in the highly oriented wood skeleton to the PAM chains **(Figure 12D)**. The resulting hydrogel exhibited a wide range of strain, high sensitivity, and good flexibility due to the reversible hydrogen bonding and metal coordination bonding, making it an excellent candidate for compressive sensing. It is believed that the technique of using natural biological templates to create highly oriented hydrogels has a promising future in TE applications 238.

**Figure 13:**
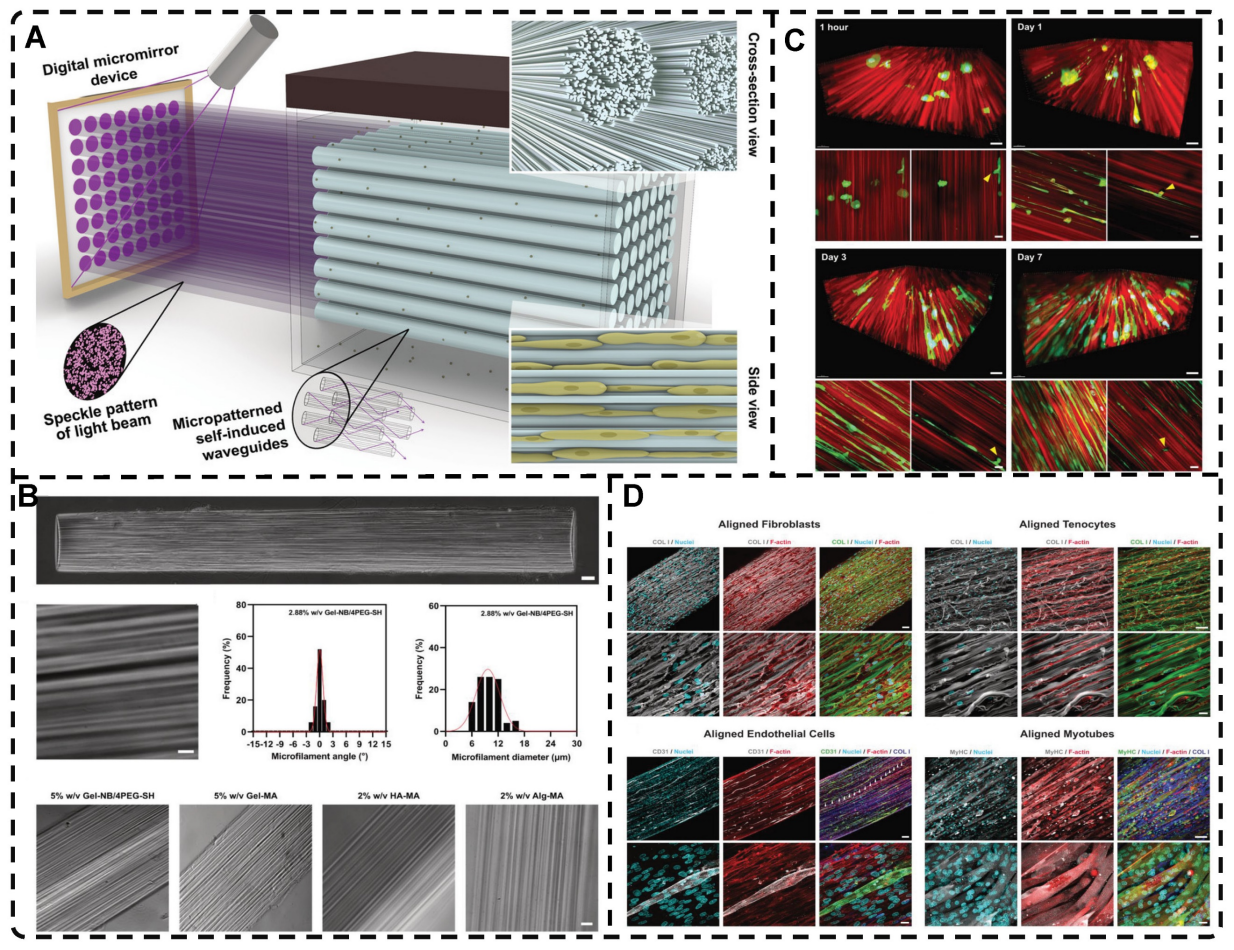
A) Schematic illustration of FLight strategy for fabricating highly oriented microfilaments and their cell guidance properties. B) Bright-field image of FLight hydrogel construct, magnified image of microfilaments structuring of oriented hydrogel, distribution of the microfilament diameter, and the distribution of angles' difference between the orientation of microfilament and direction of projection. C) Fluorescence images of cell-laden hydrogel matrix. D) Cell guidance properties of microfilaments and maturation of highly aligned tissue-engineered constructs. Evidence of highly oriented fibroblasts, tenocytes, endothelial cells, and myotubes by immunofluorescence staining. Adapted with permission from 30, copyright 2022, Wiley‐VCH.

Liu et al. 30 introduced the biofabrication technique "Filamented Light" (FLight) to produce hydrogels. They utilized optical modulation instability (OMI) to induce highly aligned hydrogel microfilaments through the interaction of spatially coherent light beams with various photoresin compositions **(Figure 13A-B)**. After removing the uncrosslinked photoresin, the voids between the microfilaments appear as microchannels with ultra-high aspect ratios, which can be used to guide cell alignment and migration **(Figure 13C)**. The diameter and spacing of hydrogel microfilaments could be adjusted by changing the coherence length of the beam from 2 to 30 μm. In less than 10 seconds, cells were safely and rapidly encapsulated in a highly oriented hydrogel microfilament network. Furthermore, other FLight biomanufacturing strategies such as Cross-Flight and Multi-Flight can been used to construct layered complex tissue constructs with different orientations, such as cardiac muscle tissue, by implementing multi-directional hydrogel microfilaments **(Figure 13D)**. This study was the first to apply this technique to the field of TE, and it offers promising prospects for future applications in highly oriented tissue regeneration, avoiding cellular shear stresses compared to extrusion-based 3D printing.

**Figure 14:**
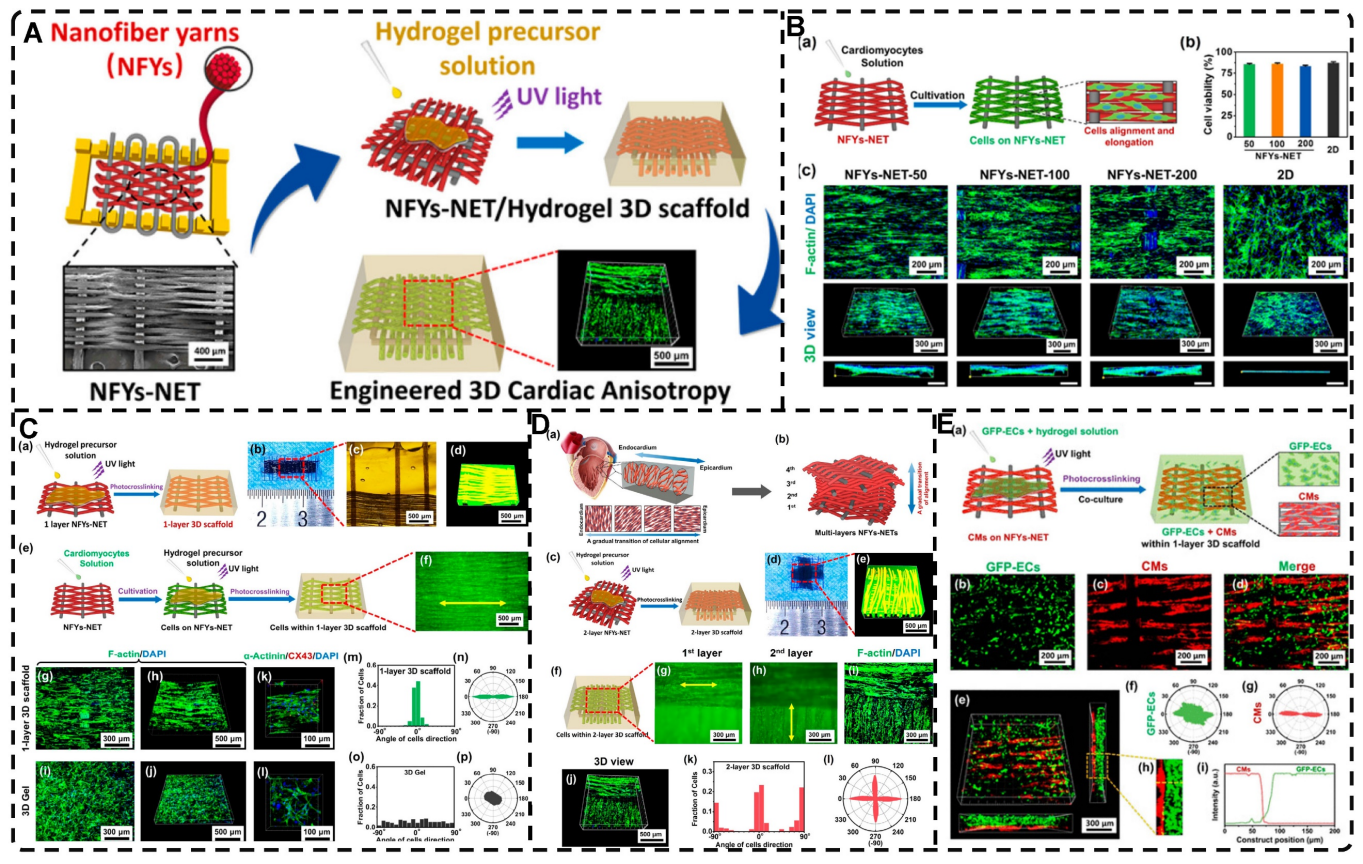
A) Schematic illustration of NFYs-NET hydrogel preparation. B) (a) Cardiomyocytes seeded and cultured on NFYs-NET hydrogels. (b) Relative cells viability percentages of cardiomyocytes on NFYs-NET hydrogels evaluated by live/dead assay. (c) The top views and 3D views of fluorescent images of cardiomyocytes on NFYs-NET hydrogels. C) (a-d) Fabrication process of 1-layer oriented hydrogels, and their gross image, optical image, and the 3D view of confocal image. (e, f) Scheme of cardiomyocytes seeded and cultured within hydrogels and the fluorescent image of cardiomyocytes. (g, h, k) The confocal images showed the cellular alignment and elongation within oriented hydrogels while (i, j, l) the random morphology of cells within GelMA hydrogel, and (m-p) the quantitative analysis of cellular orientation distribution. D) (a-c) Schematic of myocardium oriented structure, multilayers orientation of NFYs-NETs assemble, and 2-layer oriented hydrogels fabrication. (d, e) The gross image and 3D view of confocal image of 2-layer 3D scaffolds. (f-h) The scheme of culturing cells within oriented hydrogels and fluorescent images of cardiomyocytes with horizontal direction and vertical direction. (i-l) The top view and 3D view of confocal images of cells and the quantitative analysis of cellular orientation distribution. E) (a) CMs were cultured on the NFYs-NET layer, ECs were encapsulated within hydrogel shell. (b-d) The fluorescent images of ECs, CMs and their merge image. (e-g) The 3D view of confocal images of ECs and CMs within scaffolds, and the quantitative analysis of cellular orientation distribution. (h, i) The distribution of ECs in hydrogel and CMs on NFYs-NET. Adapted with permission from 246, copyright 2017, American Chemical Society.

The incorporation of highly oriented structures from electrostatic spinning products into hydrogels can bestow the hydrogels with desired structures 244, 245. For example, Wu et al. 246 devised a weaving technique to prepare a conductive nanofiber yarns network (NFYs-NET) structure using electrostatic spinning and a combination of polycaprolactone, silk fibroin, and carbon nanotubes. This structure was further encapsulated in a photocross-linked GelMA hydrogel to closely mimic the natural structure of heart tissue while imparting an oriented architecture to the hydrogel. The NFYs-NET demonstrated the ability to guide cell alignment and elongation, as well as enhance the maturation and function of cardiomyocytes (CMs). The GelMA hydrogel shells provided a favorable three-dimensional environment for mechanical protection and endothelialization by facilitating co-culture with endothelial cells (ECs). These three-dimensional hybrid scaffolds hold immense potential for applications in cardiac TE **(Figure 14)**.

## 3. Effect of Highly Oriented Structures on Cell Behaviors

Cells within natural physiological niches experience a multitude of cues, including physical stimuli such as mechanical, electrical, and topographical factors, as well as biochemical cues such as specific drugs, proteins, growth factors, and combined insoluble particles 247-249. These cues effectively direct cell behavior and function by regulating cell adhesion, migration, proliferation, and differentiation, thereby promoting tissue repair and regeneration 250-252. Highly oriented hydrogels can endow ordinary hydrogels with highly oriented structural cues, which may be analogous to physical cues being integrated and converted into intracellular signals through mechanical transduction that microcells can recognize in the microniche, ultimately influencing cellular behavior 26. The purpose of this part is mainly to analyze the crude cellular behaviors of highly oriented structures in highly oriented hydrogels affecting cell adhesion, migration, proliferation, and differentiation, and provide a brief review of the relevant literature. Despite their significance, the underlying biological mechanisms by which cells perceive and respond to these cues have so far remained incompletely understood and require further investigation. Limited research has explored cell behavior within 3D hydrogel systems with highly oriented structures. Consequently, discussions on the impact of the highly oriented structure of 3D hydrogels on cell behavior draw largely from insights derived from anisotropic materials, particularly micropatterned surfaces.

Cells cultured on highly oriented hydrogels or scaffolds exhibit robust adhesion, potentially attributed to specific topographical cues embedded within these structures, such as oriented ridges, grooves, or pores. At the macroscopic scale, topographical features influence cellular arrangement at the colony level 253, and at the microscale, topographies correspond to the size range of individual cells, thereby impacting cell behavior at the cellular level. The alignment and orientation of cells on microtopographical features are known as "contact guidance," a concept first proposed in 1964 254, 255. Moreover, at the nanoscale, topographical features manifest at a similar magnitude as cellular receptors, including integrins, influencing the adsorption of adhesion-related proteins 256.

The initial step in cell behavior is adhesion formation, which enables the cell to perceive and respond to the physical and functional properties of its external topographic environment, thus influencing cellular decision making 257. At the molecular level, cells detect topographical cues through integrins, which are transmembrane receptors located on thin protrusions of the cell membrane. Integrins consist of unique combinations of at least 24 α-subunits (18 types) and β-subunits (8 types) that interact non-covalently. These integrins form adhesion complexes and bind to ECM ligands, including RGD peptides 258. Integrins play a vital role in cell adhesion, serving as the primary family of cellular receptors responsible for interactions between cells and the ECM. Aside from anchoring cells to their surroundings, integrins also establish connections with the cytoskeleton within the cell, making integrin binding a crucial step in various intracellular signaling pathways 259. Integrins do not possess enzymatic activity; nevertheless, they have the capacity to recruit and accumulate multiple intracellular proteins at integrin clusters, forming integrin adhesion complexes (IACs) 260. These complexes serve as mechanical linkages between integrins and the actin cytoskeleton, playing a crucial role in directing cell spreading and fate. Focal complexes emerge as a result of IAC aggregation and subsequently mature into larger, elongated supramolecular complexes known as focal adhesions (FAs) 261. Within these complexes, certain proteins such as talin and vinculin participate in mechanosensitive events, crucial for adhesion maturation 262, 263. Other proteins, including paxillin, tensin, and p130cas, act as articulation proteins 264. Signaling molecules such as focal adhesion kinase (FAK) and Src family kinases are also present. Integrins and their complexes play a pivotal role in driving cellular functions and behaviors, encompassing cell adhesion, morphology, migration, proliferation, and differentiation. Topographical cues regulate these processes by modulating cell adhesion, migration, proliferation, and differentiation through integrin-mediated focal adhesions, which contribute significantly to mechanotransduction and the activation of intracellular signaling pathways.

The dynamic actin and microtubule cytoskeleton are key regulators of cellular morphology, undergoing reorganization via signaling pathways associated with guidance cue receptors 265. Initially, filamentous pseudopods respond to aligned topographic cues, and subsequently, adhesion is directed in the same orientation, leading to cytoskeleton alignment and cell rearrangement along the nanoscale guidance cue 266. Consequently, a highly oriented topography can influence filopodia orientation through spatially restricted adhesion sites, exerting anisotropic migratory forces on cytoskeletal arrangement. This results in cell elongation parallel to the hydrogel orientation and overall cytosol alignment. In neuronal cells, for instance, FAs play a crucial role in controlling axon initiation by orchestrating cell polarization, cytoskeletal organization, and axon pathfinding/motility 267. The contractile machinery of cells, mediated by Rho-associated protein kinase (ROCK), significantly influences their morphological responses to surface topography and substrate mechanics 268. Sun et al. 184 conducted an experiment where the addition of the ROCK inhibitor Y-27632 resulted in a reduced alignment of fibroblasts cultured on highly oriented hydrogels, demonstrating the impact of highly oriented structures on cell morphology. Ryosuke Ozasa et al. 269 further demonstrated a parallel relationship between cell orientation and FAs extending along oriented collagen scaffolds. Importantly, the cellular response to anisotropic topographic cues is dependent on cell type and can be influenced by cell-cell interactions and pattern density 270, 271.

Cell migration plays a crucial role in various physiological and pathological processes, and its induction is a vital aspect of TE and regeneration. Cells exhibit both individual and collective migration modes 272, 273. Individual migration can be categorized into two modes: mesenchymal and amoeboid. Mesenchymal migration, observed in fibroblasts, stem cells, and certain cancer cells, relies heavily on ECM adhesion, actin-based protrusions (lamellipodia or filopodia) at the leading edge, contraction of the actin network, and an elongated cell shape, resulting in slower migration 274. On the other hand, amoeboid migration, employed by various cells like primitive germ cells and immune cells such as leukocytes, features weak ECM adhesion and a transition of cell protrusions from lamellar and filamentous pseudopods to actin polymerization-driven rounded cell shapes and transient actin-free globular membrane protrusions known as blebs 275. Amoeboid migration relies on myosin-based contraction and pressure-driven cytoplasmic flow, enabling fast migration. In collective migration, signals from external cues are transmitted to the entire cell population through intracellular and intercellular signaling cascades, and mechanical transduction at cell-cell junctions and cell-ECM interfaces 276. Notably, cells can switch between individual and collective migration patterns, influenced by multiple factors, such as topographic features, ECM composition, adhesion strength, and biochemical signals 277, 278. However, the understanding of how highly oriented structures drive cell migration is still in its nascent stages.

Numerous studies have examined alterations in cell shape, protrusion shape, actin cytoskeleton, and motility when exposed to artificially created simulations of various topologies found in human-oriented tissues, including specific patterns or 3D aligned channels 279-281. However, there is a paucity of research investigating how the perception of these landscapes is integrated into cellular dynamics through downstream intracellular events. Several studies have reported that cells such as fibroblasts and neurons sense nano- to micrometer-scale oriented structures topographically through frontier protrusions to drive subsequent cell migration 282, 283. Recently, it has been discovered that local deformation or curvature of the plasma membrane at the cell-substrate junction serves as a sensor for nanomorphology. Bar family proteins, particularly FBP17, are considered pivotal in bridging the connection between nanomorphology and intracellular actin reorganization 284. This revelation of membrane curvature as a topological sensor fills a critical gap in understanding how surface topological signals trigger intracellular actin fiber reorganization. Moreover, topological cues also elicit changes in cell nuclear location and morphology, which can significantly impact cell migration 285. Kim et al. 286 investigated the effect of oriented structures on cells and found that NIH-3T3 cells cultured with oriented nanotopographic cues exhibited varying migration rates depending on topographic densities and orientations, demonstrated by the absence of growth factors during wound healing. Another study by Ray et al. 270 revealed that orientation-aligned structures, which provide contact-guided cues, physically restrict the maturation and orientation of FA, leading to directional traction, anisotropy, and orientation of actin fibers that drive cell orientation and directional migration.

The significance of cell proliferation in tissue regeneration is well-established, and the topographical cues provided by materials play a crucial role in regulating cell proliferation and differentiation. Previous studies have demonstrated that microtopography can effectively control the growth of neural cells and tissues, promoting enhanced cell proliferation and differentiation 287. Additionally, microtopography patterns have been shown to stimulate the proliferation of adherent cells. For instance, Seunghan Oh et al. 288 found that oriented titanium nanotubes significantly increased osteoblast proliferation by 300-400% compared to titanium metal surfaces. At the molecular level, inhibition of FA formation through modulation of the FAK/RhoA-regulated mTORC1 and AMPK pathway leads to reduced cell proliferation and metastasis 289. Consequently, integrins and their complexes are essential for cell proliferation; the adhesion of cells to the ECM through integrin receptors serves as an important checkpoint for entry into the cell cycle. Without proper integrin signaling, cells are unable to transition from the G1 phase to the S phase, preventing proliferation. The integrin adhesion complex activates multiple signaling cascades that facilitate cell progression to the S phase, including the Mek/Erk, PI3K/Akt, and the small GTPase Rac pathways 290. Scaffold proteins like talin can also play a role in proliferation by bridging mechanistic signals and nuclear responses. However, the mechanisms underlying the connection between actin polymerization and cytoskeletal changes during proliferation remain incompletely understood.

Controlling the nanomorphology of substrates presents an effective approach to modulating cell differentiation. For instance, Izadpanahi et al. 291 demonstrated that highly oriented aligned PLLA scaffolds could promote the expression of osteogenic marker genes in adipose MSCs (hMSCs), which randomly oriented scaffolds could not. At the molecular level, it was discovered that nanotopographical cues possess the ability to influence the osteogenic differentiation process of hMSCs. This influence is achieved through the modulation of lncRNAs and miR-125b, which serve as negative regulators of osteogenesis, as well as the H19 modulator BMP signaling pathway that functions as an miRNA sponge. In a similar vein, Lee et al. 292 prepared PLGA patches with highly oriented aligned nanogrooves, which exhibited enhanced migration and differentiation of osteoblasts in comparison to planar patches. In vivo studies conducted on rats further demonstrated the improved bone regeneration capabilities of the aligned nanogrooved patches. Furthermore, Xiao et al. 293 investigated the impact of distinct surface morphologies on the osteogenic differentiation of rat bone marrow-derived MSCs (rBMSCs) in the absence of osteogenic induction mediators. Their findings revealed that surfaces with oriented nano-grating-like films exhibited higher levels of osteogenic gene expression and distinctive activation of MAPK and Smad signaling pathways compared to flat and nanoporous films, which promoted the osteogenic differentiation of rBMSCs.

Previous studies have provided evidence linking FAs to the FA kinase and ERK pathways, both of which have the potential to influence cell differentiation 294. This suggests an effect of topography on cell differentiation through the mechanotransduction of extracellular biophysical signals to cells 265. Cells perceive topographical cues through integrins and transmit them to the nucleus via FA signaling and the actin cytoskeleton, thereby eliciting differential gene expression and promoting differentiation along specific lineages. For instance, anisotropic topologies can stimulate the osteogenic differentiation of cells by promoting the polymerization of FAs and actin. FAs have the ability to modulate cellular contractility and cytoskeletal organization, and an increased formation of FAs facilitates the osteogenic differentiation of cells 268, 295-297. The synergistic effects of cellular contractility, FAs, and YAP-TAZ signaling further enhance the osteogenic differentiation of cells 298, 299.

Indeed, the effect of highly oriented structures on cell behavior is a complex process involving multiple signaling pathways and the cytoskeleton. Understanding the mechanisms by which highly oriented structures affect cell behavior is essential for the development of novel biomaterials and therapeutic approaches in various research areas such as TE, regenerative medicine and cancer research. Further research is needed to fully understand the underlying mechanisms and to develop new approaches to improve cellular arrangement and function on highly oriented structures.

### 4. Application of Highly Oriented Hydrogels in Tissue Engineering

Biological soft tissues composed of highly oriented composite structures demonstrate pronounced anisotropic mechanical strength and functionality. Tissues and organs, such as the cornea, skin, skeletal muscle, tendons, ligaments, cartilage, bone, blood vessels, and heart, are composed of fibers and/or cells that possess a high degree of orientation, resulting in their distinctive structural orientation. While highly oriented tissues and organs share general orientation characteristics, each of them possesses unique composition, structure-related properties, and mechanical and biological functions. Therefore, the application of highly oriented hydrogels should be tailored to the specific properties of individual tissues or organs, and the corresponding hydrogels should be customized accordingly.

This section provides a comprehensive review of the existing research on highly oriented hydrogels in the field of TE. Furthermore, it offers a concise overview of the primary orientation characteristics exhibited by different tissues and organs, setting the stage for further discussion.

### 4.1. Highly Oriented Hydrogels for Cartilage Tissue Engineering Applications

Cartilage, a vital connective tissue in the body, is composed of 10-15% chondrocytes and ECM. The ECM of mature articular cartilage primarily comprises type II collagen (90%), with smaller proportions of type XI (3%) and type IX (1%) collagen. Cartilage serves as a crucial contributor to joint function by providing a frictionless and lubricated surface 44, 300. Cartilage exhibits a hierarchical structure characterized by distinct orientations of collagen fibers: a superficial layer with parallel orientation to the cartilage surface, a middle layer with randomly arranged fibers, and a deep layer with fibers oriented perpendicular to the surface 301, 302 Each layer's specific orientation corresponds to distinct functional roles: the superficial layer bears shear forces generated by joint motion, the middle layer serves as the primary cushion against resistance, and the deep layer provides exceptional compression resistance 44. These three layers collaborate synergistically to enable cartilage to effectively dissipate compressive loads and absorb impact forces, thereby safeguarding and facilitating bone movement 303.

**Figure 15:**
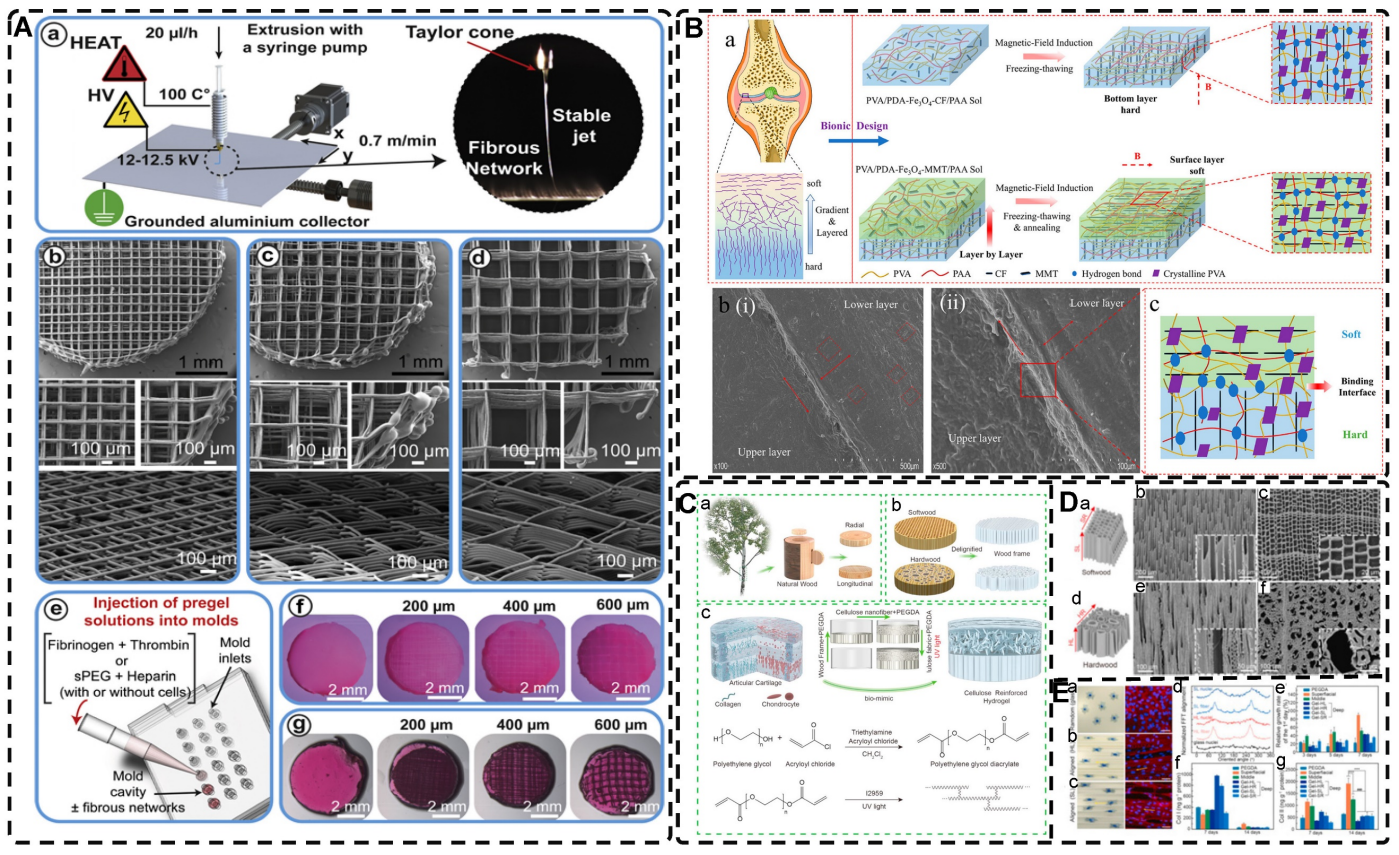
A) (a) Schematic of preparation of highly oriented mPCL fibers. (b-d) Different fiber spacing of the printed fibrous networks. (e) Schematic of preparation of highly oriented hydrogels, and their stereomicroscopy images. Adapted with permission from 245, copyright 2017, IOP Publishing. B) (a) Schematic illustration of the bilayer oriented heterogeneous hydrogel (BH-CF/MMT hydrogel). (b) SEM images of the BH-CF/MMT hydrogel. (c) Schematic illustration of the binding interface of the BH-CF/MMT hydrogel. Adapted with permission from 309, copyright 2022, American Chemical Society. C) (a) Schematic illustrates two types of wood frames that can be cut from a natural tree trunk. (b) The process of delignification. (c) The fabrication process of the bio-inspired three-zone highly oriented composite hydrogel. D) (a, d) Schemes of softwood and hardwood structure. (b, e) Longitudinal direction cross-sectional and (c, f) radial direction cross-sectional SEM images of the softwood and hardwood with aligned channels. E) (a-c) Schemes and cell morphologies of actin filaments and nuclei on the surface of glass slide, HL and SL. (d) 2D-FFT curves of the fluorescent images cultured on different material surfaces. (e-g) BMSCs relative proliferation rate and the relative content of collagen I and collagen II of different zone hydrogels. Adapted with permission from 312, copyright 2020, Elsevier.

Healing cartilage defects larger than 2 mm in diameter pose a significant clinical challenge due to the lack of blood vessels and nerves within natural cartilage tissue 304. Numerous tissue-engineered scaffolds have been employed to fill defects and promote cartilage regeneration 303, 305-308. By replicating the orientation of natural cartilage, highly oriented hydrogels can effectively emulate the anisotropy of the native tissue. Bas et al. 245 utilized a network of highly oriented polycaprolactone (mPCL) fibers prepared via melt electrospinning to reinforce a star-shaped poly(ethylene glycol)/heparin hydrogel (sPEG/Hep) **(Figure 15A)**. This approach aimed to replicate the biomechanical characteristics of human articular cartilage, encompassing mechanical anisotropy, nonlinearity, viscoelasticity, and morphology. Furthermore, it provided an appropriate microenvironment for chondrocyte culture and facilitated the in vitro formation of new cartilage tissue. Liu et al. 32 fabricated CNC/collagen nanocomposite hydrogels with hierarchically oriented pores, including horizontally aligned, randomly aligned, and vertically aligned orientations, which replicated the structural characteristics of natural articular cartilage through the process of directional freezing. Dai et al. 201 developed a highly oriented hydrogel using Silk fibroin (SF), poly(ethylene glycol) (PEG), and CNCs through the process of directional freezing. The resulting hydrogel exhibited a Young's modulus of 709 kPa and 160 kPa, which is comparable to the range (0.4-0.8 MPa) observed in natural cartilage. Chen et al. 309 fabricated bionic cartilage hydrogels with hierarchical orientation using magnetic field orientation technology, the freeze-thaw method, and the annealing process. The hydrogels consisted of a horizontally oriented surface layer made of PVA/3 wt % PDA-Fe_3_O_4_-MMT/PAA hydrogels and vertically oriented deep layer made of PVA/3 wt % PDA-Fe_3_O_4_-CF/PAA hydrogels **(Figure 15B)**. The materials employed include PVA, montmorillonite (MMT), Polyacrylic acid (PAA), and Carbon fiber (CF). The layered, oriented hydrogels demonstrated excellent frictional properties in the surface layer and high load-bearing capacity in the bottom layer, closely resembling natural cartilage. This innovative approach offers a novel strategy for the design of cartilage-like hydrogels. Furthermore, these highly oriented hydrogels hold significant clinical translational value as they can induce the aligned alignment of cultured cells while maintaining their natural mechanical properties. Wu et al. 310 developed a composite oriented hydrogel by incorporating a radially oriented poly(lactide-co-glycolide) (PLGA) scaffold into an oxygen species (ROS)-scavenging hydrogel (RS Gel). Upon implantation, the oriented hydrogel exhibited excellent biocompatibility, anti-inflammatory properties, and natural cartilage-like tidemarks. Moreover, the abundant chondrocytes within the hydrogel were arranged in a well-ordered manner, closely resembling the orientation observed in natural cartilage tissue. However, during the initial stage, the composite hydrogel somewhat influenced cell infiltration into the hydrogel; adding bioinductive factors, such as growth factors, may enhance the optimization of the scaffold. Gossla et al. 311 fabricated composite highly oriented hydrogels by combining oriented chitosan scaffolds with alginate hydrogels. This combination resulted in improved compressive strength and elasticity, specifically in the direction of the oriented fibers, offering superior mechanical stability when compared to chitosan scaffolds or pure alginate hydrogels. Moreover, the oriented hydrogels promoted chondrogenic differentiation of primary human chondrocytes, upregulated the expression of chondrogenic markers at both the RNA and protein levels, and effectively reduced the synthesis of type I collagen. Wang et al. 312 utilized cellulose fabric, cellulose nanofiber, and wood cellulose fiber in combination with a poly(ethylene glycol) diacrylate (PEGDA) polymer matrix. These components were polymerized using UV radiation to fabricate hierarchically oriented hydrogels that mimic the structure of cartilage** (Figure 15C)**. The composite layered, oriented hydrogel exhibited a shallow fiber morphology parallel to the hydrogel surface, a randomly distributed fiber morphology in the middle region, and deep reinforcing fibers oriented perpendicular to the hydrogel surface **(Figure 15D)**. The hierarchical orientation structure imparted distinct nonlinear and viscoelastic mechanical properties to the composite hydrogel across different regions. The highly oriented superficial and deep regions promoted the alignment of mouse bone-derived mesenchymal stem cells (BMSCs), resulting in predominantly hyaline cartilage differentiation across all regions after 14 days **(Figure 15E)**, which exhibited comparable morphology and collagen II content distribution to natural cartilage. Furthermore, the presence of channel-like wood cellulose fiber frames in the deeper regions created capillaries, enhancing nutrient and cell transport within the hydrogel, which played a crucial role in facilitating cartilage recovery and regeneration. These findings highlight the potential of bionic highly oriented hydrogels for various applications in cartilage TE.

### 4.2. Highly Oriented Hydrogels for Bone Tissue Engineering Applications

Bone is a widely distributed, large, and structurally complex mineralized connective tissue that serves crucial roles in providing mechanical support, protecting the body, and facilitating essential metabolic processes including mineral transfer and hematopoiesis 313. Bone primarily comprises organic matter, minerals, and water organized in specific structural arrangements. It consists of approximately 65% minerals by weight, mainly carbonate apatite, and 20-25% type I collagen, which serves as the predominant organic component 314. Notably, the hierarchical composite structure of bone encompasses highly oriented, interlaced cross-linked collagen protofibrils embedded within plate-like hydroxyapatite (HAp) nanocrystals. This composite structure spans multiple scales, ranging from the nano to macroscopic level, and contributes to the remarkable mechanical properties and diverse biological functions exhibited by bone 10, 315.

**Figure 16:**
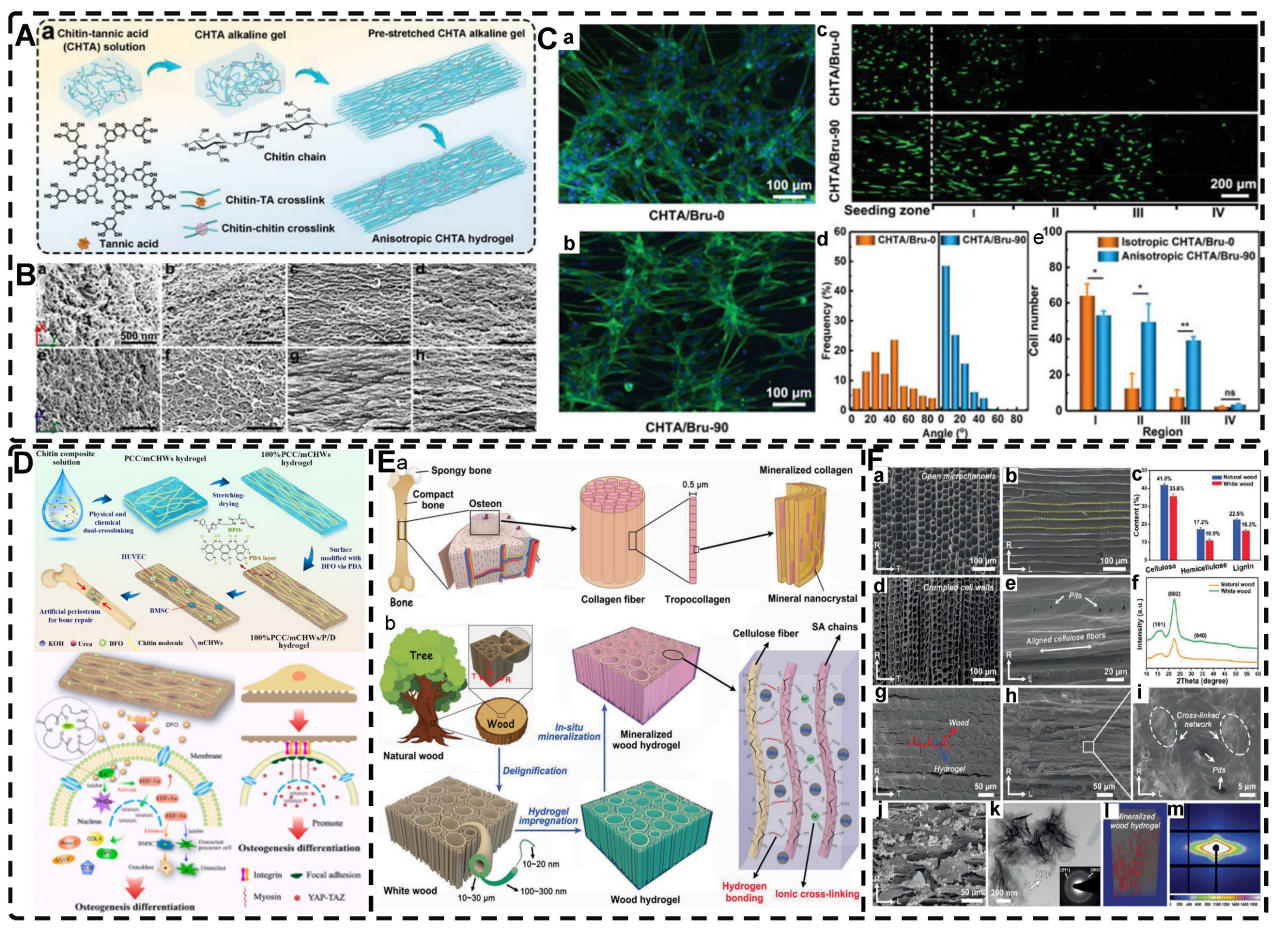
A) Processing principles of the highly oriented chitin-tannic acid hydrogel. B) Field emission SEM images of a,e) CHTA-0; b,f) CHTA-30; c,g) CHTA-60; and d,h) CHTA-90 hydrogels. C) a-c) Fluorescence microscopy images of BMSCs on isotropic CHTA/Bru-0, oriented CHTA/Bru-90 hydrogels and their migration. d, e) Angle distribution histograms and the number of the BMSCs cultured on isotropic CHTA/Bru-0 and anisotropic CHTA/Bru-90 hydrogels. Adapted with permission from 321, copyright 2022, Wiley‐VCH. D) The flowchart of the experimental process, and the mechanism diagram of DFO promoting angiogenesis. Adapted with permission from 322, copyright 2022, Elsevier. E) a) Schematic of the hierarchical structure of natural bone. b) Schematic illustration of the fabrication approach and the structure of the MWH. F) a-m) Structural and compositional characterization of highly oriented MWH. Adapted with permission from 33, copyright 2021, Wiley-VCH.

While bone possesses remarkable inherent self-healing abilities, the challenge of repairing extensive bone defects persists in the absence of external interventions 316, 317. Given the high toughness and stiffness of bone tissue, bone scaffolds represent the preferred option for filling and providing support to bone defect sites. Despite hydrogels not being the primary choice for bone TE, recent studies utilizing highly oriented hydrogels in this field have demonstrated promising applications, leading to a growing interest in bone TE 318-320. Lin et al. 321 employed a non-covalent assembly approach combined with mechanical deformation to produce highly oriented hydrogels composed of chitin and 2D materials such as molybdenum disulfide and brushite **(Figure 16A-B)**. Furthermore, apart from excellent cytocompatibility, the oriented structure's higher modulus led to superior osteogenic differentiation of BMSCs compared to isotropic hydrogels. This was supported by the increased YAP nuclear/cytoplasmic (n/c) ratios, enhanced expression of phosphorylated myosin light chains (pMLCs), upregulated expression of osteoblast-specific transcription factor runt-related transcription factor 2 (RUNX2), significant elevation in alkaline phosphatase (ALP) expression, and enhanced matrix mineralization. Immunofluorescence imaging revealed that BMSCs on the oriented hydrogel exhibited diffused and aligned distribution in the direction of orientation, indicating the guidance provided by the oriented nanofiber structure in the adhesion and migration of BMSCs** (Figure 16C)**. In vivo experiments showcased that the oriented hydrogel facilitated the migration of BMSCs towards the defect area in the rat skull, promoted osteogenic differentiation, and stimulated bone regeneration. Over time, the new bone progressively replaced the hydrogel, resulting in the simultaneous integrated lamellar bone formation and hydrogel degradation. He et al. 322 developed chitin composite highly oriented hydrogels comprising chitin chains and maleated chitin whiskers (mCHWs) using a dual chemical and physical crosslinking strategy along with a stretch-drying process. Moreover, they employed bioactive desferrioxamine (DFO) functionalization to augment the osteogenic and angiogenic properties of the oriented hydrogels. The release of DFO from hydrogels can chelate with iron ions in cells, and the activity of prolyl hydroxylases (PHDs) and the degradation of hypoxia inducible factor-1α (HIF-1α) by PHDs are inhibited without the assistance of iron ions. Upon reaching a certain threshold, the elevated expression of HIF-1α facilitated the upregulation of vascular endothelial growth factor (VEGF) as well as osteogenic differentiation-related genes (RUNX-2, OCN, OPN, etc.) **(Figure 16D)**. Consequently, this promoted the differentiation of BMSCs into osteoblasts while inhibiting the differentiation of osteoclasts from osteoblastic precursor cells. The highly oriented hydrogel structure facilitates cell adhesion, orientation, and elongation. Combined with its osteogenic and angiogenic effects, this hydrogel holds significant potential for applications in artificial bone membranes.

Although these findings that highly oriented hydrogels promote osteogenic differentiation and regeneration are exciting, the applications of highly oriented hydrogels in bone tissue engineering still has some shortcomings, such as insufficient mechanical properties and too single biological function. Therefore, to enhance the mechanical properties, such as toughness and strength, of hydrogels, Wang et al. 33 drew inspiration from wood and developed highly oriented composite hydrogels **(Figure 16E-F)**. They achieved this by impregnating sodium alginate hydrogels into delignified wood, followed by in situ mineralization of HAp nanocrystals. The in situ mineralized HAp nanocrystals promoted MC3T3-E1 cell adhesion, spreading, and alignment with cellulose protofibrils, facilitated osteogenic differentiation, and further stimulated bone formation in a rabbit femoral defect model. This composite hydrogel combines an oriented structure, excellent mechanical properties, high water absorption, and osteoconductivity, offering a promising approach for bone tissue repair. Similarly inspired by wood, Chen et al. 323 infused the delignified wood (white wood, WW) with a porous and highly oriented cellulose fiber skeleton. This scaffold was combined with a PVA hydrogel loaded with curcumin (Cur) and phytic acid (PA) to create a composite hydrogel with a highly oriented structure. The inclusion of Cur and PA in the hydrogel resulted in a synergistic increase in ALP activity and calcium nodule formation in BMSCs. Moreover, it upregulated the expression of OPN, Runx-2, and BMP-2, thereby promoting osteogenic differentiation of BMSCs. The composite oriented hydrogel exhibited a synergistic effect by inhibiting bacterial growth and inflammatory response, promoting osteogenic differentiation, and holds the potential to serve as an artificial bone membrane. As the preparation strategy for highly oriented hydrogels continues to mature, there is a growing belief that the utilization of hydrogels in bone TE will expand in the future.

### 4.3. Highly Oriented Hydrogels for Tendon Tissue Engineering Applications

Tendons are connective tissues characterized by a high degree of orientation, primarily consisting of collagen and elastin embedded within an extracellular matrix that includes water and proteoglycans, among other components. Tendons play a crucial role in linking skeletal muscle to bone, enabling the transmission of forces generated by muscle contraction for movement and postural maintenance. This unique function and their remarkable mechanical properties, characterized by high tensile strength and resistance to compressive forces, are intricately linked to their structural composition 324. Tendons possess a hierarchical orientation, wherein highly aligned collagen fibers are enveloped by membranes or sheaths of varying structures, resulting in the formation of microfibrils, subfibrils, fibers, bundles, and ultimately multiple bundles arranged in a highly ordered manner, constituting the tendon 325, 326. The ECM of tendons, along with the layered membranes or sheaths, facilitates the frictionless sliding of these highly oriented fibers, imparting them with distinctive mechanical properties. It is worth noting that the highly oriented structure of tendons, in conjunction with the ECM and the tendon cell population, achieves distinctive mechanical properties, along with viscoelastic and nonlinear elasticity beyond anisotropy 327. However, these aspects are not extensively discussed within the scope of this paper.

Tendon injuries, known as tendinopathies, significantly impact the lives, occupational activities, and overall well-being of athletes, active individuals, and older adults worldwide. Common examples include rotator cuff injuries and Achilles tendon injuries 328. Due to the limited cellularity, vascularity, and metabolic activity of tendon tissue, the recovery process after injury is notably inadequate, often resulting in matrix disorders, scar formation, and compromised mechanical properties. Restoring the mechanical properties of the original tendon after healing the damaged tissue is challenging due to two primary reasons: the regenerated tendon may lack native tendon cells, and the composition or arrangement of the ECM differs from that of the natural tendon. Consequently, these factors can result in weakness, pain, adhesions, retears, or even complete tendon rupture 324, 328, 329. The current conservative and surgical treatments available in clinical practice are limited in their effectiveness 330, 331. TE presents a promising avenue for achieving comprehensive and effective treatment of tendon injuries, with the incorporation of highly oriented structures providing improved mimicking of native tendon tissue. An illustrative example is the ANF/PVA highly oriented hydrogel developed by Sun et al. 184, which features bundled and curled highly oriented fibers that closely resemble the natural tendon's structure. Notably, when subjected to 80% elongation during stretching, the hydrogel demonstrated an impressive elastic modulus of 1.1 GPa and a strength of 72.1 MPa, effectively matching those of natural tendons. Kim et al. 177 successfully developed alginate and polyacrylamide double-network (DN) hydrogels incorporated with silica microrod inorganic fillers. These hydrogels exhibited excellent mechanical properties and featured a highly oriented anisotropic structure reminiscent of natural tendons. The hydrogel holds promising potential for application in artificial tendon engineering. However, a comprehensive evaluation of the composite's biological behavior necessitates cell encapsulation.

**Figure 17:**
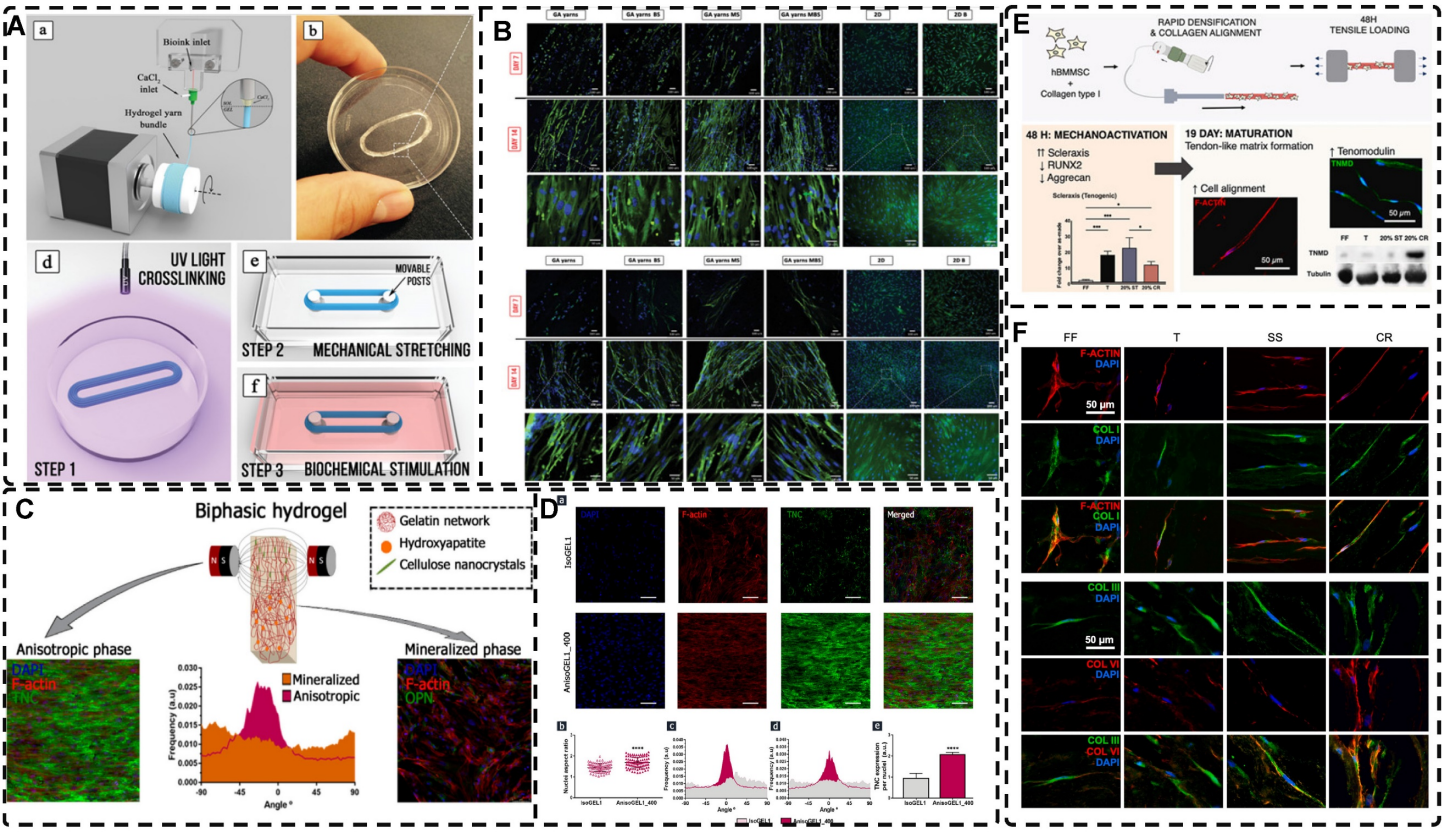
A) a-f) Schematic of biofabrication and stimulation of 3D oriented cell-laden hydrogel yarns using the wet-spinning technique. B). Collagen I expressed by hBM-MSCs encapsulated into different hydrogels. Adapted with permission from 332, copyright 2019, Wiley-VCH. C) Schematic of biofabrication of cellulose nanocrystals (CNC) and hydroxyapatite (HA) oriented and aligned under a magnetic field. D) a-f) The nuclei aspect ratio, directionality of cell distribution, and tendon-related ECM protein TNC secretion were assessed by confocal immunofluorescence. Adapted with permission from 31, copyright 2019, American Chemical Society. E) Schematic of biofabrication of highly oriented ADC hydrogel scaffolds and the ability to tenogenic differentiation. F) Cellular morphology and collagen formation of hBM-MSC-seeded ADCs of 20% static strain (SS) and 20% cyclic rest (CR) and their controls, free-floating (FF) and tethered (T). Adapted with permission from 190, copyright 2022, Elsevier.

Furthermore, highly oriented structures, such as electrostatically spun nanomicrofiber scaffolds, can serve as valuable cues for directing cell differentiation towards tendon formation 27. However, these scaffolds are deficient in an ECM-like 3D environment, which can be effectively supplemented by hydrogels. Liu et al. 30 employed Flight biomanufacturing technology to prepare highly oriented microfilament hydrogels, which facilitated the oriented alignment of human tenocytes (HTs). After two weeks of co-culture with the highly oriented hydrogel, HTs exhibited a remarkable presence of highly aligned filamentous actin (F-actin) **(Figure 13C)**. Furthermore, immunofluorescence analysis revealed that the highly oriented microfilaments effectively guided the oriented deposition of cells and ECM, as evidenced by the expression of type I collagen. Rinoldi et al. 332 successfully fabricated 3D oriented cell-laden hydrogel yarns using the wet-spinning technique, which closely mimic the structural characteristics of natural tendon bundles** (Figure 17A)**. The highly oriented structure, combined with mechanical stimulation through stretch and biochemical stimulation via the growth factor BMP-12, effectively facilitated hBM-MSC attachment, spreading, elongation, and oriented alignment, resulting in the formation of dense and highly oriented 3D cell constructs **(Figure 17B)**. Moreover, this approach holds the potential to promote collagen orientation and deposition, ultimately leading to the development of an oriented matrix. Furthermore, the observed significant expression of tendon-related genes, such as scleraxis (SCX), tenomodulin (TNMD), collagen I, and collagen III, strongly suggested the successful tengenic differentiation of hBM-MSCs, thus highlighting the potential of this method for tendon TE. Park et al. 190 employed the gel GAE method to efficiently densify and remodel collagen, yielding a highly oriented aligned dense collagen (ADC) system **(Figure 17E)**. The casting solution removal enhances collagen fiber density (CFD) or collagen content, while the applied pressure difference facilitates the orientation and alignment of the resulting hydrogel. Short-term, high-strain mechanical stimulation of highly oriented ADC hydrogels significantly enhanced the expression of tendon-related genes, including Tenascin-C and tenomodulin **(Figure 17F)**. This stimulation led to substantial remodeling of the matrix, resulting in the formation of a mature tendon-like matrix. These findings exemplify the significant potential for employing highly oriented ADC hydrogels in conjunction with mechanical stimulation for tendon TE.

Tendon-to-bone integration, also known as the point of attachment called enthesis, is another characteristic of tendons. The enthesis comprises four distinct zones with remarkable adhesive and mechanical properties: pure dense fibrous connective tissue, uncalcified fibrocartilage, calcified fibrocartilage, and bone 333, 334. Currently, TE approaches for the tendon-bone interface predominantly focus on fibrous scaffold development 335-338. Kim et al. 180 developed a triple-network hydrogel comprising imidazole-containing polyaspartamide and an energy-dissipative alginate-polyacrylamide double-network. This hydrogel demonstrated an exceptional tensile modulus (3.0 MPa) and strength (0.8 MPa), as well as strong adhesion to bone (2.73 kJ m^-2^ and 725 kPa), effectively mimicking the native tendon-to-bone attachment interface. Furthermore, the hydrogel's excellent biocompatibility suggests its considerable potential for advancing TE research and fostering future developments in the field. Hierarchically highly oriented hydrogels were prepared by Echave et al. 31 through the incorporation of CNCs and HAp into gelatin networks that were oriented and aligned under a magnetic field **(Figure 17C)**. During the tendon-mimicking phase, hASCs encapsulated within highly oriented hydrogels exhibited a spindle-shaped morphology characterized by elongated nucleus length diameter, aligned cytoskeleton, and enhanced expression of the tendon-associated ECM protein TNC **(Figure 17D)**. These observations indicate the potential of the hydrogels to effectively mimic tendon tissue when compared to the isotropic system. During the bone-like phase, the inclusion of HA particles in the hydrogel enhanced its rigidity and promoted the differentiation of hASCs toward the osteogenic lineage. This differentiation process resulted in an upregulation of bone markers, including the bone bridge protein OPN. The hierarchical structure of these hydrogels closely mimicked the composition, structure, and cellular organization found in natural tendon-bone attachment sites, thereby offering significant potential for various applications.

### 4.4. Highly Oriented Hydrogels for Skeletal Muscle Tissue Engineering Applications

Skeletal muscle constitutes approximately 45% of the total body weight and serves crucial functions such as protecting and supporting internal organs, facilitating body movement, and regulating body temperature and basal energy metabolism 339. Skeletal muscle comprises numerous myofibrils, the fundamental functional units of muscle fibers. These myofibrils are arranged in series, giving rise to highly oriented myogenic fibers that are tightly packed within the endomysium. Subsequently, these myofibrils are enveloped by fascicles, which form bundles, and ultimately, the bundles are encased by the epimysium, thereby forming the muscle tissue 340, 341. The oriented structure of skeletal muscle contributes to its notable mechanical strength along the direction of fiber accumulation and high elasticity coefficient in the direction of alignment, thereby ensuring stability during a wide range of postures and movements 38, 40.

**Figure 18:**
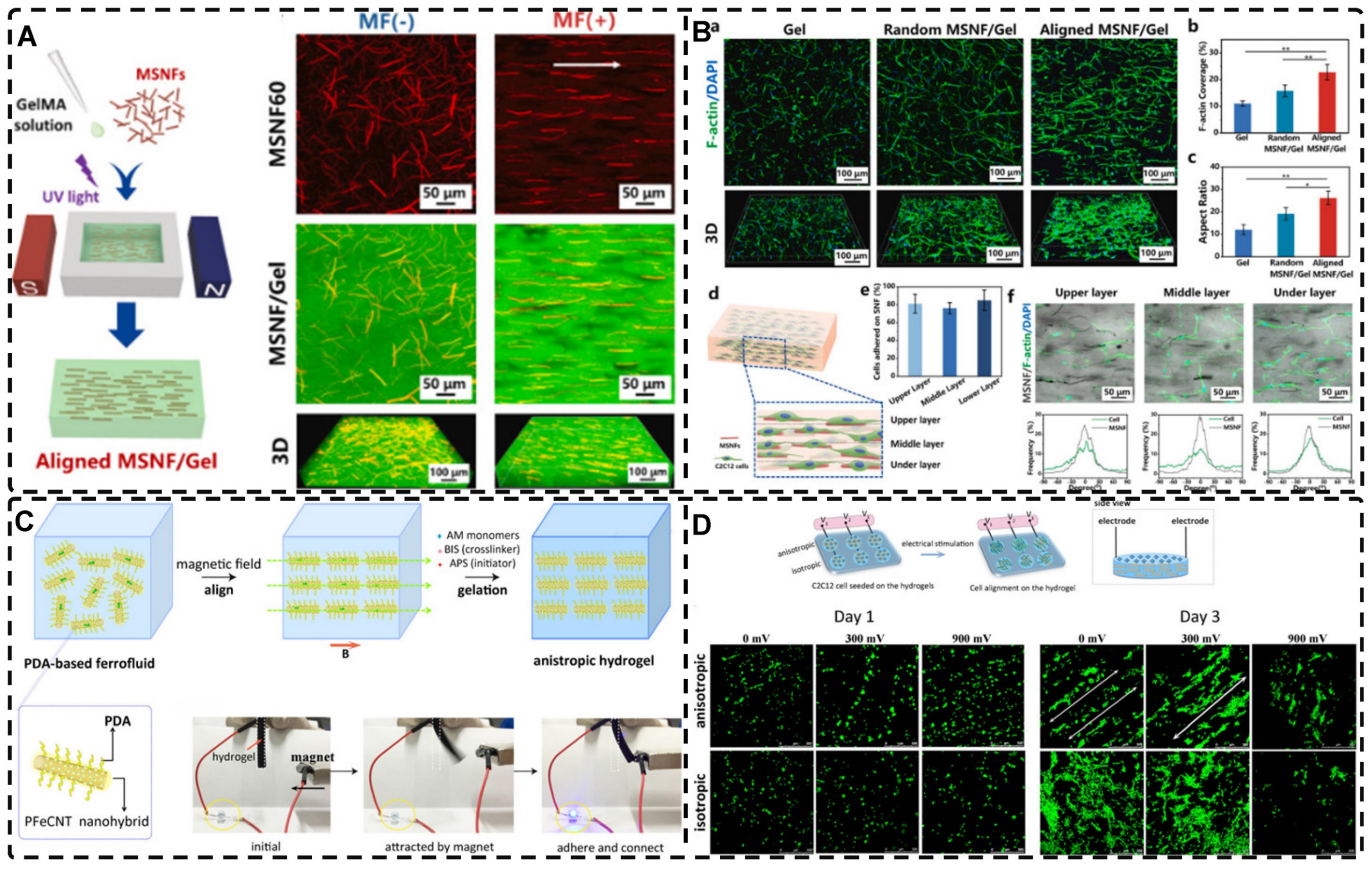
A) Schematic illustration of the fabrication of aligned MSNF/Gel scaffold in situ under magnetic field, and the fluorescence images showed that MSNF was oriented encapsulated within GelMA hydrogels. B) (a-c) Cytoskeleton morphology of C2C12 cells encapsulated within Gel, random MSNF/Gel scaffold and oriented MSNF/Gel scaffold after culturing for 3 days, and their F-actin coverage and aspect ratio. (d-f) C2C12 cells adhered to MSNFs and showed oriented aligned morphology in each layer within the aligned MSNF/Gel scaffold. Adapted with permission from 29, copyright 2022, Elsevier. C) Schematics of preparation of an anisotropic hydrogel based on a mussel-inspired conductive ferrofluid. D) Schematic representation of C2C12 cells seeded on the hydrogels and cells being electrically stimulated under different voltages, and CLSM micrographs of C2C12 cells on the anisotropic and isotropic hydrogels after 1 and 3 days of culturing under different electrical stimulation voltages. Adapted with permission from 85, copyright 2019, American Chemical Society.

Skeletal muscle possesses intrinsic regenerative capabilities to repair minor injuries. However, its capacity to heal is hindered when the damage or loss exceeds a critical threshold, resulting in scar tissue formation and functional impairments, such as volumetric muscle loss following trauma or surgical interventions 342-344. Surgical transplantation of autologous healthy muscle tissue/flaps is a commonly employed strategy to mitigate scar tissue formation and preserve functionality. However, this approach is associated with limitations, including inadequate availability of donor tissue, compromised donor function, and heightened morbidity at the donor site 341, 345. Muscle TE has emerged as a means to replace damaged or absent muscle tissue, aiming to facilitate tissue regeneration and restore functional capacity in areas affected by muscle deficiency. Bionic materials with high orientation offer a promising approach as they guide the alignment of muscle cells and facilitate myoblast regeneration, resulting in the formation of densely packed muscle fibers that align closely with aesthetic requirements 40, 346. Additionally, these materials promote tissue regeneration and functional recovery. Highly oriented hydrogels play a crucial role in muscle TE and have garnered considerable attention in current research endeavors. A graphene-polysaccharide nanocomposite hydrogel film, meticulously designed by Patel et al. 170, exhibited a highly oriented structure and elicited an initial alignment of mouse myoblast cells (C2C12) upon inoculation. Furthermore, the hydrogel promotes the differentiation of these cells into multinucleated myotubes. Highly oriented MSNF/GelMA hydrogels, as developed by Wang et al. 29, demonstrated superior capability in promoting the elongation and alignment of C2C12 cells when compared to random MSNF/GelMA and pure GelMA hydrogels **(Figure 4H, Figure 18A)**. This effect can be attributed to the affinity of the cells for the MSNF, leading to their alignment and elongation along the highly oriented structure of the hydrogel **(Figure 18B)**. Furthermore, mice treated with the highly oriented MSNF/GelMA hydrogel exhibited an increased number of longer myotubes formed by fused C2C12 cells compared to mice in other groups. This observation highlighted the capacity of the highly oriented MSNF structure within the hydrogel to enhance the three-dimensional myogenic differentiation of C2C12 cells. Additionally, mouse satellite cells encapsulated within the highly oriented hydrogel group demonstrated spontaneous beating and synchronous contraction, aligning with the highly oriented direction of the MSNF. This observation further supports the notion that highly oriented structural hydrogels hold significant promise as a TE strategy for skeletal muscle regeneration. Liu et al. prepared highly oriented microfilament hydrogels using Flight biomanufacturing technology, in which C2C12 myofibroblasts were encapsulated. The fusion of myofibroblasts and alignment of myotubes in these hydrogels were observed through immunofluorescence staining, using anti-mouse myosin heavy chain (MyHC) antibody and ghost pen cyclic peptide **(Figure 13C)** 30. The highly oriented microfilament hydrogels exhibited a remarkable contrast to bulk hydrogels, with 95% of myotubes aligning parallel to the microfilaments, and over 76% of myotubes containing more than six nuclei per myotube, a feature absent in bulk hydrogels. These findings highlight the potential of highly oriented microfilament hydrogels in muscle TE and their role in advancing the biofabrication of muscle tissue-engineered constructs. Highly oriented aligned hydrogels containing polydopamine-Fe_3_O_4_-CNTs were prepared by Liu et al. 85 to mimic muscle tissue **(Figure 18C)**. These highly oriented hydrogels exhibited superior orientation conductivity and mechanical properties compared to randomly aligned hydrogels, and when combined with oriented electrical stimulation, they promoted the directed growth and elongation of C2C12 mouse myogenic cells, as evidenced by immunofluorescent staining of the cell adhesion protein Vinculin (green) **(Figure 18D)**. Furthermore, the highly oriented hydrogel, when subjected to electrical stimulation, exhibited cortical and homogeneous cytosolic staining of oriented vinculin, along with ellipsoidal cell nuclei and the formation of clustered cell protrusions at the cell periphery. These observations confirmed that the highly oriented hydrogel, in synergy with electrical stimulation, effectively guided the directional growth of C2C12 cells. These studies provide compelling evidence for the potential applications of highly oriented hydrogels in skeletal muscle TE.

### 4.5. Highly Oriented Hydrogels for Cardiac Muscle Tissue Engineering Applications

Cardiovascular disease is the leading cause of global mortality, resulting in approximately 18.6 million deaths in 2019, marking a 17.1% increase since 2010 347. Notably, myocardial infarction or ischemic heart disease stands out as the primary contributor to the elevated morbidity and mortality associated with cardiovascular diseases. During this pathological process, there is a significant reduction in blood supply to the infarcted area, leading to the demise of myocardial cells. Presently, available therapeutic and preventive strategies encompass pharmaceutical interventions involving catecholamines, angiotensin, aldosterone, surgical interventions, and the implantation of pacemaker devices. However, given their limited effectiveness in halting disease progression, heart transplantation stands as the definitive treatment option 348. In light of the challenges posed by immunosuppression and the scarcity of cardiac donors, the development of artificial cardiac tissue-engineered hydrogels, stents, and patches has gained substantial attention over the last twenty years 349, 350.

**Figure 19:**
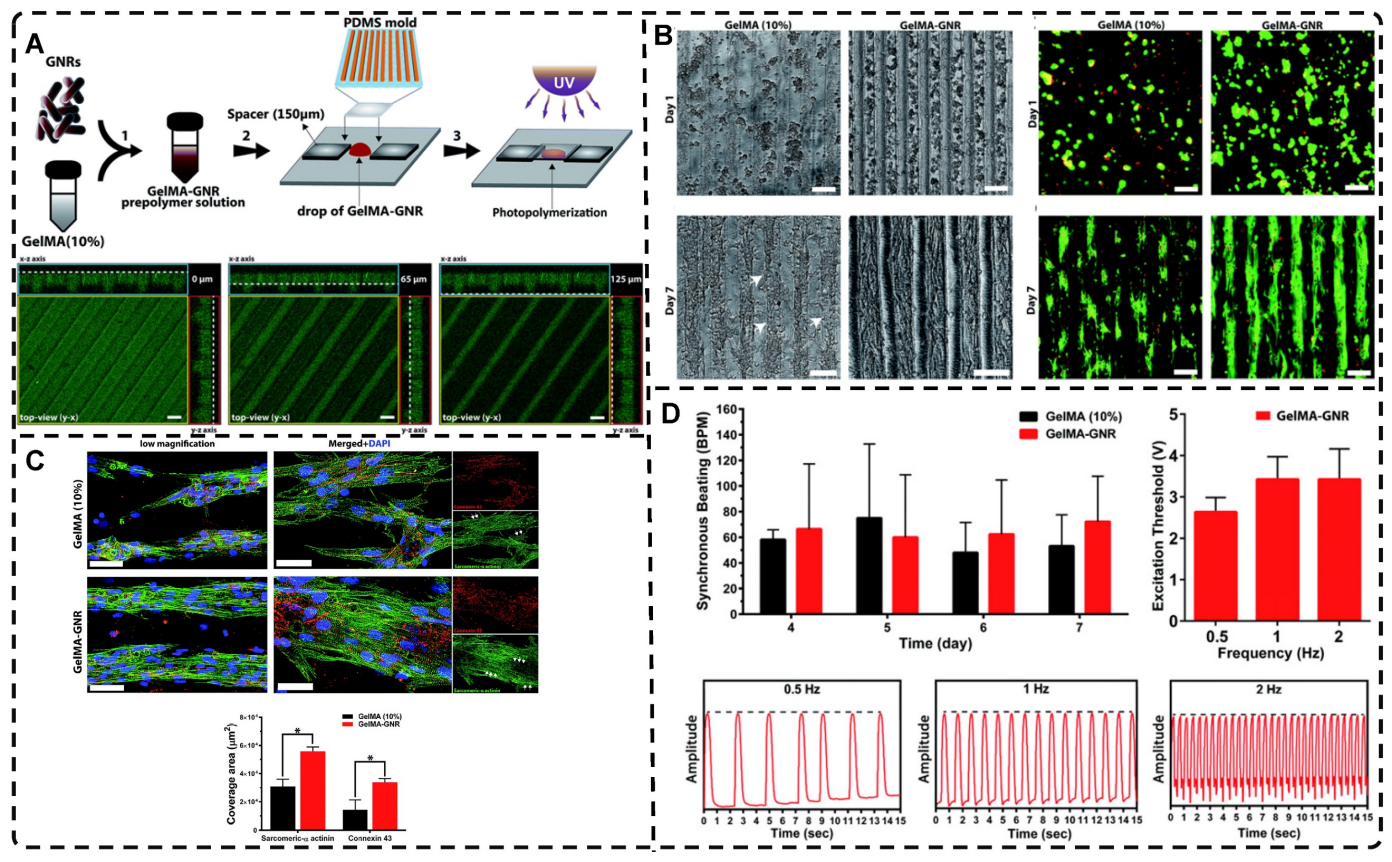
A) Schematic illustration of the fabrication procedure of GelMA-GNR microgrooved tissues, and z-Stack images showing the 3D structure of the microgrooved patterns. B) Phase-contrast images of cardiac cells seeded on GelMA and GelMA-GNR microgrooved hydrogel, and fluorescent viability images of GelMA and GelMA-GNR microgrooved cardiac tissues on day 1 and day 7 of culture. C) Immunostained images of SATN (green) and Cx43 gap junctions (red) within GelMA and GelMA-GNR microgrooved cardiac tissues on day 7 of culture, and quantified area coverage of cardiac specific markers on day 7. D) Synchronous spontaneous beating behavior (beats per minute; BPM) of cardiac tissues on GelMA and GelMA-GNR constructs, voltage excitation thresholds, and extracted beating signals of GelMA-GNR cardiac tissue at different frequencies. Adapted with permission from 356, copyright 2017, Royal Society of Chemistry.

The cardiac muscle is composed of numerous highly oriented cardiomyocytes, each containing an abundance of myofibrils aligned parallel to the long axis of the cells 13. Consequently, the development of cardiac TE necessitates highly oriented manufacturing strategies capable of emulating the natural myocardial structure and facilitating the elongated morphology and functionality of the cells 351-355. Wang et al. 153 fabricated highly oriented PPy nanocomposite hydrogels through mechanical stretching, inducing a cardiomyocyte alignment resembling that of the natural myocardium, along with an elongated cell morphology **(Figure 6D)**. The hydrogels exhibited excellent electrical conductivity and enabled stable and directional transmission of electrical signals, potentially reducing the occurrence of myocardial infarction. Ye et al. 35 developed nanoscale highly oriented cellulose hydrogels through pre-stretching and leveraging of cellulose's inherent robust self-aggregation forces within an alkali/urea aqueous solution **(Figure 11C)**. The subsequent acid treatment and release of the pre-stretched highly oriented hydrogels in a dilute acid solution resulted in the formation of microgroove-like structures that promoted the alignment and adhesion of neonatal rat ventricular myocytes (NRVM) along the orientation of the stretch force. Furthermore, the cultured NRVM exhibited enhanced mitochondrial content, well-defined myonodular structures, and spontaneous beating. Navaei et al. 356 synthesized gold nanorods (GNRs) and GelMA hydrogel with surface-concave microgrooves with a width and depth of 50 μm, using PDMS molds to emulate the electrical functionality and histomorphology of natural cardiac muscle **(Figure 19A)**. Compared with pure GelMA hydrogels, the F-Actin-stained images and data on the area of fluorescence demonstrated the development of uniformly dense and highly aligned cardiac tissue in GelMA-GNR hydrogels **(Figure 19B-C)**. Neonatal rat cardiomyocytes cultured on both hydrogels for 4-7 days displayed spontaneous contractility. However, only the cardiac tissue formed on the electrically conductive GelMA-GNR hydrogels exhibited responsiveness to external stimuli at various frequencies (0.5, 1, and 2 Hz) at low voltages **(Figure 19D)**. These findings highlight the potential of the microgroove GelMA-GNR hydrogel for constructing natural cardiac tissue with the capability to function as a cardiac patch after myocardial infarction.

The establishment of vascularized myocardial tissue is important for the exchange of nutrients and oxygen with cardiomyocytes, and poses a significant challenge for TE. Mehrotrad et al. 357 presented a novel silk-based biomaterial ink for fabricating well-aligned cardiac structures with favorable elasticity and stiffness (≈40 kPa). The anisotropic hydrogel scaffold, fabricated using the double cross-linking method, facilitated the organization of aligned sarcomeres, promoted the upregulation of gap junction proteins like connexin-43, and sustained the coordinated contraction of cardiomyocytes. Furthermore, they endeavored to generate vascularized myocardial tissue by encapsulating human, induced pluripotent stem cell-derived cardiac spheroids (hiPSC-CSs) in silk-based inks for viability. They employed embedded bioprinting techniques to create perfusable and well-aligned vascular channels. The endothelial cells enclosed within the bioink of these aligned channels could gradually migrate toward the channel edges, establishing an endothelial vascular network. As the culture matured, the vascularized myocardial tissue exhibited maturity-dependent protein expression in both cardiomyocytes and human umbilical vein endothelial cells (HUVECs). Establishing vascularized in vitro tissues is an important step in advancing TE, and this approach warrants continued investigation and refinement.

The above study was performed on a single directionally oriented hydrogel applied to cardiac TE, whereas the actual native cardiac tissue showed a multilayered, oriented arrangement and a gradual transition in the orientation of the arrangement between the layers of the myocardium 358, 359. Therefore, a design of highly oriented hydrogels with distinct orientations between layers would offer greater potential for mimicking the structure of natural myocardial tissue. Wu et al. 246 prepared a GelMA hydrogel incorporating a highly oriented NFYs-NET structure, effectively emulating the architecture of the native heart **(Figure 14A)**. The presence of NFYs-NET within the hydrogel scaffold facilitated cellular alignment, elongation, and promoted the maturation and functionality of CMs **(Figure 14B-C)**. Simultaneously, the hydrogel provided a three-dimensional environment that supported nutrient exchange and offered mechanical protection. The incorporation of layered NFYs-NET structures within the GelMA hydrogel enabled the creation of a biomimetic multilayer orientation akin to the native heart **(Figure 14D)**; each layer's orientation independently influenced the arrangement of cells, closely resembling the multiple cell layers found in natural cardiac tissue. Moreover, a hybridization approach was employed to construct endothelialized myocardium by co-culturing cardiomyocytes on the NFYs-NET layer and encapsulating endothelial cells within hydrogels **(Figure 14E)**. These findings underscore the significant potential for developing and applying this three-dimensional, highly oriented hydrogel scaffold in cardiac TE.

### 4.6. Highly Oriented Hydrogels for Nerve Tissue Engineering Applications

Nervous tissue plays a crucial role in transmitting information and signals within the body. It is divided into two main components: the central nervous system (CNS) and the peripheral nervous system (PNS) 360. The PNS facilitates the transmission of information and signals between the CNS and the body via its extensive network of neurons, whereas the CNS processes and coordinates these signals 361. Neurons, which are the fundamental structural and functional units of the nervous system, consist of a cell body, highly branched dendrites that extend from the cell body, and a single axon per neuron. Dendrites receive impulses from the axons of other neurons and relay them to the cell body, which in turn transmits these impulses in a unidirectional manner through its axon to other neurons, ensuring the directionality of nerve impulses 362. Anatomically, nerve tissue exhibits a hierarchical organization, with nerve fibers arranged and oriented to form fiber bundles. Nerve fibers possess distinctive features, such as a nano-oriented fibrous ECM and a core-shell structure composed of a tubular myelin sheath that envelops elongated axons 14. These structural attributes contribute to the pronounced orientation observed in neural organization.

#### 4.6.1. Peripheral Nerve

**Figure 20:**
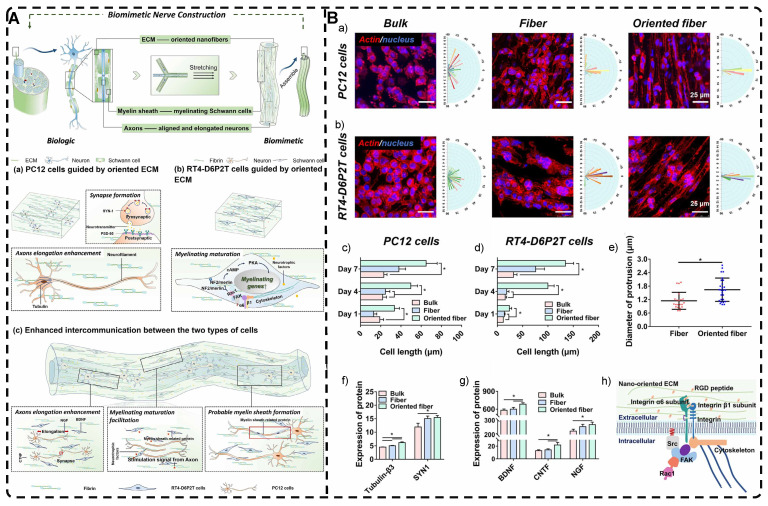
A) Schematic illustration of constructing biomimetic nerve fibers and their neural performances. (a) PC12 cells guided by oriented ECM; (b) RT4-D6P2T cells guided by oriented ECM; (c) Enhanced intercommunication between the two types of cells. B) (a, b) Morphology of PC12 and RT4-D6P2T cells cultured for 7 d. (c, d) Histograms of PC12 and RT4-D6P2T cells length. (e) Axons diameters of PC12 cells counted from immunofluorescence images marked by Tubulin-*β*3. (f, g) Elisa assays of Tubulin-*β*3 and SYN1 expressed by PC12 cells and neurotrophic factors including BDNF, CNTF and NGF expressed by RT4-D6P2T cells. (h) Schematic illustration of possible extracellular topographic signaling transduction mechanism. Adapted with permission from 14, copyright 2020, IOP Publishing.

The PNS possesses inherent regenerative capabilities but faces challenges in self-healing when the defect length exceeds 5 mm, and becomes incapable of self-healing beyond 10 mm 363-365. Short-gap injuries are typically treated with direct clinical suturing, which is considered the gold standard approach 366. Conversely, repair of long-gap peripheral nerve injuries (PNI) often requires autografts 365. Nonetheless, autografts are beset by limitations, including size mismatch, limited availability of suitable donor tissue, the need for secondary surgical procedures, and permanent damage to the donor site. Neural TE aims to facilitate nerve regeneration through the development of tissue-engineered bioactive nerve substitutes, which then serve as optimal scaffolds or channels for axonal regeneration, leading to the reconstruction of nerve circuits and restoration of tissue function 367-369. Several scaffolds have received FDA approval, substantiating the potential of these alternatives in PNI repair 370, 371. Natural neural tissue exhibits a low elastic modulus, typically below 1 kPa, and hydrogels can serve as effective mimics of this mechanical property 372. Particular attention should be given to the fabrication of biomimetic neural tissue using hydrogels as the primary framework. Incorporating highly oriented structures into these hydrogels can provide biophysical and biological cues that promote and guide the growth of neural tissues, facilitating the reconstruction of neural circuits. Babu et al. 373 developed oriented hydrogels by incorporating a low concentration of SPION (400 μg/ml) microgel into synthetic polyethylene glycol hydrogels, aligning them using low magnetic fields (≈100 mT). The hydrogel facilitated axonal growth and orientation during in vitro culture. However, the microgel's ability to direct cells in an oriented manner was compromised by the presence of local cell binding sites on the microgel. Intriguingly, coupling the microgel covalently to a matrix PEG hydrogel resulted in diminished orientation and growth of neuronal cells. This effect may be attributed to the modification of anisotropic force transmission due to covalent coupling, thereby attenuating the influence of mechanical signals that facilitate directional cell growth. Ghaderinejad et al. 374 fabricated magnetic hydrogels filled with short fibers oriented using unidirectional freeze-drying in a sodium alginate matrix. Olfactory ecto-mesenchymal stem cells (OE-MSCs) cultured in highly oriented hydrogels demonstrated significantly improved proliferation rates and a more elongated and flattened morphology compared to those cultured on pure hydrogels. Moreover, the heightened expression of mature neuronal markers β-tubulin III and Glial fibrillary acidic protein (GFAP) confirmed the neural differentiation of OE-MSCs. Lu et al. 375 fabricated micro-nano hierarchical hydrogels with aligned orientation as fillers for nerve conduits subjected to electric field treatment. Oriented aligned filled nerve conduits (ASFN), in comparison to randomly oriented filled nerve conduits (RSFN), effectively guided the orientation of Schwann cells and exhibited enhanced cell proliferation. Additionally, the axons of PC12 cells exhibited elongation and alignment along the oriented structure to a greater extent. Upon implantation in a rat sciatic nerve defect model, ASFN demonstrated comparable outcomes to autografted nerves, outperforming RSFN and unfilled nerve conduits, highlighting it as a viable alternative for bionic nerve tissue. Chen et al. 14 utilized a microfluidic system to fabricate core-shell structured biomimetic tubular hydrogels with an oriented ECM **(Figure 20A)**. The highly oriented hydrogels facilitated the elongation and oriented alignment of PC12 cells and RT4-D6P2T cells, i.e., myelinating Schwann cells, to a greater extent compared to a bulk composite hydrogel with random orientation **(Figure 20B)**. They effectively guided the orientation and extension of neurons while enhancing the elongation alignment of neurons and the maturation of myelin sheaths in Schwann cells. The core-shell structure of the biomimetic tubular hydrogel constructs, comprised of nano-oriented fiber ECM, core elongated neurons, and shell myelinating Schwann cells resembling tubular myelin sheaths, showcases the successful fabrication of bionic nerve fibers with significant potential in neuroregenerative TE.

#### 4.6.2. Spinal Cord

**Figure 21:**
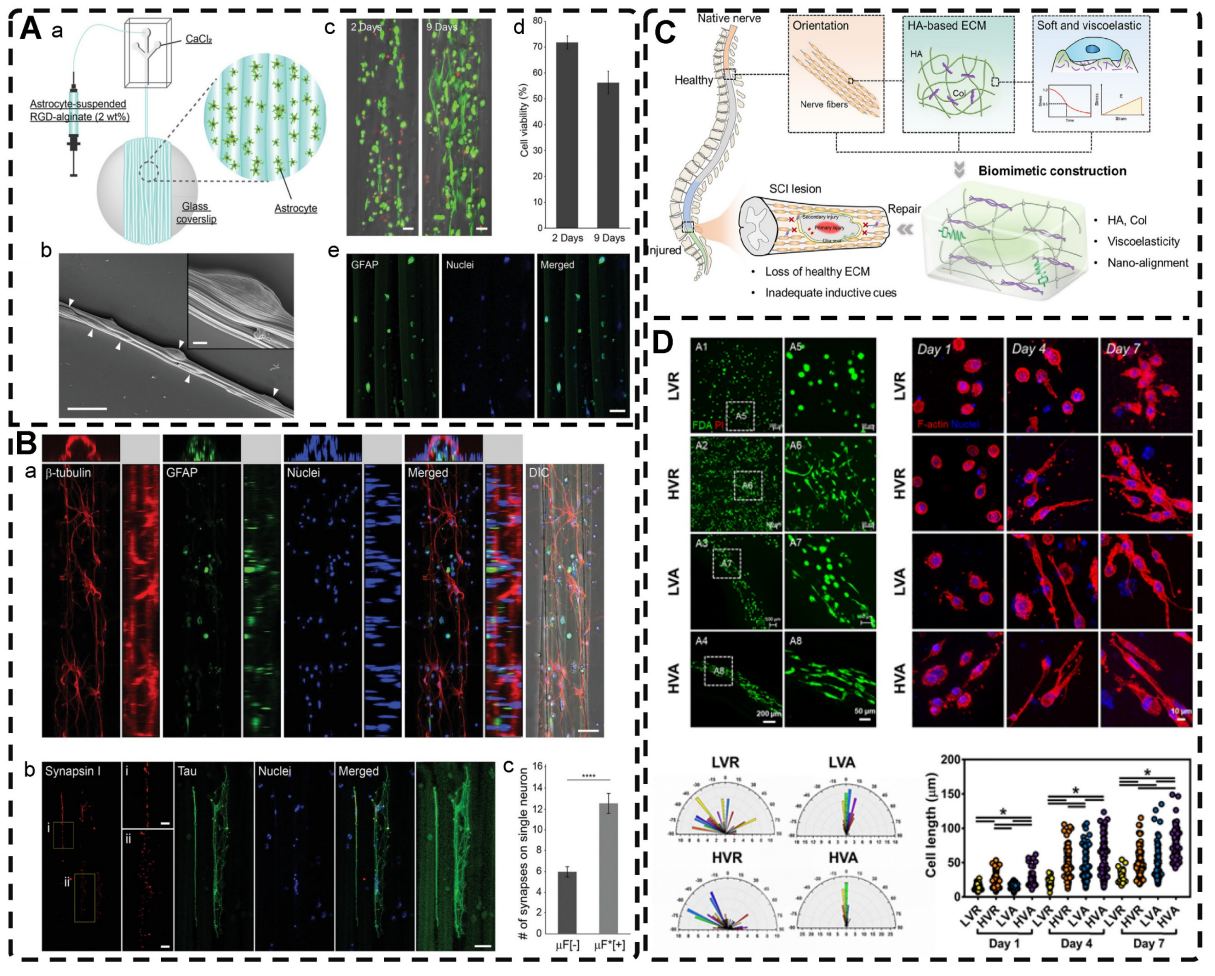
A) a) Schematic illustration for astrocyte-laden microfibers. b) SEM image of astrocyte-laden microfibers after drying and dehydrating. c) CLSM images of astrocytes encapsulated in the fibers. d) Viabilities of astrocytes encapsulated in the fibers on 2 and 9 days after fabrication. e) CLSM images of astrocytes stained for GFAP and nucleus on 9 days after fabrication. B) a, b) Maximum-intensity-projected CLSM *z*-stack images of hippocampal neurons on astrocyte-laden microfibers. c) Number of presynapses of a single hippocampal neuron. Adapted with permission from 381, copyright 2022, Wiley-VCH. C) Schematic illustration for biomimetic construction of HA/Col hydrogel incorporated with viscoelasticity and nano-alignment, as well as the exploration for co-effects of extracellular viscoelasticity and nano-orientation on neuronal cell/tissue behaviors. D) Viability and morphology of PC12 cells. Adapted with permission from 28, copyright 2021, Elsevier.

Spinal cord injury (SCI) initiates a complex, multifactorial cascade of responses, wherein primary injury, such as neuronal and glial cell death and vascular rupture, is typically succeeded by secondary injury, including secondary ischemia, edema, hypoxia, inflammatory response, and calcium overload 376. In response, the body employs inherent protective mechanisms, such as the formation of a glial scar, to prevent further harm 377. However, the glial scar ECM contains inhibitors of axonal growth, such as chondroitin sulfate and proteoglycan, which hinder neuronal regeneration and significantly impede the restoration of neurogenic function in SCI 377. Clinical interventions, including medication and decompression surgery, primarily focus on safeguarding the spinal cord against further damage, yet they yield limited gains in terms of functional recovery in the reconstructed spinal cord 378, 379. The implantation of tissue-engineered scaffolds, which can induce cellular targeting and promote spinal cord regeneration, is a burgeoning area of research, with highly oriented hydrogels assuming a crucial role 380. For instance, Kim et al. 381 utilized microfluidic technology to encapsulate astrocytes in alginate hydrogel incorporated with the tripeptide arg-gly-asp (RGD), thus producing oriented micron-scale hydrogel fibers **(Figure 21A)**. Their experimental findings indicated that hydrogel microfibers loaded with astrocytes expedite neurite outgrowth and guide neurite elongation directionality during the early stages **(Figure 21B)**. Furthermore, they facilitate synapse formation and the establishment of well-organized neural circuits during later stages of development. Despite the relatively low viability of the encapsulated astrocytes, this method holds potential for further exploration in neural TE. Chen et al. 382 developed a stretchable and adherent, highly oriented hydrogel through electrostatic spinning and in situ sequential cross-linking. This hydrogel exhibited the ability to achieve spatiotemporal delivery of stromal cell-derived factor-1α (SDF1α) and paclitaxel while being orientation-aligned with collagen fibronectin. In vitro and in vivo experiments revealed that the oriented hydrogels stimulated the migration of exogenous or endogenous neural stem/progenitor cells (NSPCs) to intermediate/lesion sites and facilitated their neuronal differentiation. Ultimately, this process led to the reconstruction of neural networks and the restoration of function. Gao et al. 383 developed a silk fibroin nanofiber hydrogel with a hierarchically oriented microstructure and incorporated nerve growth factor (NGF), which enabled the scarless repair of the spinal cord in a rat model of spinal cord hemisection. This NGF-enriched oriented hydrogel improved Schwann cell alignment and spreading, and also enhanced axonal growth and length in PC12 cells, highlighting its ability to stimulate nerve cell proliferation, alignment, spreading, and migration. When implanted in a rat spinal cord hemisection model, the hydrogel promoted migration and differentiation of neural stem cells (NSCs) and stimulated angiogenesis, leading to scar-free regeneration of the spinal cord and functional recovery comparable to that of the intact spinal cord. This approach, combining biological and physical cues for tissue regeneration, demonstrates the feasibility of utilizing multiple cues to construct bionic neural tissue. Chen et al. 28 fabricated biomimetic oriented hydrogels with nano-alignment and viscoelastic properties using a static-dynamic strategy and microfluidic techniques **(Figure 21C)**. In vitro, the oriented hydrogel effectively guided the orientation of neuronal cells (PC12), while the nano-alignment and viscoelasticity positively influenced neuronal polarization and axonal elongation **(Figure 21D)**. Moreover, the combined influence of the nano-oriented structure and viscoelasticity resulted in enhanced neural differentiation, as evidenced by a significant upregulation of neurogenic genes, while no enhancement in glial differentiation of BMSCs was observed. In a SD rat spinal cord hemisection model, the implantation of the oriented hydrogel graft promoted neuronal differentiation and facilitated functional axon regeneration, while inhibiting cystic and glial formation, ultimately, positively impacting the functional recovery following spinal cord injury. The combination of orientation and viscoelasticity in this hydrogel serves as a valuable reference for the development of neural grafts with multiple guidance cues, significantly contributing to the field of neural TE.

#### 4.6.3. Brain

The brain is the most intricate and sophisticated component of the nervous system, and brain injuries often result in enduring neurological impairments 384, 385 due to the limited regenerative capacity of brain tissue and the lack of sufficient nerve growth factors 386. While numerous therapeutic approaches have been investigated in laboratory settings, current clinical treatments struggle to rebuild brain structures and restore their functions effectively 387. The potential of aligned oriented in TE for brain injury repair has received limited attention, possibly due to the subtle nature of structural alignments in the brain at the tissue level. At the cellular level, cortical tissue does exhibit spatially oriented aligned structures, such as neurons with elongated axons capable of unidirectional pulse signaling and radial glial (RG) cells that extend aligned fibers from the soma to the meninges 388, 389. Hence, the utilization of highly oriented hydrogels that replicate the structural orientation found in natural tissues holds significant value in brain TE investigations. Chai et al. 390 implanted electrostatically spun and molecularly self-assembled highly oriented fibronectin hydrogels into the subventricular zones (SVZ) to facilitate the migration of endogenous NSCs towards the lesioned area, aiming to replicate the orientation and functionality of natural RG fibers. In vitro experiments demonstrated that the cytoskeleton of NSCs elongated along the long axis of the oriented hydrogel, resulting in oriented and extended axons. When implanted into a rat brain injury model, this hydrogel led to the development of regenerated tissue with an oriented morphology, unlike the results with randomly oriented hydrogel. Additionally, the implantation facilitated the differentiation and maturation of NSCs and the recovery of neurological function following brain injury in rats. RNA transcriptome analysis conducted on regenerated tissues 14 days after the operation revealed upregulation of neurogenic transcripts, including Sema3A and Sema6A, which are linked to neuronal migration, as well as Plexin2, Plxna4, and Plxnb4, associated with axonogenesis. Moreover, the oriented hydrogel facilitates neural regeneration by engaging multiple signaling pathways, including those of Notch, Wnt, axon guidance, MAPK, and neurotrophin. This study highlights the clinical translation potential of the oriented hydrogel in brain TE, while also offering novel insights for future investigations into highly oriented hydrogels for the repair of brain injuries.

### 4.7. Highly Oriented Hydrogels for Corneal Tissue Engineering Applications

**Figure 22:**
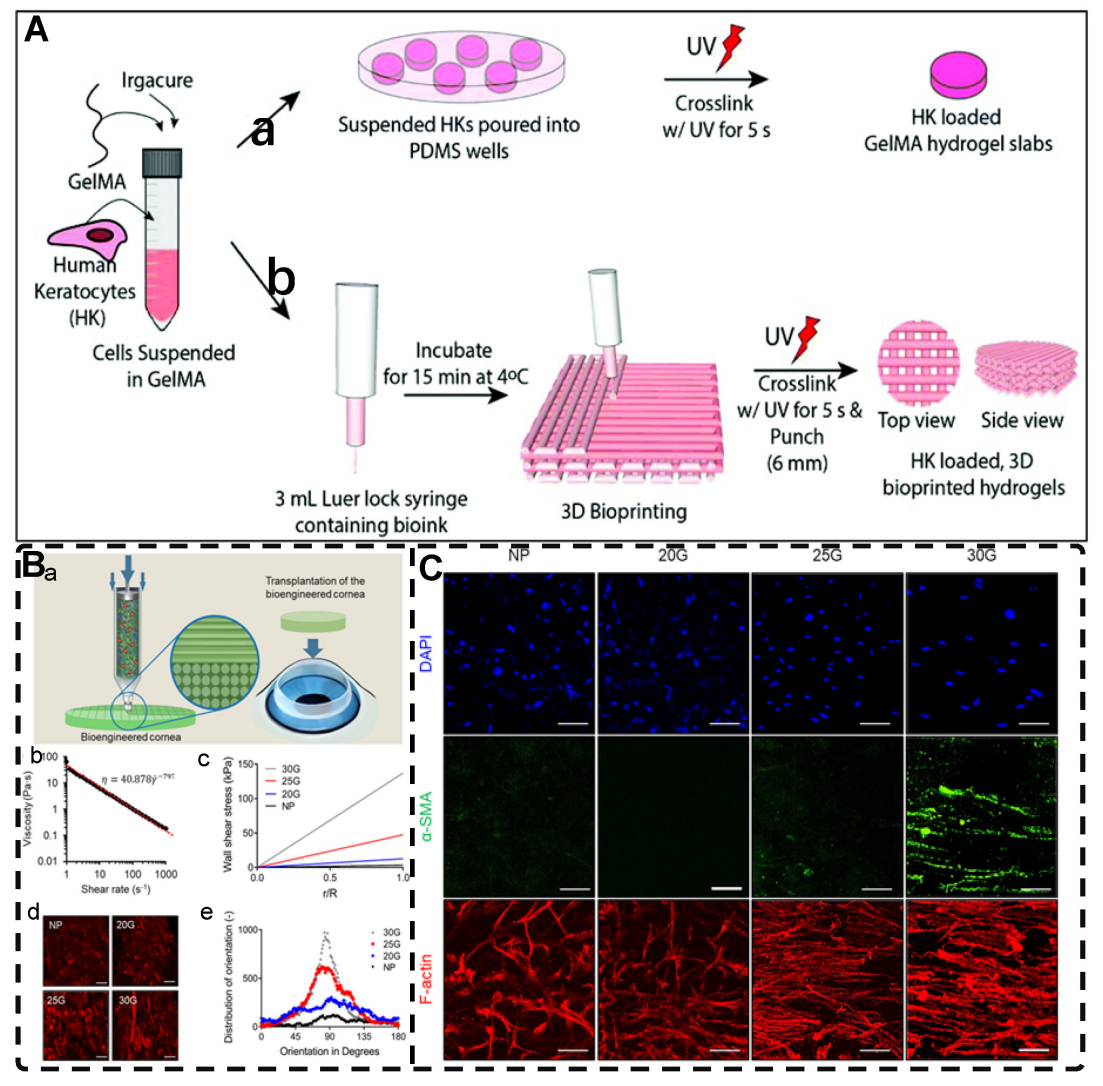
A) Schematic representation of preparation of human keratocyte (HK) loaded hydrogels. (a) GelMA slabs, and (b) 3D bioprinted GelMA hydrogels. Adapted with permission from 393, copyright 2019, Royal Society of Chemistry. B) (a) Schematic illustration of the alignment of collagen fibers within the nozzle during bioink extrusion. (b) Viscosity profile of 2%-Co-dECM bioink incorporating cells. (c) Radial distribution of wall shear stress upon 3D printing for the different-sized nozzles. (d) Second harmonic generation (SHG) images of shear-aligned collagen. (e) Distributions of orientations obtained by analysis using Orientation-J of SHG images collected at different azimuthal angles. C) Cellular behavior of the differentiated keratocytes encapsulated in the bioink on the 28th day of cell culture. Adapted with permission from 394, copyright 2019, IOP Publishing.

The cornea, which serves as the outermost layer of transparent and protective soft tissue in the eye, possesses a hierarchically oriented structure that facilitates the transmission and refraction of light to the retina 17, 391. Comprised mainly of type I collagen, the cornea is divided into three components: the epithelium, stroma, and endothelium 392. Each component consists of oriented collagen fibers embedded within a hydrated proteoglycan rich gel matrix, which provides support for the fibrillar collagen structure. Bektas et al. 393 fabricated 3D printed GelMA hydrogels with an oriented structure that closely replicate the biological and physical characteristics of the corneal stroma** (Figure 22A)**. By optimizing printing conditions, including the nozzle speed in the x-y direction and the spindle speed, they achieved a biomimetic structure comprising parallel fibers within each layer, with adjacent layers arranged perpendicular to one another, mirroring the natural structure of the stroma. Kim et al. 394 utilized the shear forces during the 3D printing process to prepare orientation-aligned collagen hydrogels using a bioink derived from decellularized ECM of the corneal stroma **(Figure 22B)**. The spatial orientation of collagen fibers was manipulated by adjusting parameters like nozzle diameter and flow rate** (Figure 22C)**. In vitro cell cultures demonstrated the expression of keratocyte-specific genes, including keratocan (KERA) and aldehyde dehydrogenase (ALDH). Additionally, the in vivo corneal injury model cultures conducted on New Zealand white rabbits exhibited a homogeneous cell alignment that resembled the natural structure of the human cornea. He et al. 395 employed DLP technology to fabricate a cell-laden bi-layer construct that encompassed an epithelium layer loaded with rabbit corneal epithelial cells and an orthogonally aligned fibrous stroma layer loaded with rabbit adipose-derived mesenchymal stem cells (rASCs). The intention was to mimic the microstructure and microenvironment of the natural cornea. In vivo experiments utilizing a rabbit anterior lamellar keratoplasty (ALK) model effectively facilitated corneal regeneration by promoting efficient re-epithelialization and stromal regeneration. This approach established a favorable topographical and biological microenvironment for corneal regeneration.

### 4.8. Highly Oriented Hydrogels for Vascular Tissue Engineering Applications

The vascular network plays crucial biological roles in the human body, including facilitating nutrient and gas exchange, removing metabolic waste, and maintaining internal environmental homeostasis 396. Furthermore, the formation of the vascular network is accompanied by cellular behaviors that contribute to the growth and development of tissues and organs, as well as various biological events 397. Blood vessels comprise an inner layer of endothelial cells aligned along the long axis of the vessel that is surrounded by smooth muscle cells aligned circumferentially relative to the long axis of the vessel 34. This specific orientation is crucial for preserving the vessel's constant tone and elasticity 38.

**Figure 23:**
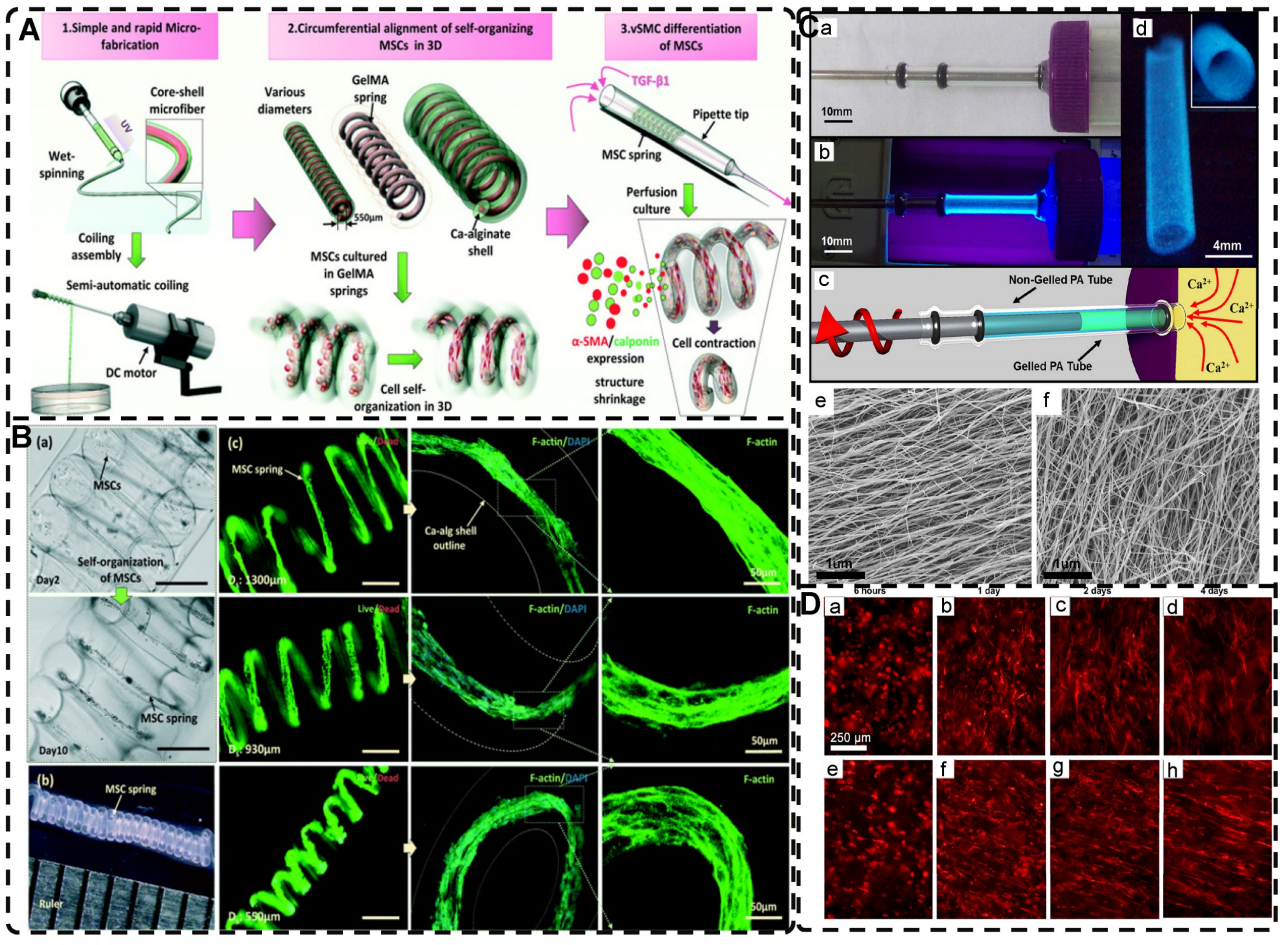
A) MSCs encapsulated in GelMA springs self-organized into smooth muscle-like tissues with cell alignment and contractile property similar to that of vSMCs in the media layer of blood vessels in vivo. B) Formation of MSC springs. (a) Self-organization of MSCs in a GelMA spring. (b) An MSC spring with a large length-to-diameter ratio of about 13.33. (c) Live/dead staining images showing cell viability in the resulting MSC springs encapsulated in GelMA springs with three different diameters. Adapted with permission from 408, copyright 2020, Royal Society of Chemistry. C) Schematic of fabrication device with resulting macroscopic tubular gel. (a) Empty shear chamber assembled and (b) PA solution loaded in shear chamber. (c) Fabrication procedure showing the inner rod's combined rotation and retraction movement allowing the Ca^2+^ solution to flow into the lumen of the tube. (d) Macroscopic photo of final PA tube retaining its tubular shape. The aligned sample (e) shows noticeably more aligned nanofibers than the non-aligned sample(f). D) Cellular alignment viewed from inner surface with the tube's long axis in the vertical direction; (a-d) Non-aligned samples, (e-h) Aligned samples. Adapted with permission from 409, copyright 2012, Elsevier.

Vascular diseases, particularly those affecting vessels with a diameter less than 6 mm, have a high global prevalence 398. While autologous vessel transplantation is the preferred option for revascularization, over 30% of patients do not have an available vessel for use, highlighting the need for tissue-engineered artificial vessels as an alternative graft source in vascular replacement 399. Despite the progress in this field, numerous bionic hollow duct hydrogels have been developed using 3D printing or template-sacrificing techniques 400-407. However, there is a need for more precise strategies to replicate the natural vascular morphology for effective TE applications. Replicating the circumferentially oriented alignment of vascular smooth muscle cells remains a crucial challenge in vascular TE. Sun et al. 408 introduced a GelMA hydrogel-based circumferentially oriented spring structure formed through semi-automated coiling of microscale nucleus-shell microfibrils **(Figure 23A)**. This structure was subsequently utilized as a perfusion culture device to induce the differentiation of human MSCs into contractile vascular smooth muscle cell (SMC) -like cells **(Figure 23B)**. McClendon et al. 409 devised liquid crystalline solutions containing peptide amphiphile nanofibers to create gel ducts with fibers arranged in a circular orientation **(Figure 23C)**. The SMCs were successfully encapsulated during the preparation process, preserving their viability, and exhibited parallel alignment with the fibers, effectively mimicking the circumferential arrangement observed in natural arterial vasculature **(Figure 23D)**.

Replicating the multilayered oriented structure of blood vessels is a key objective in vascular TE research. Bosch-Rué et al. 34 utilized a triple coaxial nozzle to fabricate a bilayer hydrogel that mimics the native vascular structure. The inner hydrogel layer was loaded with HUVECs, while the outer hydrogel layer was loaded with human aortic smooth muscle cells (HASMCs). The injection speed was adjusted to control the size of the simulated vessels. The HUVECs and HASMCs were aligned parallel and perpendicular to the orientation of the bilayer hydrogel, mirroring the natural arrangement of vascular endothelial cells and smooth muscle cells, respectively. This approach presents a novel strategy for creating artificial blood vessels; however, additional experiments involving perfused cultures are necessary to ensure optimal cell alignment and promote the maturation and functionality of the constructs. The implementation of this multichannel coaxial extrusion system (MCCES) holds significant promise for fabricating multilayered tubular biological tissues, including blood vessels 410.

### 4.9. Highly Oriented Hydrogels for Skin and Disc Tissue Engineering Applications

**Figure 24:**
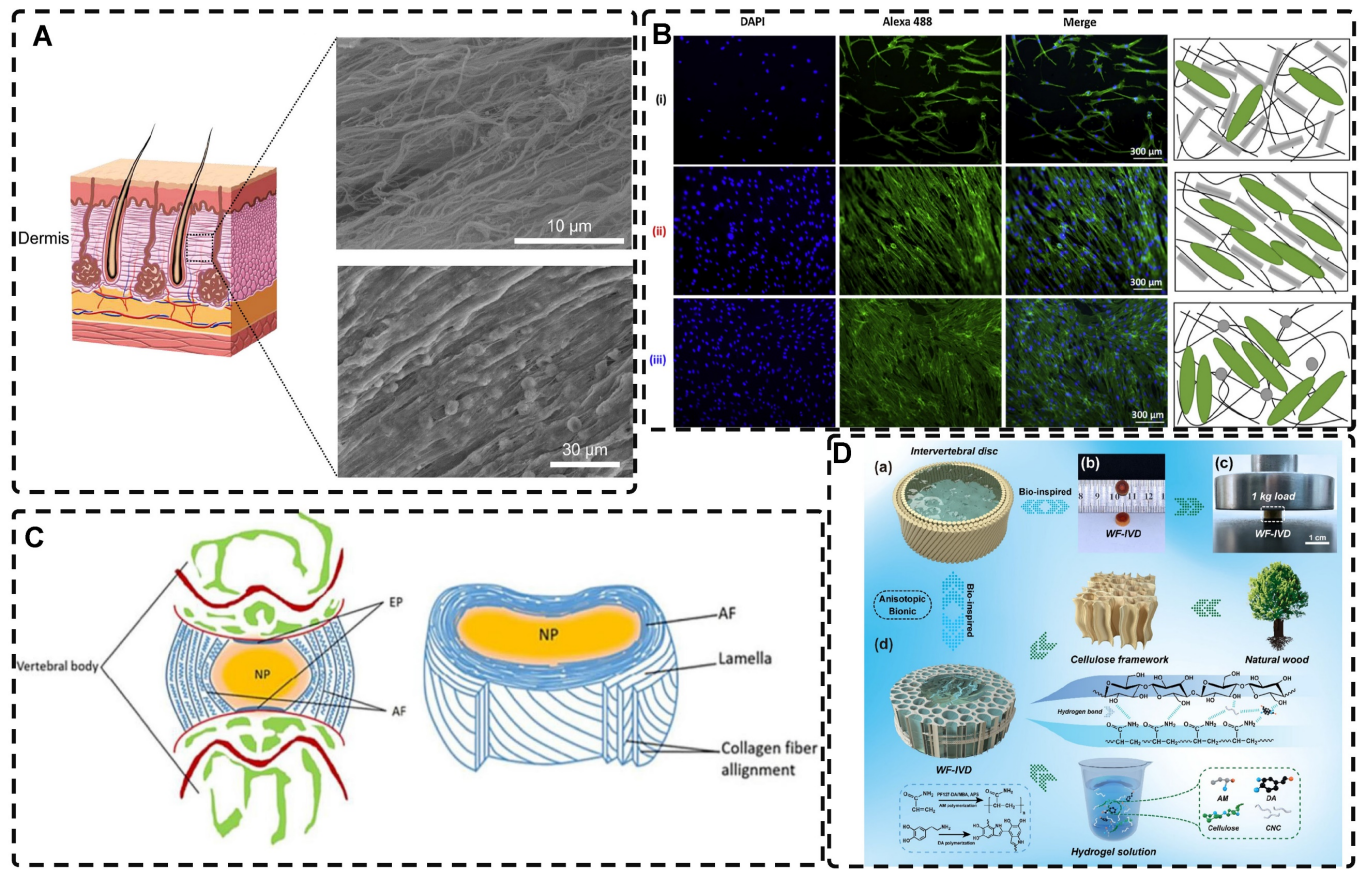
A) A graphical illustration of dermis in skin, and SEM image of aligned collagen fiber bundles observed in the dermis of neonatal rat skin and aligned dermal fibroblasts in the same tissue. The orientation of bundles agrees with the tension line in skin. Adapted with permission from 286, copyright 2012, Elsevier. B) Fluorescence microscopy images and schematic representation of NHDFs culture on highly oriented hydrogels after 48 h. Adapted with permission from 81, copyrights 2020, Elsevier. C) Schematic representations of the adult IVD and midsagittal cross-section showing anatomical regions. Adapted with permission from 414, copyright 2020, Frontiers Media. D) Schematic illustration and fabrication process of WF-IVD. (a) Schematic diagram of a typical intervertebral disk to be simulated by WF-IVD. Images of WF-IVD (b) in different directions and (c) under a 1 kg load. (d) Two-component composite fabrication process. Adapted with permission from 418, copyright 2021, American Chemical Society.

The skin, being the body's largest organ, serves as the outermost layer that provides a physical barrier and regulates body temperature 411, 412. Skin injuries can self-heal, but this depends on the severity or thickness of the wound 413. Managing chronic and full-thickness wounds remains a significant challenge. Limited research has been conducted on reconstructing the oriented structures of the mammalian skin, such as a variety of ECM fibers, including collagen fibers and fibroblasts **(Figure 24A)** 286, and currently, one of the goals in skin TE is to induce fibroblasts to reorient structures resembling natural dermal tissue during dermal injury healing. Shi et al. 81 demonstrated that a magnetic field-oriented 1D SiO_2_@Fe_3_O_4_ rod composite hydrogel could successfully guide the oriented and aligned growth of normal human dermal fibroblasts **(Figure 3B, 24B)**. In the future, the integration of oriented hydrogels with biological cues, such as stem cells or bioactive molecules, needs to be researched to advance the development of ideal tissue-engineered skin substitutes.

The natural intervertebral disc (IVD) primarily comprises two distinct structural tissues: the highly hydrated gelatinous nucleus pulposus (NP) situated at the center and the closely surrounding fibrous annulus (AF) **(Figure 24C)** 414. The NP comprises a reticular fibrous structure and a proteoglycan matrix that exhibits properties of an elastic gel. The AF, which envelops the NP closely, primarily comprises a layered arrangement of type I collagen fibers that maintain the position and shape of the NP, transmit its stresses, and safeguard the integrity of the IVD against bending, stretching, and torsional damage 415. Damage to the NP and AF can result in various degenerative spinal diseases and considerable morbidity 416, and current clinical treatments primarily focus on symptom management. The performance of current artificial IVDs does not align with the biomechanical properties of natural tissue, making it impossible to fully replace the degenerated IVD and restore its complete function 417. Therefore, the development of TE natural discs is crucial, ideally using hydrogels with properties that closely mimic the properties of the IVD. Highly oriented hydrogels present promising strategies for simulating and constructing complex IVDs. For instance, Liu et al. 418 demonstrated the development of a biomimetic IVD through the combination of a naturally oriented cellulose framework with nanocomposite hydrogels **(Figure 24D)**. These hydrogels encompassed Cellulose PAM-Polydopamine (PDA) Composite Hydrogels (CCHs) that mimic the NP, as well as Wood Framework Hydrogels (WFHs) that emulate the AF **(Figure 24D)**. The formation of robust hydrogen bonds among the multi-hydrogen bonded substances (e.g., cellulose (hydroxyl), CNC (hydroxyl), PAM (amino group), and PDA (phenolic hydroxyl)) ensures stable bonding and endows the composite structure with exceptional mechanical properties. The isotropic CCH exhibited a distinct, highly elastic behavior that promoted significant recovery (up to ∼5% energy dissipation) and achieved favorable mechanical matching with the NP tissue. Additionally, the highly oriented WFH offered notable mechanical buffering and energy absorption (at a minimum of ∼60% energy dissipation), akin to the characteristics observed in natural AF tissue. Furthermore, the composite, oriented hydrogel comprised biocompatible materials. Further research is needed to explore additional applications of highly oriented hydrogels in the field of IVD TE.

## 5. Summary and Outlook

Highly oriented structures are crucial for the diverse functions in tissues and organs. Researchers are actively exploring TE using highly oriented hydrogels that mimic the properties of native tissues and organs to varying extents. These hydrogels exhibit characteristics such as aligned structures, anisotropic mechanical properties, and electrical conductivity. This approach is key in enhancing tissue repair, regeneration, and potential replacement, especially in addressing challenging tissue diseases and injuries. Critically, ignoring the differences in enhanced functionality of different materials, highly oriented structures are superior to randomly oriented structures for biomimetic tissue engineering in the same material, including the modulation of cellular behaviors such as adhesion, migration, proliferation, and differentiation, and the provision of a biomimetic in vivo 3D microenvironment for cells. Significant advances in biomanufacturing technologies offer immense potential for the fields of TE and regenerative medicine. This article reviews the application of hydrogels with highly oriented structures in the field of TE and demonstrates their potential in different TE fields and their effects on cell behavior. Despite the promising application of highly oriented hydrogels in TE, there remain challenges that scientists should overcome:

i. Biocompatibility and safety are crucial considerations for hydrogels intended for TE applications. They should not only exhibit biocompatibility with cultured cells in vitro but also ensure a low risk of severe adverse effects such as direct cytotoxicity, immune reactions, or the release of toxic substances, including metabolites, upon implantation into human tissues. Consequently, researchers must prioritize the investigation of biocompatibility and the fate of highly oriented hydrogels in vivo. Additionally, strategies utilizing external stimuli like magnetic fields, electric fields, and shear forces can strongly influence cell survival, while the zero-degree environment during directional freezing can impact cell viability. These limitations present opportunities to enhance or mitigate the impact of these techniques on cell viability.

ii. Improving the mechanical properties of hydrogels is needed as currently, hydrogels are only capable of simulating the mechanical characteristics of soft tissues, and achieving a Young's modulus of up to 20 GPa, comparable to that of bone tissues, remains challenging. Furthermore, fully replicating the mechanical properties of biological tissues means also replicating the elasticity, rigidity, hardness, toughness, fracture toughness, and fatigue strength. Notably, strategies like 3D printing, which enable the fabrication of high-resolution bionic microstructures, face challenges in reconciling the mechanical properties required to ensure adequate flexibility, particularly for tendons and ligaments. A combination of multiple strategies may improve the shortcomings of individual systems. In addition, a potential challenge lies in the bio-adhesiveness of hydrogels for their in vivo implantation due to their insuturability.

iii. Simple high-orientation structures are inadequate as human tissues and organs are highly diverse and complex in their hierarchical organization. Moreover, the highly oriented structures between different layers are connected together by tissue-specific ECM to form an organic whole with a smooth transition in structure and properties, exemplified by skeletal muscle fibers wrapped by extracellular connective tissue or cornea tissue arranged orthogonally. Certain highly oriented hydrogels successfully mimic hierarchically oriented structural tissues like articular cartilage, and this change in orientation between layers holds promise for advancing the understanding of cell behavior at interfacial boundaries. Nevertheless, fully replicating the intricate molecular-to-macroscopic scale of natural tissue structure remains a formidable challenge. Moreover, during the implantation of highly oriented hydrogels into injured tissues, it is crucial to align the tissue orientation with that of the hydrogel to prevent the formation of tissue scars resulting from conflicting orientations.

iv. The precise biological mechanisms by which cells perceive structural orientation in a 3D environment remain incompletely understood. Further investigations of the molecular mechanisms that underlie cellular behaviors, including adhesion, migration, proliferation, and differentiation in highly oriented hydrogels, are needed to enhance the efficiency of engineering manufacturing and propel advancements in TE. Currently, the majority of studies investigating the impact of oriented structures on cell behavior are focused on the 2D environment, which differs to some extent from the 3D environment within hydrogels and the human body. Consequently, the molecular biological mechanisms underlying cell responses in the 2D environment can only serve as a reference for the 3D cellular microenvironment and cannot be directly extrapolated.

v. Human tissues and organs possess not only highly oriented structures but also a diverse array of biophysical cues and biochemical signals that collectively orchestrate the development from individual cells to fully formed tissues and organs. Incorporating complex, highly oriented structures along with many other cues into hydrogels in vivo represents a promising approach in TE to mimic the natural tissue environment better. For instance, the in vitro maturation and differentiation of cardiomyocytes, nerve cells, and skeletal muscle cells can direct cell phenotypes by responding to a range of biochemical and physical cues, including growth factors immobilized in hydrogels, electrical stimulation, and mechanical forces. However, the investigation into coordinating multiple guiding cues and elucidating how cell behavior is modulated in response to diverse cues is still in the early stages.

vi. The pursuit of hydrogels that accurately replicate the structure and function of complex tissues can result in a complexity of design strategies, rendering them unsuitable for efficient industrial production. Additionally, laboratory biofabrication tools are susceptible to flaws, potentially resulting in variations between batches of hydrogels in terms of their desired structure, mechanical properties, compositional uniformity, and so forth. Such inconsistencies can significantly impede product translation and pose challenges during the regulatory approval process. Technologies such as 3D/4D printing provide the possibility to realize simple large-scale production preparation, but the premise is to solve their own limitations.

Numerous challenges and obstacles remain to be addressed in order to attain the ultimate objective of transforming highly oriented hydrogels into finished products. Considering the remarkable progress in biomaterials science, engineering, and cell biology, we anticipate forthcoming captivating advancements in the field of TE.

## Figures and Tables

**Table 1:** Mechanical properties and applicable clinical applications of traditional hydrogels and highly oriented hydrogels.

	Highly Oriented Hydrogel	Traditional Hydrogels
Properties	• A special type of hydrogel, based on the distribution of internal components • With highly oriented structure (Most important properties) • Anisotropic mechanical properties • For special application purposes	• Mechanical property (Comparatively soft materials) • Water-absorbing quality (Wet materials) • Biocompatibility • Biodegradability • Swellability • Stimulti sensitivity (“Smart” materials) • Based on different purposes and applications, special properties will be highlighted
Clinical applications	• Main applications for tissue repair and regeneration of oriented tissues, such as cartilage, bone, skeletal muscle, tendons, myocardium, nerves, blood vessels, and cornea, et al.	• Main applications for tissue repair and regeneration of soft tissues, such as muscle, skin, nerves, blood vessels, et al. • With special mechanical properties enhanced, also used in tissue repair and regeneration of hard tissues, such as bone, cartilage, tendon, et al.

**Table 2:** Comparison of different highly oriented hydrogels and their applications in tissue engineering.

Highly Oriented hydrogel	Strategy	Advantages	Challenges	Applications in tissue engineering
Highly oriented nanofillers hydrogels	• Magnetic-Field-Induced	• Remote control • Nanofillers of different dimensions (0D, 1D, 2D)	• Non-magnetic fillers require high-intensity magnetic fields • Safety of different magnetic fillers in tissue engineering	Tendon 19, 29, 31, Skin 81, Bone 110, Cartilage 309, Nerve 51, 368, 373
	• Electric-Field-Induced	• Fast and easy preparation • Nanofillers of different dimensions (0D, 1D, 2D)	• Noncontact strategy • Electrochemical stability of the material	Cardiac Muscle 129, 130, Nerve 375
	• Mechanical force-Induced	• Fast and simple preparation • Easy processing • Excellent mechanical properties	• Unevenly distributed shear forces • Precise control	Cardiac Muscle 152, 153, Cartilage 152, Tendon 154, 177, Skeletal Muscle 170, Nerve 380, Vessel 409
	• Directed freezing	• Easy processing • Large-scale fabrication	• Time/energy-consuming during freezing • Low temperature environment below zero • Precise control	Nerve 374
	• Self-assembly	• No external force required	• Large-scale fabrication • Easy processing • Precise control	Skeletal Muscle 170, Nerve 390
Hydrogels with highly oriented polymer-chain network	• Mechanical Stretch Induced	• Fast and simple preparation • Easy processing • Excellent mechanical properties • Good reproducibility	• Precise control • Huge mechanical force	Tendon 177, 180, 184, Bone 181, 321, 322, Cardiac Muscle 35
	• Directed Ion Diffusion	• Easy processing	• Non-homogeneous	Vessel 186
	• Shear Force Induced	• Easy processing	• Unevenly distributed shear forces • Precise control	Bone 188, 189, 192, Skeletal Muscle 190, Tendon 190, Nerve 14, 28, 383, Cardiac Muscle 192
	• Electric-Field-Induced	• Fast and easy preparation	• Noncontact strategy • Electrochemical stability of the material	Vessel 194, Bone 195, Nerve 196
Hydrogels with highly oriented void channels	• Directional Freeze-Casting	• Easy processing • Controllable pore size • Controllable pore arrangement • Large-scale fabrication	• Time/energy-consuming during freezing • Low temperature environment • Uncontrollable pore shape	Bone 307, 318, Cartilage 32, 307, 308, Nerve 202
	• physical template/molding	• Easy processing • Personalized assembly	• Low mechanical strength	Nerve 211-215
	• Directed Ion Diffusion	• Easy processing	• Low mechanical strength	Vessel 186
	• 3D Printing	• Precise control • Complex architectures • Rapid prototyping • Scalability • Versatile	• Limited to printable hydrogels • Expensive • Resolution	Nerve 14, 219, Cornea 393
Highly oriented hydrogels with microfabricated structures	• 3D Printing	• Precise control • Complex architectures • Rapid prototyping • Scalability • Versatile • Personalization	• Limited to printable hydrogels • Expensive • Resolution	Skin 25, Skeletal Muscle 192, 346, Cardiac Muscle 192, 355, 357, Tendon 227, 332, Nerve 364, 369, 381, Disc 226, Bone 319, 320, Cornea 394, 395, Vessel 34, 408
	• Molding	• Easy processing • Personalized assembly	• Template's design and prepared	Cardiac Muscle 228, 356, Nerve 231, Skeletal Muscle 231
	• Wrinkling	• Fast and easy preparation • Easy processing	• Precise control	Bone 240
Special highly oriented hydrogels	• Biotemplating	• Excellent mechanical properties	• In vivo degradation	Disc 418, Cartilage Muscle 312, Bone 33
	• Filamented Light biofabrication	• Ultrahigh aspect ratios • Complex architectures • Rapid production	• Expensive	Tendon, Skeletal Muscle 30
	• Embed highly oriented structure in hydrogel	• Personalized assembly	• Complicated	Cartilage 245, 310, 311, Cardiac Muscle 246
